# Immune responses to a HSV-2 polynucleotide immunotherapy COR-1 in HSV-2 positive subjects: A randomized double blinded phase I/IIa trial

**DOI:** 10.1371/journal.pone.0226320

**Published:** 2019-12-17

**Authors:** Janin Chandra, Wai-Ping Woo, Julie L. Dutton, Yan Xu, Bo Li, Sally Kinrade, Julian Druce, Neil Finlayson, Paul Griffin, Kerry J. Laing, David M. Koelle, Ian H. Frazer

**Affiliations:** 1 Admedus Vaccines Pty Ltd (formerly Coridon Pty Ltd), Translational Research Institute, Woolloongabba, Queensland, Australia; 2 University of Queensland, Diamantina Institute, Translational Research Institute, Woolloongabba, Queensland, Australia; 3 Medicines Development Limited, Southbank, Victoria, Australia; 4 Victorian Infectious Diseases Reference Laboratory, Melbourne, Victoria, Australia; 5 Doherty Institute, Melbourne, Victoria, Australia; 6 Q-Pharm Pty Ltd, Brisbane, Queensland, Australia; 7 Department of Medicine and Infectious Diseases, Mater Hospital and Mater Medical Research Institute, Brisbane, Queensland, Australia; 8 The University of Queensland, Brisbane, Queensland, Australia; 9 QIMR Berghofer, Clinical Tropical Medicine Lab, Brisbane, Queensland, Australia; 10 Department of Medicine, University of Washington, Seattle, Washington, United States of America; 11 Department of Laboratory Medicine, University of Washington, Seattle, Washington, United States of America; 12 Vaccine and Infectious Diseases Division, Fred Hutchinson Cancer Research Institute, Seattle, Washington, United States of America; 13 Department of Global Health, University of Washington, Seattle, Washington, United States of America; 14 Benaroya Research Institute, Seattle, Washington, United States of America; IAVI, UNITED STATES

## Abstract

**Background:**

Genital herpes simplex infection affects more than 500 million people worldwide. We have previously shown that COR-1, a therapeutic HSV-2 polynucleotide vaccine candidate, is safe and well tolerated in healthy subjects.

**Objective:**

Here, we present a single center double-blind placebo-controlled, randomized phase I/IIa trial of COR-1 in HSV-2 positive subjects in which we assessed safety and tolerability as primary endpoints, and immunogenicity and therapeutic efficacy as exploratory endpoints.

**Methods:**

Forty-four HSV-2+ subjects confirmed by positive serology or pathology, and positive qPCR during baseline shedding, with a recurrent genital HSV-2 history of at least 12 months including three to nine reported lesions in 12 months prior to screening, aged 18 to 50 years females and males with given written informed consent, were randomized into two groups. Three immunizations at 4-week intervals and one booster immunization at 6 months, each of 1 mg COR-1 DNA or placebo, were administered intradermally as two injections of 500 μg each to either one forearm or both forearms.

**Results:**

No serious adverse events, life-threatening events or deaths occurred throughout the study. As expected, HSV-2 infected subjects displayed gD2-specific antibody titers prior to immunization. COR-1 was associated with a reduction in viral shedding after booster administration compared with baseline.

**Conclusions:**

This study confirms the previously demonstrated safety of COR-1 in humans and indicates a potential for use of COR-1 as a therapy to reduce viral shedding in HSV-2 infected subjects.

## Introduction

Genital herpes, mainly caused by infection with herpes simplex virus (HSV) -2, and sometimes by HSV-1, affects more than 500 million people worldwide. Infection often occurs without or with only mild symptoms and remains unrecognized, which further facilitates transmission. When symptoms occur, individuals experience blisters and/or ulcers, and primary infections can be accompanied by fever, myalgia, and lymphadenopathy. HSV infection remains life-long as the virus establishes latency in the sacral ganglia. During reactivation of the virus, infected individuals often experience outbreaks of increased viral shedding, blisters and ulcer formation which can be painful [1]. HSV-2 infection also increases the risk of HIV-1 acquisition 3-fold [2, 3], and given that HSV-2 infection often remains subclinical, its global impact is likely underestimated [4]. Current therapy relies on antiviral medication such as the nucleoside analogue acyclovir, which reduces the frequency of viral shedding and outbreaks but reduces transmission only by 50% [5]. Further, continuous treatment with anti-viral medication is not attainable in low-income countries with the highest prevalence of HSV and HIV. An effective immunisation-based therapy preventing or reducing HSV-2 viral shedding and spread is desired.

Recent approaches have focused on creating immunotherapeutic vaccines, which induce an effective viral-controlling immune response that is more effective than the naturally elicited one. Several vaccine candidates based on live virus attenuation, viral protein subunits or DNA were evaluated in early clinical trials and their immunogenicity and clinical activity have been reviewed recently [6]. We have previously reported that a codon-modified polynucleotide vaccine COR-1 protected mice in a lethal HSV-2 challenge [7] and was found safe and well tolerated in a phase 1 trial of healthy volunteers [8]. COR-1 also induced cell-mediated immune responses in the majority of subjects.

COR-1 is a DNA vaccine consisting of two codon-modified and optimized plasmids for enhanced expression and immunogenicity upon intradermal delivery in mammals. One plasmid encodes the full length of the envelope glycoprotein D of HSV-2 (gD2) and the other encodes a truncated version of gD2 fused to an ubiquitin sequence, which targets gD2 to the proteasome and ultimately to MHC class I presentation and induction of a CD8^+^ T cell response [7]. Using two different disease models, we have shown that this novel vaccine design induces a balanced adaptive humoral and cell-mediated immune response in mice [7, 9].

Primary objective of the current placebo-controlled, randomized double-blind phase I/IIa trial was to evaluate if COR-1 is safe and well tolerated in HSV-2 positive subjects. Secondary objectives were to test if COR-1 induces cell-mediated and humoral immune responses in HSV-2 positive subjects. Lastly, exploratory objectives were to investigate if immunization with COR-1 leads to reduction in viral shedding and outbreaks and if immunization with COR-1 to two forearms targeting two sets of draining lymph nodes results in stronger responses compared to COR-1 delivery to one forearm.

We report that immunisation with COR-1 was well tolerated and safe in HSV-2 positive subjects. Although the study was not powered to detect significance in changes due to the low number of participants, especially in the placebo group, we observed trends of increases of gD2-specific antibodies and cell-mediated immune responses measured in peripheral blood, and decreases in viral shedding rates post-booster administration compared with baseline in subjects treated with COR-1 but not in those who received placebo. Further optimization of the vaccine is warranted to improve therapeutic efficacy.

## Methods

### Ethics statement

Subjects had given voluntary written informed consent. This clinical trial was approved by the human research ethics committee of the QIMR Berghofer Medical Research Institute (QIMR HREC P922 and P2079). The study was registered on the Australian New Zealand Clinical Trials Registry (ANZCTR): ACTRN12615000094572.

### Subjects, study design and treatment

The CONSORT (Consolidated Standards of Reporting Trials) recommendations were followed (S1 CONSORT Checklist). Full details of the trial protocol can be found in the Supplementary Appendix (S1 File). This study enrolled the first subject on March 5^th^ 2015 and completed the last subject on January 9^th^ 2017. Potential subjects were screened to assess their eligibility to enter the study. Subjects were eligible if HSV-2 infection was confirmed by positive HSV-2 serology using an indirect chemiluminescence immunoassay to HSV-2 gG2 (LIAISON^®^, Murrieta USA), displayed history of recurrent genital HSV-2 for at least 12 months with at least 3 but no more than 9 reported occurrences in 12 months prior to screening (S1 Table), were aged 18–50 years, were male or non-pregnant and non-nursing females (S2 Table). Furthermore, subjects had to have one positive qPCR for HSV-2 during a 45-day baseline shedding assessment by daily swabbing prior to randomization. In addition, the subjects were required to have adequate venous access to allow collection of blood samples; no birthmarks, tattoos, wounds or other skin conditions on the forearms or around the site of immunisation.

This was a randomized single site, double blind, placebo-controlled, parallel group study conducted by QPharm Pty Ltd in Brisbane, Australia. The study was divided into four stages: screening period, study period 1, study period 2 and a follow-up period (visual study timeline in S1 Fig, S3 Table). In the screening period, subjects underwent 45 days of genital swabbing. Three doses of vaccine or placebo was given at 4-week intervals in study period 1 week 0, week 4, and week 8. A booster was administered in study period 2 at week 24. There were two groups in this study. Group 1 subjects received one immunisation into each forearm, and group 2 subjects received two immunisations into one forearm. Once group 1 was fully allocated, group 2 recruitment commenced. Within each group, 22 subjects were randomized to either treatment or placebo in a ratio of 3:1. Group 1 subjects received one immunisation of 500 μg of COR-1 or placebo to each forearm and group 2 subjects received two immunisations each containing 500 μg of COR-1 or placebo in the same forearm. Each dose was administered by authorized clinical site staff experienced in intradermal immunisations. The site of immunisation was an area of skin 5–10 cm below the elbow joint. Subjects in group 2 received both immunisations in the same forearm approximately 5 cm radially from the first immunisation site. If the immunisation was done correctly, a “bleb” or “raised wheal” became visible in the skin. The protocol required visible technical failures within 15 minutes after immunisation (such as no bleb formation or leakage) to be recorded. No technical failures were recorded. Subjects were instructed not to touch or scratch the immunisation site. Blood samples for antibody detection were collected before treatment at week 0, on weeks 12 and 24 post-treatment, and week 28 post-booster period (S3 Table). Blood samples for detection of cellular immune responses were collected before treatment at week 0, on weeks 9 and 24 post-treatment, and week 25 post-booster period. An optional skin biopsy from the site of immunisation was collected 48 hours after the third immunisation. Viral shedding testing was scheduled for 45 days in three periods: before initial immunisation, post immunisations and post booster. Multiple swabs at different genital/anal locations were conducted by each subject on each scheduled study day. Male subjects were instructed to obtain swabs from penile skin and perianal or rectal areas, and female subjects from the vagina or vulva and perianal or rectal areas. Swabs collected from all relevant locations were individually stored in collection tubes and sent back to the clinic for analysis. When a recurrence appeared, subjects were instructed to continue swabbing relevant areas each day until the lesions had healed. If any swab was reported as HSV-2 positive by PCR, the day was counted as positive for viral shedding. Reports of adverse events and outbreaks were recorded throughout the study.

### Study vaccine and placebo

The formulation of the HSV-2 DNA vaccine COR-1 was published previously [8]. COR-1 was manufactured under Good Manufacturing Practice (GMP) conditions by VGXI Inc. (Texas, United States) under license from Admedus Vaccines Pty Ltd (lot COR-1.15C004). The vaccine was supplied frozen and upon receipt, the vials were stored at -20 ± 5^°^C. Each 2-ml sterile glass vial contained a 1:1 mixture of two DNA plasmids (COR-1A and COR-1B) formulated with 10 mM (hydroxymethyl) animo methane hydrochloric acid (Tris HCl) and 1 mM ethylenediamine tetra-acetic acid (EDTA) at pH 8 and was sealed with Teflon coated butyl stoppers and aluminum crimp caps. Each COR-1 single use vial contained 1250 μg in 0.5 ml of buffer. All doses administered in the study were prepared by authorized staff at the clinical site pharmacy according to instructions provided by Admedus Vaccines Pty Ltd. Sterile, isotonic and endotoxin-free TRIS-EDTA (TE) buffer, manufactured under GMP condition by Sypharma Pty Ltd (Victoria, Australia) was used as placebo (lot BP-008). Placebo vials were stored at -20 ± 5^°^C.

### Randomization and blinding

Independent pharmacists dispensed either active or placebo doses according to a computer generated randomisation list. The generation of the randomisation list for each group was performed by a clinical network service (CNS) biostatistician using SAS® v9.4 software. For each group, a set of sealed, opaque, tamper-evident individual codebreak envelopes were prepared and provided to the site. The individual codebreak envelopes were labelled as “Individual Codebreak Information” and visibly displayed (without requiring opening of the envelope) the protocol number, name of principal investigator, and randomisation number. The sets provided to the investigational site were stored in a secure accessible location.

Q-Pharm pharmacist and delegates were unblinded. All other investigator site staff, sponsor and participants were blinded to treatment allocation until the completion of the study. No unblinding was required due to the presentation of an adverse event (AE) or serious adverse event (SAE). The unblinded CNS biostatistician, unblinded clinical research associate (CRA) and unblinded Q-Pharm pharmacist had access to the randomisation list and therefore were not blinded to treatment allocation.

### Safety and tolerability assessments

The safety and tolerability of COR-1 across all groups was evaluated according to the following specific assessments: physical examination, clinical laboratory tests, vital signs, ECG, signs and symptoms of tolerability including immunisation site reactions (ISRs) and adverse events (AEs). Subjects were requested to complete an ISR diary for each week following the administration of the vaccine or placebo. Incidence and severity of local reactions (soreness, redness, induration, ecchymosis, edema, itching and paresthesia) at the site of the immunisation was documented. All AEs, regardless of severity, causality or seriousness were to be reported from the date of informed consent until the end of the study or 28 days after the last dose of study medication. The safety assessments were performed immediately prior to each immunisation with vaccine or placebo. All subjects were dosed by study personnel and were required to remain at the clinic for at least 60 minutes after immunisation, or longer if clinically indicated.

Treatment-emergent adverse events were analysed of the safety population. The safety analysis set included all subjects who received at least one injection of study drug. Regardless of their randomized assigned treatment, subjects were included in the treatment group according to the actual treatment received.

### Local tissue response at the site of immunisation

Skin punch biopsies from volunteers were taken from immunisation sites 48 hours after the third immunization (week 8 + 2 days) to assist in determining the type of immune response occurring by the presence of different types of immune cells. To obtain a skin biopsy, a small amount of local anaesthetic was injected under the epidermis. Once the area was numb, a sterile 3 mm skin punch was used to obtain the punch. Pressure was applied, and the device was twisted and gently pushed until the blade of the skin punch pierced the epidermis of the skin. The skin was collected using a forcep and scalpel, and placed in a single container with 4% buffered formalin. Biopsies were sent to TissuPath Specialist Pathology (Victoria, Australia) for fixation, paraffin embedding, sectioned at 5 μm and prepared for H&E staining and immunohistochemistry. Antibodies to test for CD3 (2GV6), CD4 (SP35), CD8 (SP57), CD68 (KR1), CD1a (EP3622), CD45 (LCA RP2/18) were purchased from Roche and IFN-γ (ab89657) from Abcam. For manual counting a 10 μm^2^ gradicule in the microscope eyepiece and 400x magnification resulting in an effective field size of ~25 μm x 25 μm (~625 μm^2^ [0.0006mm^2^]) was used. Up to 10 fields of 100 μm^2^ were counted for each cell type across both dermis and epidermis, and a mean number was calculated per one field.

### Interferon-γ enzyme-linked immunospot assay

Peripheral blood mononuclear cells (PBMCs) were isolated using Ficoll density gradient centrifugation by QPharm and frozen in media containing 10% DMSO in liquid nitrogen. T cell responses were analyzed using peptide pools (gD2-A; 1-139aa; gD2-B: 130-286aa; gD2-C: 259-393aa; Biosynthesis) of 13-mers overlapping by 10 amino acids at 1 μg/mL each spanning the whole length of the HSV-gD2 protein (in triplicate wells) in interferon (IFN)-γ enzyme-linked immunosorbent spot (ELISPOT). Sterile white-walled PVDF membrane bottomed 96-well plates (Cellular Technology Limited) were coated overnight at 4^°^C with IFN-γ specific coating antibody (Cellular Technology Limited). After coating, plates were washed with sterile PBS (GE Healthcare) and complete RPMI containing 10% fetal calf serum (FCS, Hyclone) was added to equilibrate medium in the plates to the appropriate pH and temperature for human PBMC. PBMC were thawed and assessed for cell number and viability before and after an overnight rest in complete RPMI using a Guava easyCyte Flow Cytometer (Millipore). Overnight-rested PBMC were stimulated with medium alone, HSV-gD2 peptide pools, CEF control peptide pools (Biosynthesis), which are a mixture of peptides from cytomegalovirus, EBV, and influenza peptides known to stimulate memory T-cells, or PHA at a cell number of 2 x 10^5^ to 2.5 x 10^5^ PBMCs/well. PBMC control samples from the Koelle laboratory were included in all assays for quality control (QC) measures. The positive control PBMC were chosen based on screening of HSV-2-infected persons to have reactivity to each of the three pools of gD2 peptides. QC tracking of identical sister vials of PBMC from the HSV-2-seropositive control and one HSV-seronegative control donor confirmed there was no temporal change in cell recovery, viability, and immunogenicity measures throughout the laboratory evaluation of the test subjects’ PBMC. PBMCs were incubated with stimulants at 37^°^C and 5% CO_2_ for 18 to 24 hours. Plates were washed twice in PBS/0.05% Tween 20. For detection, detection antibody solution (Cellular Technology Limited) was added, followed by Streptavidin-Alkaline Phosphatase solution (Cellular Technology Limited) and developer solution (Cellular Technology Limited). Plates were dried, and spots were quantitated using CTL-ImmunoSpot S6 Micro Analyzer. Values of SFUs were adjusted to 10^6^ PBMCs/well. SFUs above 30 after background subtraction (medium and DMSO) were considered positive. The presence of both a two-fold increase and a minimum value of 30 for mean triplicate spot forming units (SFU)/well after immunisation compared to baseline was considered treatment-related.

### Antibody responses

Sera taken before immunization (week 0), after immunisation (week 12), before booster immunisation (week 24) and after booster immunisation (week 28) were frozen and shipped to Charles River Laboratories for analysis. Charles River Laboratories Edinburgh Ltd. analyzed the serum samples for gD2-specific antibodies and titers by ELISA. For this assay, plates were coated with an HSV-gD2 recombinant protein which covers the immunodominant region (266-394aa) of gD2 (Meridian Life Science Inc, Cat. R18530). The positive control was a pool of HSV-gD2 positive human serum samples derived from consenting positive patients from a previous HSV-2 study (approval number HREC/12/QPAH/348). The negative control was a pool of HSV-gD2 negative human serum from consenting HSV sero-negative patients taken from a previous HSV-2 study (approval number UQ-2012001301). A HSV-2 seronegative serum pool diluted in assay buffer to minimum required dilution and applied to 12 wells was used to determine a screening cut point (SCP) for each plate. The following formula was used to determine the SCP: SCP _plate_ = mean detector response of negative pool replicates + (1.645 x standard deviation). Antibody titers were reported as the highest dilution factor that produced a mean detector response value greater than the SCP. Each titration sample was analysed on at least 3 occasions for inter-assay precision, and 3 titration samples at each level were analysed on at least one occasion for intra-assay precision.

### Viral shedding

Subjects were requested to provide daily genital skin swabs on 45 consecutive days in three study periods (before immunisation, post immunisation and post booster immunisation). Males collected swabs from penile and perianal/rectal areas and females collected swabs from vaginal/vulva and perianal/rectal areas. Viral shedding was assessed by a qualitative PCR at the Victorian Infectious Disease Reference Laboratory (Melbourne, Australia). The assay was a highly sensitive multiplex TaqMan PCR for HSV-1/HSV-2/Equine herpes virus (EHV), each with specific primers and fluorescent probes. The assay has been National Association of Testing Authorities (NATA) accredited, and is subjected to the Australian (RCPA) Quality Assurance program twice annually. Swab samples in 1 ml Liquid Amies medium (Copan ESwabs) were stored at 4ºC and sent at room temperature by post to the laboratory as 7-day collections. Nucleic acid was extracted from 200 μL of sample using QIAamp 96 Virus QIAcube HT kit (Qiagen Hilden) using QIAcube robotic instrumentation with manufacturers conditions. As a nucleic extraction control and PCR amplification control verifying absence of inhibition EHV DNA was added to each sample to achieve a copy number of approximately 100 copies per PCR reaction. If samples with negative HSV-2 detections fell outside the appropriate range of detection for EHV (cycle threshold 30–32) they were repeat extracted and retested. Nucleic acid extracts were tested using a real-time TaqMan PCR performed on AB7500 Fast instrumentation using default cycling conditions for 45 cycles with Quanta Perfect mastermix (Gene Target Solutions) and primers targeting the glycoprotein B region of HSV-1 and HSV-2 with specific probes labelled with FAM and VIC for HSV-1 and HSV-2 differentiation respectively. The lower limit of detection is approximately 200–400 copies/ml.

Viral shedding was analyzed qualitatively (positive/negative). A subject was counted positive for a day with HSV-2 detected if any of the collected swabs was positive. The shedding rate was described as number of days per year with HSV-2 detected. This number was estimated from the number of days with HSV-2 detected within the 45-day swabbing period. The number of days on which swabs were collected was not adjusted for. However, protocol compliancy was high and ~92% of swabbing periods of subjects who completed the study contained swabs of at least 40 days, and ~98% of swabbing periods contained swabs of at least 30 days. Viral shedding rate for each study period was compared between treatment groups by a random intercept Poisson regression analysis, with number of days with HSV-2 detected in each period as response variable and logarithm of number of days with swabbing conducted in the period as offset variable. The random intercept Poisson regression was used to account for the correlation among repeatedly collected data between study period for the same subject. The overdispersion of data was accounted for by using a robust sandwich estimator of standard error of the estimated viral shedding rate. The Intent to Treat (ITT) data set was used for analysis and included all randomized subjects. Subjects in the ITT set were analyzed according to their randomized treatment group regardless of exposure to study drug or the actual treatment received. If a subject withdrew before the planned end of study, the swabbing days up to the withdrawal date was included in the analysis. Of the total 8 early withdrawals, all 8 subjects missed swabbing data in the post booster period and no shedding rate was calculated (S5 Table). Six of 8 subjects collected no post vaccination swabs, and one subject collected all post vaccination swabs. One other subject collected 5 of 45 swabs and was included in the analysis.

### HSV-2 outbreaks

Subjects were provided with HSV-2 outbreak diaries to record information about their HSV-2 outbreaks. If there was an outbreak, an outbreak diary was recorded for 7 continuous days, with a new diary started for each new outbreak. The number of symptomatic genital herpes outbreaks per year was estimated similar to the analysis method for viral shedding by using a random intercept Poisson regression, with number of outbreaks reported in each period as response variable and logarithm of days of exposure in each period as offset variable.

### Statistical analysis

The sample size was determined on the basis of practical and logistical considerations rather than statistical power with regard to hypothesis testing or precision in parameter estimation. No formal statistical comparisons were planned in the prospective study design. All statistical tests were designed after data collection. Differences in local immune cell infiltration at the immunization site were compared for each parameter using a Mann-Whitney test. Differences between groups in cell-mediated humoral immune responses were calculated using one-way ANOVA. Differences in viral shedding were calculated using random intercept Poisson regression with further details outlined within the viral shedding methods section.

### Independent review of data

CNS (Brisbane, Australia) compiled the data and prepared the clinical trial report. An interim analysis was performed following the booster vaccination in the 20 subjects of group 1, and was conducted for information purposes only. The interim analysis did not affect the type I error level of the final analysis and no multiplicity adjustment was required.

## Results

### Subjects

**Fig 1:**
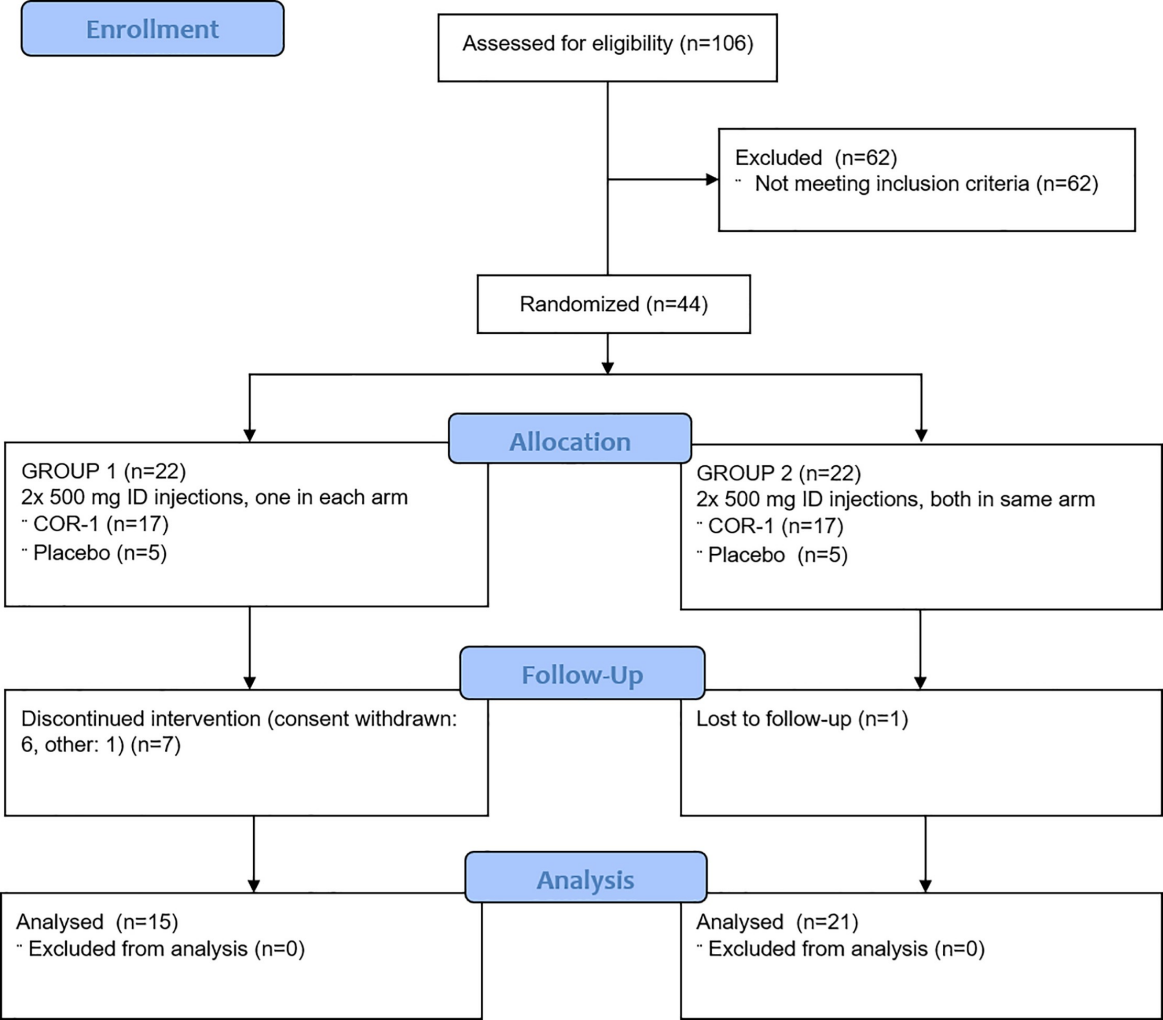
Disposition of subjects. N = number of subjects.

We screened 106 subjects for eligibility, of which forty-four symptomatic HSV-2 infected subjects (aged 18–50) were eligible for participation in the study. The main reasons for exclusion were absence of HSV-2 detection within the baseline screening swabbing period, or a positive HSV-1 swabbing result. Eligible subjects were randomized into two groups of 22 subjects (Fig 1, S2 Table). All subjects received 2 intradermal immunisations. Group 1 received one immunisation into each forearm and group 2 received both immunisations into the same forearm. Recruitment of group 2 commenced once group 1 was fully allocated. Of each group, 17 subjects were immunised with COR-1, and 5 subjects with placebo. Of group 1, seven subjects of the COR-1 immunised cohort withdrew. Six subjects withdrew consent (five for personal reasons such as change of circumstances which prevented them from coming to the clinic, one due to anxiety about the vaccine). One subject withdrew due to unspecified “other” reasons. From group 2, one subject in the placebo cohort was lost to follow-up. Of subjects that withdrew pre-maturely, two subjects withdrew after first immunisation, four subjects after third immunisation and two subjects after booster immunisation. Thirty-six subjects completed the study as per protocol.

### Safety and tolerability of COR-1

No serious adverse events, life-threatening events or deaths occurred throughout the study. All 34 subjects receiving COR-1 and 90% (9/10) of subjects receiving placebo reported treatment emergent adverse events (TEAEs), which were dominantly grade 1 (mild) (97.1% in COR-1 subjects and 100% in placebo subjects). A total of 786 TEAEs were reported, and of these 547 were considered treatment related. The frequency of TEAEs was higher with COR-1 treatment, with 488 treatment-related TEAEs reported by 34 COR-1 dosed subjects and 59 treatment-related TEAEs reported by 10 placebo subjects. The most frequently reported TEAEs were mild systemic symptoms, and administration site erythema, induration, discoloration or pain (S4 Table). No TEAE was considered to be life threatening. One subject receiving COR-1 in Group 1 experienced two non-serious TEAEs, (anxiety and abdominal pain) considered unrelated to immunisation, which led to withdrawal from the study. Adverse events of special interest (AESI) were identified as fatigue, myalgia, malaise, fever, rigors, arthralgia, nausea, diarrhea, light headedness, dizziness, hypersensitivity and headache. The incidence of AESIs was similar between subjects receiving COR-1 (44.1%) and placebo (40%). Altogether, COR-1 was found to be safe and well tolerated by HSV-2 positive symptomatic subjects.

### Local tissue response

**Fig 2:**
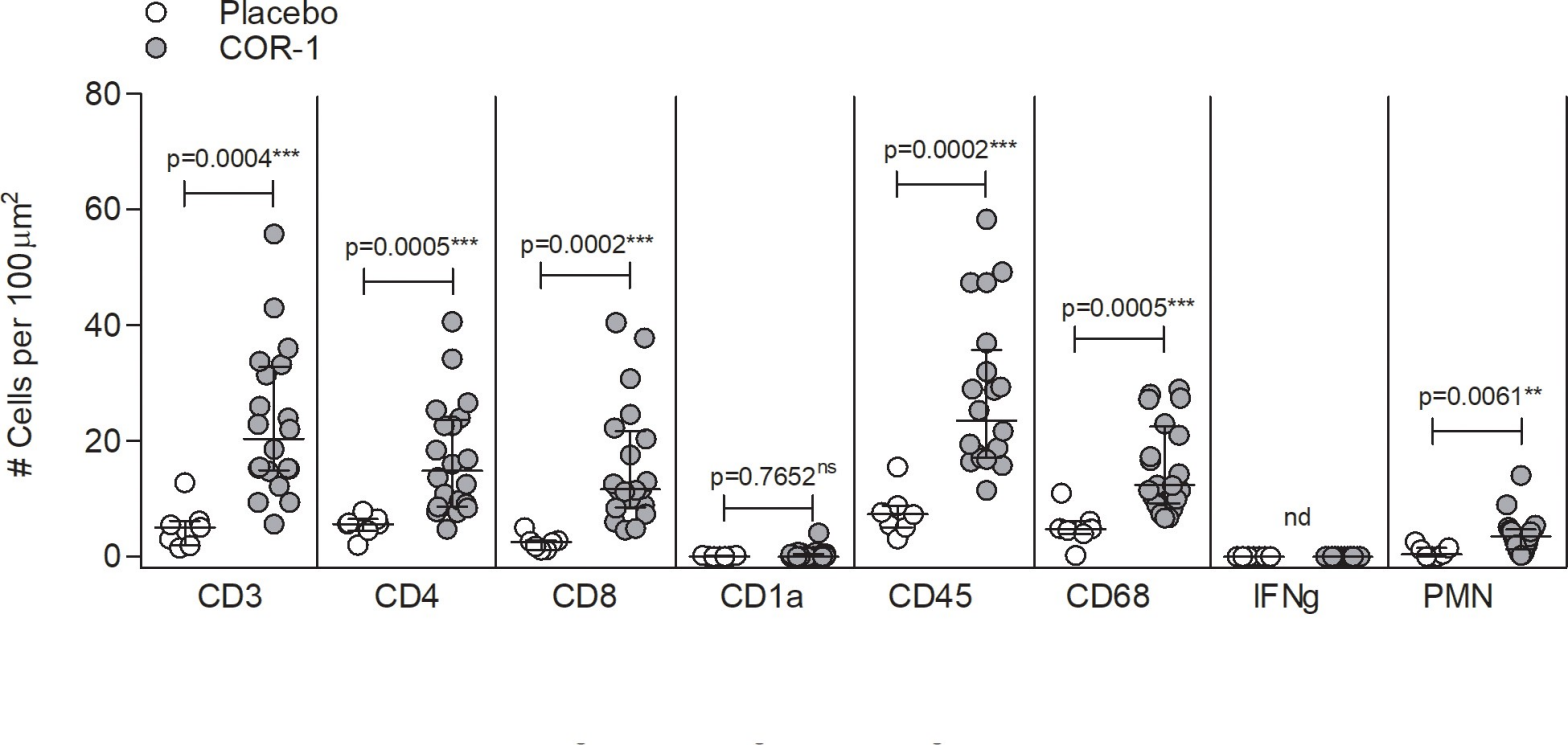
Local tissue response at the site of immunisation. Skin punch biopsies of 27 subjects (20 COR-1 immunised and 7 placebo) were collected 48 hours after the third immunisation and assessed for presence of immune cells by immunohistochemistry. Each data point represents the average number of cells from up to 10 fields counted in one individual (1 field = 100 μm^2^). Median +/- interquartile range (IQR) are indicated. Statistical significance was calculated by Mann-Whitney test. *p<0.05, **p<0.01, ***p<0.001, ns = not significance, nd = not detected.

We assessed the local tissue response at the site of immunisation by analyzing immune cell infiltrates at the immunisation site 48 hours after receiving the third immunisation (Fig 2). We found a highly significant increase in total CD45^+^ leukocytes per 100 μm^2^ (median of cell number per field [interquartile range] COR-1 versus placebo, 23.5 [15.725] versus 7.3 [2.95]), CD4^+^ (14.9 [13.975] versus 5.6 [1.1]) and CD8^+^ T cells (11.6 [12.075] versus 2.5 [1.25]), and CD68^+^ macrophages (12.4 [11.825] versus 4.8 [1.25]) and polymorphonuclear neutrophils (PMN) (3.5 [2.875] versus 0.4 [1.35]) with COR-1 immunisation, when compared to placebo. We were unable to detect IFNγ^+^ cells and numbers of CD1a^+^ antigen presenting cells were very low (<4) or absent. This result suggests that administration of the DNA vaccine stimulated immune cellular activity at the site of immunisation. However, it remains undetermined if this immune cell infiltration was gD2-specific or associated with stimulation of innate immunity by plasmid DNA.

### Antigen-specific humoral and cell-mediated immune response

**Fig 3:**
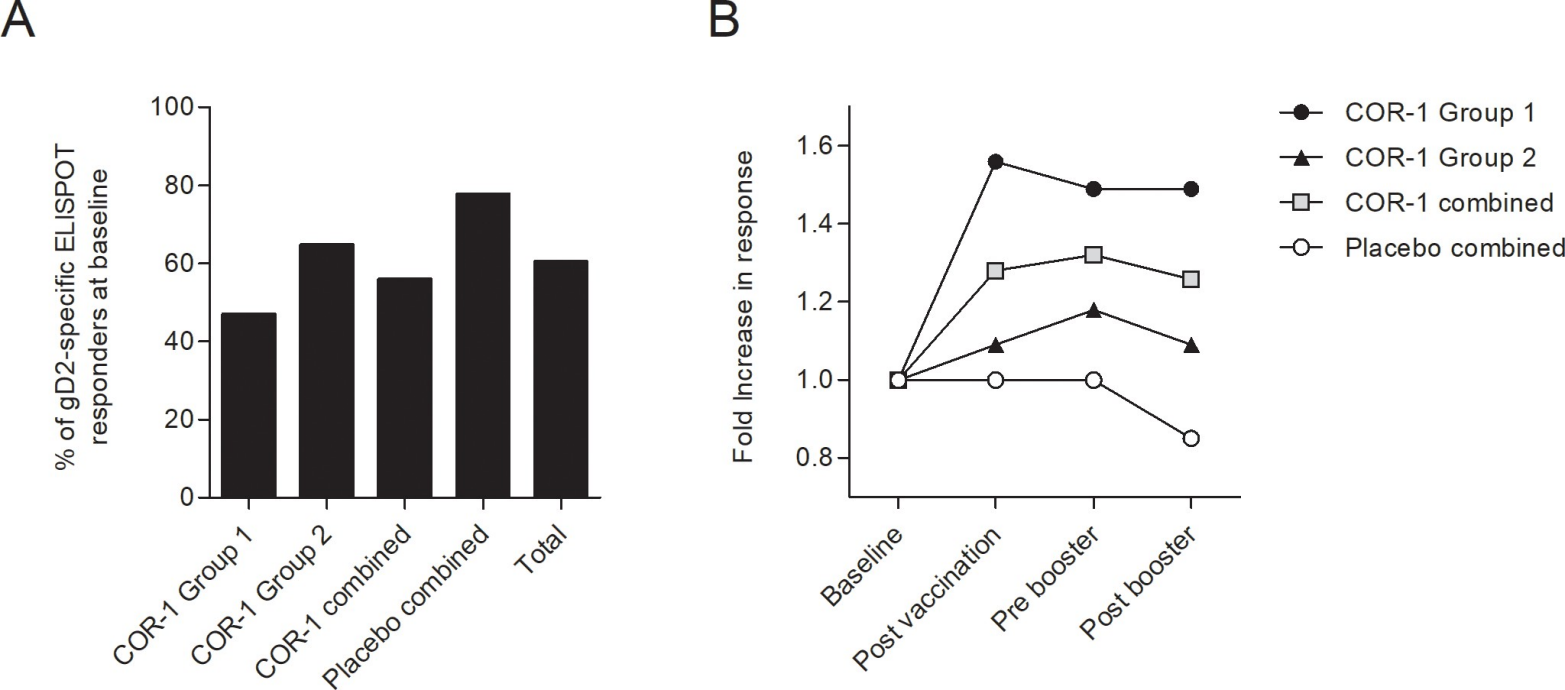
gD2-specific cellular immune responses. Responses to gD2 were determined using PBMCs collected at baseline (week 0), after immunisation (week 9), before booster (week 24) and after booster (week 25). Three pools of overlapping peptides (HSV-gD2 A, B, C) spanning the whole length of gD2 were used to recall PBMC IFNγ ELISPOT responses. Responses presenting as SPUs>30 in any peptide pool were considered positive. The percentage of subjects which displayed more than 30 SPUs in any peptide pool was calculated for each time point. **(A)** The graph shows the percentage of subjects which were positive by criteria SPU>30 at baseline. **(B)** Subsequently, with baseline set to 1, the fold increase in the number of subjects displaying a positive response (SPU>30) was calculated.

**Fig 4:**
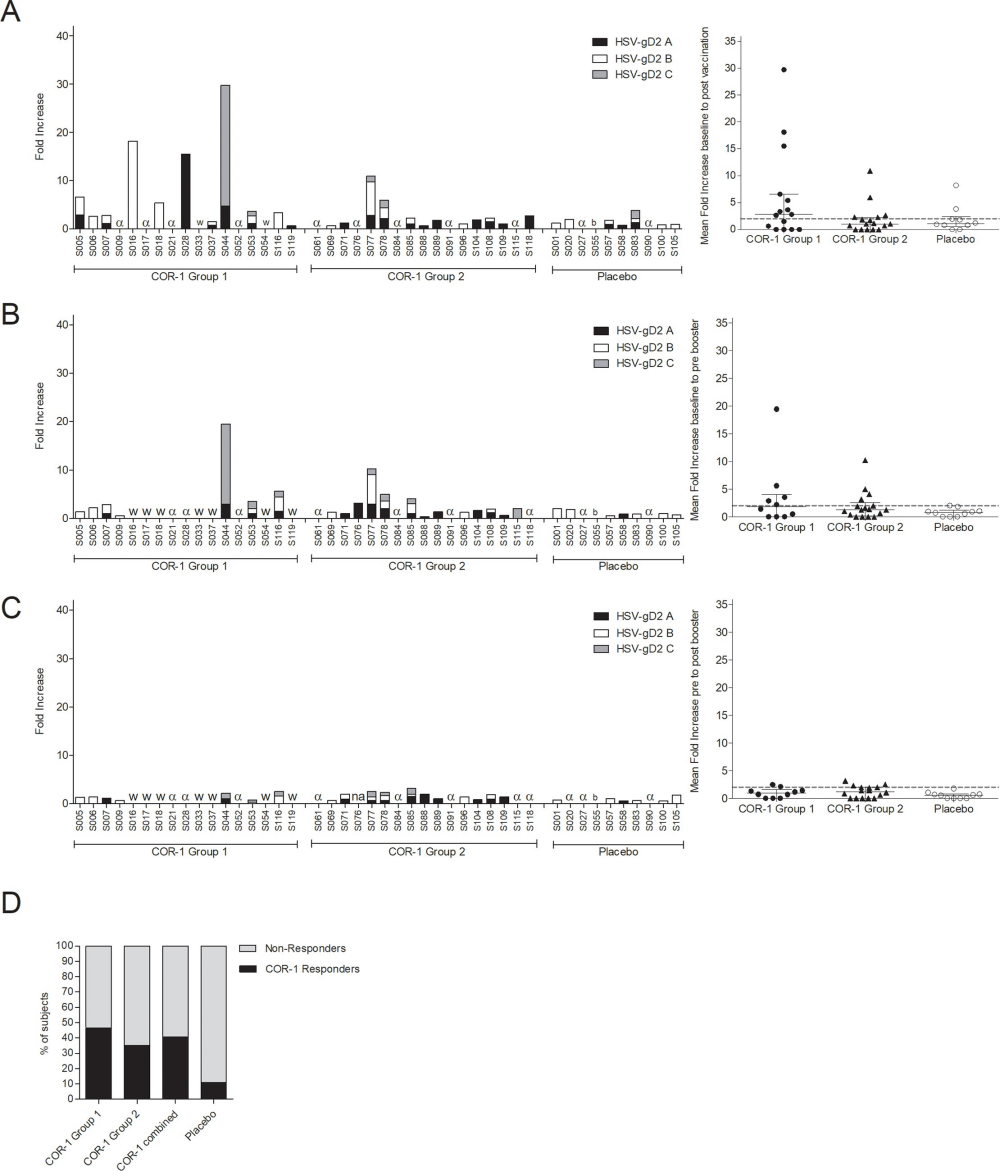
gD2-specific cellular immune responses to COR-1. Responses to gD2 were determined using PBMCs collected at baseline (week 0), after immunisation (week 9), before booster (week 24) and after booster (week 25). Three pools of overlapping peptides (HSV-gD2 A, B, C) spanning the whole length of gD2 were used to recall PBMC IFNγ ELISPOT responses. **(A-C)** Fold increases of spots to all three peptide pools and of all subjects individually (left panel) or averaged with median +/- IQR indicated (right panel) from week 0 to week 9 **(A)**, from week 0 to week 24 **(B)** and from week 24 to week 25 **(C)**. A dotted line at 2 indicates threshold above which subjects were considered as positive responders. Samples with baseline spot forming units below 30 after background subtraction were excluded from analysis (α). Premature study withdrawals are indicated (w). Subject S055 was excluded from analysis due to high background in negative control wells (b). No PBMCs were available from subject S076 at week 25 (na). **(D)** The percentage of subjects considered as responders by criteria SPU>30 and fold increase >2 in any peptide pool at any time point after immunization was evaluated. Responses were determined positive with a minimal fold change of 2 from week 0 to at least one peptide pool.

To assess antigen-specific cellular immune responses to COR-1, we measured the IFNγ response of PBMCs restimulated with three pools of overlapping peptides (HSV-gD2 A, B and C) spanning the complete length of gD2 by ELISPOT. Samples for which exposure to gD2 peptide pools was associated with less than 30 spot forming units (SFUs) per one million PBMCs, after subtraction of background, were considered negative. We found ~60% of all subjects responded positively (SFU>30) to at least one peptide pool at baseline, demonstrating pre-existing cellular immune responses in this HSV-2 seropositive cohort (Fig 3A). When we examined the change in ELISPOT response after immunization in subjects responding positively (SFU>30) to at least one peptide pool at recruitment, we found that the number of positive responders did not increase after immunisation in the placebo cohort, but frequently increased in groups receiving COR-1 (Fig 3B). The number of subjects with a positive response increased more strongly in COR-1 group 1 compared to the number of subjects with a positive response in the COR-1 group 2 (~1.5-fold and ~1.1-fold respectively). We also analyzed the fold change in SFUs across all peptide pools, comparing before immunisation samples with post immunisation, pre-booster immunisation and post booster immunisation. A sample demonstrating less than 30 SFUs after background subtraction after exposure to a peptide pool was regarded as a non-responder for that pool. A subject demonstrating, at any time point after immunization, a minimum of 30 SFUs and a 2-fold increase in SFUs, in response to any peptide pool when compared to the pre-immunisation response to that pool, was considered as a responder to the immunisation. By these criteria, we found that 13 of 32 subjects (40.63%) in the COR-1 cohort of Group 1 and Group 2 had responded, with most responses observed after the third immunisation (week 9) (Fig 4A–4C), whereas only one of 9 subjects receiving placebo (S001) developed a new response (11%). The response to immunisation measured in PBMCs was not further increased with a booster immunisation (Fig 4C). Interestingly, 46.6% of Group 1 subjects receiving immunisation to two arms generated a specific T cell response to COR-1, compared to only 35.3% of Group 2 subjects receiving immunisation to only one arm (Fig 4D). These data suggest that targeting two lymph node sites might be more beneficial in initiating an immune response than targeting only one lymph node site.

**Fig 5:**
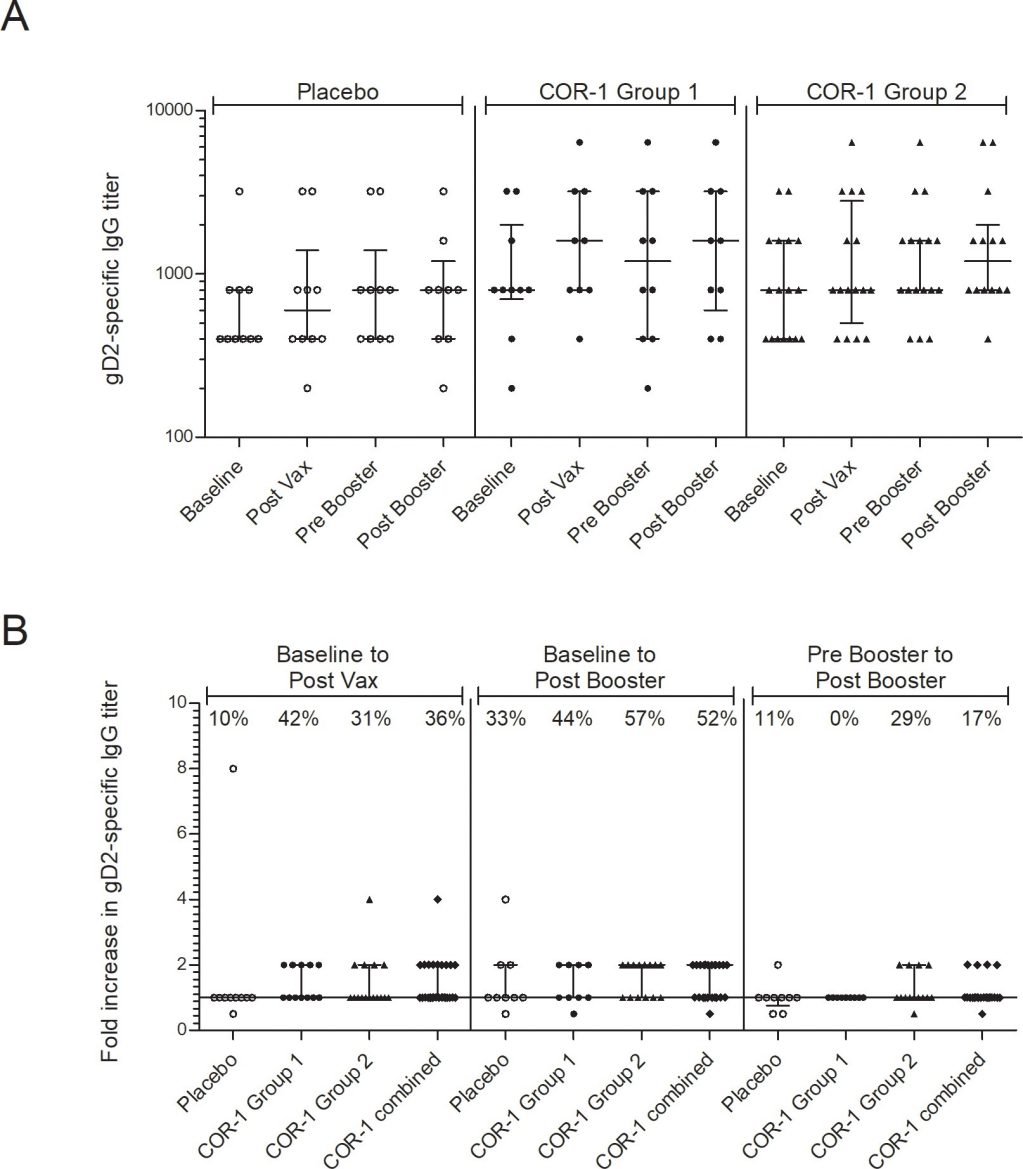
Humoral immune response to COR-1. Serum was collected before immunisation (baseline, week 0), after immunisation (post vax, week 12), before booster (pre booster, week 24) and after booster (post booster week 28). gD2-specific IgG antibodies were detected by capture ELISA and titers were determined by serial dilution. The highest titer which resulted in a mean detector response higher than the plate-specific screen cut point determined using HSV-2 negative pooled sera was reported. **(A)** shows gD2-specific IgG titer and **(B)** the fold increase of titer with each subject represented individually and median and interquartile range indicated. Values with percentile indicate percentage of subjects with a minimum of 2-fold titer increase. No statistical significance was detected between groups using one-way ANOVA analysis.

For evaluation of antibody responses, sera were first screened for positivity by establishing a screening cut point using gD2 seronegative serum. As expected, all subjects enrolled in the study tested positive for gD2-specific IgG antibodies with a range of antibody titers (Fig 5A). After immunisation, we observed a ≥ 2-fold increase in specific antibody titer in ~36% of subjects receiving COR-1 and in 10% of subjects receiving placebo (one subject of ten) (Fig 5B). After the booster immunisation a ≥ 2-fold increase in specific antibody titer was observed in ~52% of subjects receiving COR-1 and in 33% of subjects receiving placebo. However, most antibody increases were minor and overall no statistical significance was detected between placebo and COR-immunized groups (Fig 5A and 5B).

### Outbreaks and viral shedding

Outbreak rates and viral shedding rates at each study period for each treatment, estimated using random intercept Poisson regression, are reported in Tables 1 and 2.

**Table 1:** Outbreak recurrence rates (average number per year) by study period and intervention arm.

	COR-1 Group 1 N = 17	COR-1 Group 2 N = 17	COR-1 combined N = 34	Placebo N = 10
Baseline Number of outbreaks/year (95% CI)	4.63 (2.62, 8.18)	4.72 (2.57, 8.66)	4.76 (3.17, 7.15)	4.44 (2.25, 8.76)
Post vaccination Number of outbreaks/year (95% CI)	2.35 (1.25, 4.39)	1.83 (0.88, 3.82)	2.09 (1.30, 3.36)	2.07 (1.25, 3.43)
Post booster Number of outbreaks/year (95% CI)	1.11 (0.49, 2.53)	1.12 (0.58, 2.14)	1.15 (0.70, 1.88)	1.19 (0.42, 3.38)

Note: Calculated using Poison regression and extrapolation to one year.

**Table 2:** Viral shedding rates (average number and percentage of days with HSV-2 detected per year) by study period and intervention arm.

	COR-1 Group 1 N = 17	COR-1 Group 2 N = 17	COR-1 combined N = 34	Placebo N = 10
Baseline Number of days HSV+/year (95% CI) Percentage of days HSV+ (%)	66.85 (46.34, 96.43) 18.3	70.55 (48.79, 102.02) 19.3	68.53 (53.05, 88.54) 18.8	52.01 (32.09, 84.30) 14.3
Post vaccination Number of days HSV+/year (95% CI) Percentage of days HSV+ (%)	43.86 (21.40, 89.91) 12.0	51.85 (31.07, 86.54) 14.2	48.09 (31.72, 72.90) 13.2	46.85 (25.50, 86.08) 12.8
Post booster Number of days HSV+/year (95% CI) Percentage of days HSV+ (%)	26.36 (12.04, 57.72) 7.2	37.14 (22.14, 62.31) 10.2	32.94 (21.18, 51.22) 9.0	34.21 (15.48, 75.59) 9.4

Note: Calculated using Poison regression and extrapolation to one year.

Subjects kept a diary, in which they self-reported outbreaks. We observed a comparable reduction in the mean number of outbreaks in each cohort receiving COR-1 or placebo (Table 1).

**Fig 6:**
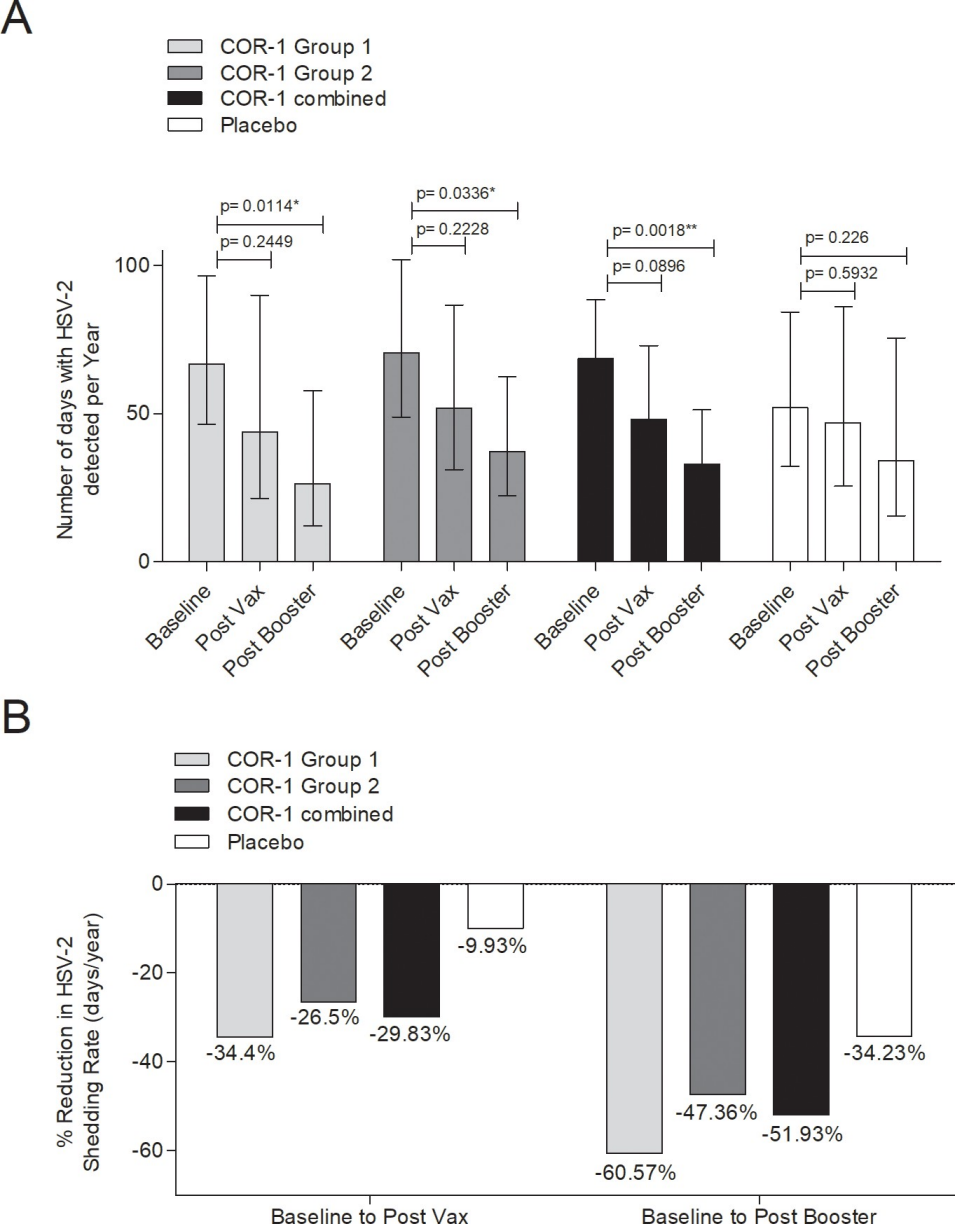
HSV-2 shedding before and after immunisation. HSV-2 shedding rates were assessed over 45-day swabbing periods before immunisation, after immunisation and after booster immunisation. **(A)** The number of days with HSV-2 detected was normalized per year. Shown are mean values with 95% CI. Significance was calculated using Poisson Regression analysis. *P<0.05, **P<0.01. **(B)** Percentage of reduction in HSV-2 shedding rate was calculated from mean number of days with HSV-2 detected per year.

The number of days with HSV-2 detected were normalized to per year for analysis. After three immunisations we observed non-significant reduction in the number of days with HSV-2 detected in cohorts receiving COR-1 (Group 1, Group 2 and combined), and the pooled placebo cohort (Fig 6A). After the booster immunisation the number of days with HSV-2 detected was further non-significantly reduced in the pooled placebo cohort, and significantly decreased in groups receiving COR-1 (Group 1, Group 2 and combined). The difference in the average number of days with HSV-2 detected after booster immunization was non-significant between groups receiving COR-1 or placebo. The significant reduction in viral shedding within each group receiving COR-1 resulted from a higher value of HSV-2+ days at baseline (Table 2). On average, subjects receiving COR-1 reached ~50% reduction in viral shedding after the booster immunisation compared to ~35% for placebo recipients, although the confidence intervals for both groups are wide (Fig 6B). Interestingly, Group 1 subjects receiving COR-1 reached >60% reduction in viral shedding after the booster immunisation, and Group 2 subjects reached 47%. The result might suggest that immunisation to two different sites (e.g. each arm) targeting different lymph nodes can be beneficial to vaccine induced immune responses. Of importance, the number of subjects in this study was insufficient to allow conclusive conclusions. Cautions should be taken to interpret the results in light of the small sample size. Although, statistical calculations are presented here, the lack of significance in the placebo group potentially reflects the low sample number, and we cannot exclude that a significant placebo effect is present.

## Discussion

Here we describe a phase I/IIa study evaluating the safety and tolerability of COR-1, a DNA-based immunotherapy to genital herpes, composed of plasmids encoding codon-optimized full length and ubiquitinated truncated gD2, in HSV-2 positive healthy subjects. As in the previous study [8], we found that a dose of 1 mg of COR-1 was safe and well tolerated with no moderate or serious adverse events. Further, this was a double-blinded placebo-controlled and randomized study to explore immunogenicity and therapeutic potential of COR-1 in HSV-2 seropositive subjects, and compared two groups which received the vaccine dose to either one or both forearms based on the hypothesis that targeting multiple immunization sites will increase vaccine efficacy. Of note, the study was not powered to rigorously assess immunogenicity and clinical efficacy due to the low number of subjects. Also, subjects of groups 2 were recruited after group 1, possibly contributing to temporal effects. Hence, immunogenicity and clinical efficacy endpoint evaluations were secondary or exploratory. The rate of patient reported recurrence of clinical signs and symptoms was reduced after immunisation with both COR-1 or placebo. As self-reported outbreaks are by nature subjective and more prone to placebo effects (subjects are not always consistent in reporting outbreaks over a pro-longed study period), viral shedding is regarded as a more accurate endpoint measurement than the traditional recurrence rate [4] and has been recommended as a surrogate outcome for herpes recurrences [10]. After immunisation with COR-1 we observed trends in reduction of viral shedding, and trends in induction of antigen-specific cellular and humoral immune responses in peripheral blood. However, a larger study cohort is required to test if COR-1 drives significant immunogenicity and clinical efficacy.

Subjects were deemed eligible to participate in the study if viral shedding could be detected on at least one day within the 45-day swabbing period during screening. As viral shedding appears to occur periodically [11], the study design was expected to result in some degree of reduction in viral shedding from baseline to post immunisation. This may account for the reduction in viral shedding observed in subjects receiving placebo. However, more than 60% reduction was observed in subjects receiving COR-1 administered to two arms, suggesting that COR-1 may have therapeutic potential in patients suffering from genital herpes. Of note, the viral shedding rate of both groups receiving COR-1 was higher during baseline swabbing compared to placebo groups (for unknown reasons). We observed a shedding rate of ~19% (percentage of days with HSV-2 detected) in subjects receiving COR-1 at baseline and ~14% in subjects receiving placebo, which is a comparable range to a recently published vaccination study which assessed baseline viral shedding in HSV-2+ subjects [12]. Also, this study experienced a high rate of early withdrawals resulting in only 10 of 17 COR-1 group 1 subjects and 9 of 10 placebo subjects completing swabbing post vaccination and booster as per protocol. Hence, the number of subjects completing the study was too low to allow formal conclusions regarding therapeutic efficacy of the vaccine.

In contrast to our previous study, where COR-1 did not induce any gD2-specific antibodies in HSV negative subjects [8], we detected a small but detectable rise in gD2-specific antibodies in HSV-2 infected subjects in response to COR-1. Note that the recombinant gD2 protein used for antibody detection was only partial length, hence antibody titer changes to other parts of gD2 could have been missed, but peptides used to detect T cell responses covered the full length of gD2. In HSV-2 negative healthy subjects, COR-1 induced gD2 antigen-specific cell-mediated immunity in 19 out of 20 subjects [8]. Here, we assessed if COR-1 could enhance gD2 antigen-specific cell mediated immunity in subjects with pre-existing responses. We considered a minimum of 2-fold increase to any gD2 peptide pool from baseline as a vaccine-related response and found that 60% of subjects were responders to the initial doses of immunisation when delivered to two arms, but the booster immunisation did not further increase cell-mediated immunity. A comparison to our previous study in HSV-2 seronegative subjects is not possible as T cell responses were measured by different protocols and institutions and using different peptide pools.

Interestingly, while booster immunization did not further increase the gD2-specific cell-mediated immunity, further reduction in viral shedding continued. A possible explanation for this is that blood is not the right correlate to measure but the anatomic locus of effector cells is probably ganglia or skin, and maybe it requires a booster immunization to facilitate this migration.

One challenge in the development of therapeutic vaccines to persistent viral infections is overcoming potentially exhausted and anergic virus-specific T cell responses due to high loads of chronic antigen exposure [13]. Flechtner and colleagues have also observed that antigen-specific effector T cell responses were not further increased after the initial dose of immunization with a bivalent adjuvanted protein HSV-2 immunotherapeutic vaccine candidate [14, 15]. Interestingly, in the present study, while the gD2-specific T cell response was not further increased with booster immunisation, viral shedding was further reduced, suggesting that the systemically measured antigen-specific IFNγ^+^ T cell response in blood might not be a decisive associate of viral shedding. We conclude that COR-1 is immunogenic in HSV-2 positive subjects, but optimization is necessary to improve efficacy, which could be achieved by escalating the dose.

Optimization of an HSV-specific immunotherapy could be addressed by enhancing antigen-specific adaptive cellular immunity, or by directing antigen-specific effector memory T cells more effectively to the site of disease [11], in either case to reduce viral shedding, moderate the length and severity of outbreaks, and thereby reduce transmission. An interesting observation of this study was that subjects receiving COR-1 to two arms displayed a non-significant trend of higher cell-mediated immune responses (magnitude and prevalence) and reduction in viral shedding compared to subjects receiving the same dose of COR-1 to one arm. This suggests that multiple site immunisation may be more immunogenic than single site, perhaps by targeting more lymph nodes. CD8^+^ tissue-resident memory T cells remain in the skin after encountering HSV [16], potentially serving as sentinels for and effectors against secondary infection. Hence, a strategy to increase the concentration of antigen-specific CD8^+^ memory T cells at the site of recurrences/ infection might benefit both therapeutic outcomes in HSV-2 positive subjects and prophylactic vaccine strategies respectively. Antibody responses are generally believed to prevent primary infections, while T cell mediated responses combat persistent infections. Seeding HSV-specific memory T cells in the genital tract could potentially assist with reducing the intensity of primary infection and thus seeding of the ganglia [17]. Immunisation in close proximity to the genital area or implementing a prime and pull strategy might therefore enhance vaccine effectiveness. A prime and pull immunisation strategy, in which chemokines were applied topically to the genital site in mice that previously received immunisation, protected against HSV infection of neurons [17]. Genital intradermal immunization with a human papillomavirus plasmid vaccine to cervical and vulvar intraepithelial neoplasia lesions resulted in positive patient outcomes [18, 19]. Hence, different delivery strategies to enhance the clinical outcome of COR-1 immunisation are worth exploring.

Other recent trials for therapeutic HSV-2 vaccines tested vaccine candidates from Genocea, Vical, Agenus and Sanofi [6]. Genocea tested a protein subunit vaccine GEN-003 composed of truncated gD2 and ICP4 in combination with a novel adjuvant Matrix-M2, which is a saponin-based lipid particle. GEN-003 induced significant reduction in viral shedding and lesion rates, and induced humoral and cell-mediated antigen-specific immune responses [14, 15, 20]. However, Genocea decided to not progress into costly and high-risk Phase III trials at this stage. Vical tested a plasmid vaccine VCL-HB01 consisting of polynucleotides encoding codon-optimized gD2 and VP11/12 in combination with Vaxfectin, a lipid-based compound designed to enhance protein expression. In a phase I/IIa trial they observed significant reductions in lesion rates and viral loads after immunisation, but placebo induced a comparable reduction in lesion rates and an even higher reduction in viral shedding (http://www.vical.com/investors/news-releases/News-Release-Details/2015/Vical-Reports-Top-Line-Results-From-Phase-12-Trial-of-Therapeutic-Genital-Herpes-Vaccine/default.aspx). Motivated by positive results collected at 9 months after immunisation [21], Vical conducted a phase II trial with results yet to be reported (NCT02837575). Agenus tested a peptide vaccine HerpV in combination with QS-21 adjuvant in a Phase II trial in HSV-2 positive subjects (NCT01687595), which resulted in some degree of reduction in viral shedding [4]. Another trial in collaboration with Sanofi is currently evaluating safety, tolerability and immunogenicity of a live, replication-deficient vaccine HSV529 [22] in a phase I study in HSV-2 positive subjects (NCT02571166). Primary endpoints of this study include the analysis of T cell density and TCR composition in genital lesion and non-lesion skin biopsies after immunisation, indicating that this study’s focus lies on tissue-resident memory T cell induction at the site of recurrence as discussed above.

Developing prophylactic and therapeutic vaccines against chronic viral infections, such as HSV, HPV, HIV, HBV, HCV and CMV, is challenging compared to vaccines against viruses that are naturally cleared from the host [23]. Viruses causing persistent infections often develop mechanisms to evade the host immune response. HSV for example has evolved mechanisms to suppress interferon responses by down-regulating pattern recognition receptors or blocking the localization of interferon response factors to the nucleus. Besides these mechanisms to disturb innate immune signaling, HSV has also evolved mechanisms to escape adaptive immunity through counteracting antigen presentation and thereby evade HSV-specific CD8+ T cell mediated lysis (reviewed in [24]). Though viral clearance of HSV-2 might be hindered by these mechanisms, reducing viral shedding and viral load in the genital mucosa would have a positive individual impact by reducing painful outbreaks. Further, it has been suggested that there is a viral load threshold associated with transmission [25]. With this in mind, a therapeutic HSV-2 vaccine reducing the viral load could not only decrease the global burden of HSV-2 but also HIV as HSV-2 infection is associated with higher prevalence of HIV infection [26].

## Supporting information

S1 CONSORT Checklist (DOC)

S1 File Clinical study protocol. (PDF)

S1 Fig Study schedule of events. (TIF)

S1 Table HSV-2 history. (DOCX)

S2 Table Demographics. (DOCX)

S3 Table Study schedule of events. (DOCX)

S4 Table Summary of Treatment-Emergent Adverse Events occurring in ≥1 subject by system organ class (Safety Population). (DOCX)

S5 Table Swabs collected for early withdrawals. (DOCX)
