# Supplementary material for: Immune responses to a HSV-2 polynucleotide immunotherapy COR-1 in HSV-2 positive subjects: A randomized double blinded phase I/IIa trial
Source: PLoS One. 2019 Dec 17;14(12):e0226320. doi: 10.1371/journal.pone.0226320 (PMC6917347; doi:10.1371/journal.pone.0226320)
Supplement: S1 File — (PDF) [file pone.0226320.s002.pdf]

Clinical Study Protocol

**Study Title:**	A Phase I/IIa, randomized, double blind, placebo-controlled, parallel group, pilot study to assess the safety and efficacy of a therapeutic HSV-2 DNA vaccine in HSV-2 positive adults
**Investigational Product:**	COR-1
**Indication:**	Genital Herpes
**Name and Address of Sponsor:**	Admedus Vaccines Pty Ltd<br>Registered Office:<br>Level 7<br>General Purpose South Building<br>Staff House Road,<br>The University of Queensland<br>St. Lucia Campus,<br>Brisbane, Queensland, 4072 AUSTRALIA
**Protocol Number:**	ADM-COR-1-2001
**Development Phase:**	Phase I/IIa
**Investigator(s):**	Dr Paul Griffin
**Version No:**	FINAL, Protocol Version 4-0
**Date of Version:**	11 Mar 2016

ADMEDUS VACCINES PTY LTD

Registered office: Level 7
General Purpose South Building, Staff House Road,
The University of Queensland,
St. Lucia Campus, Brisbane, Queensland, 4072, Australia

STUDY ACKNOWLEDGEMENT

Title: A PHASE I/IIA, RANDOMIZED, DOUBLE BLIND, PLACEBO-CONTROLLED, PARALLEL GROUP, PILOT STUDY TO ASSESS THE SAFETY AND EFFICACY OF A THERAPEUTIC HSV-2 DNA VACCINE IN HSV-2 POSITIVE ADULTS.

Protocol Number: ADM-COR-1-2001
Version: 4-0, Dated: 11 March 2016

This protocol has been approved by Sponsor. The following signature documents this approval:

______________________	______________________
Name (Printed)	Signature
	______________________
	Date (dd mmm yyyy)

INVESTIGATOR STATEMENT

I have read the protocol, including all appendices, and I agree that it contains all necessary details to conduct this study as described. I will conduct this study as outlined herein and will make a reasonable effort to complete the study within the time designated.

I will provide all study personnel under my supervision copies of the protocol and access to all information provided by Admedus Vaccines Pty Ltd. I will discuss this material with them to ensure that they are fully informed about the drugs and the study.

______________________	______________________
Investigator's Name (Printed)	Signature
______________________	______________________
Site number	Date (dd mmm yyyy)

1 STUDY SYNOPSIS

**Protocol No.:**	ADM-COR-1-2001
**Study Title:**	A Phase I/IIa, randomized, double blind, placebo-controlled, parallel group, pilot study to assess the safety and efficacy of a therapeutic HSV-2 DNA vaccine in HSV-2 positive adults
**Investigational Product:**	COR-1
**Indication:**	Genital Herpes
**Development Phase:**	Phase I/IIa
**Primary objectives:**	To evaluate:<br>• The safety and tolerability of two injection regimens of the HSV-2 DNA vaccine COR-1 compared with placebo, administered by intradermal injection as three, 4-weekly doses followed by a 6-month booster to otherwise healthy symptomatic HSV-2 positive subjects;
**Secondary objectives:**	To investigate the impact of COR-1 on:<br>• Induction of an antigen specific humoral and/or cell mediated immune response against gD2.
**Exploratory objectives:**	To evaluate:<br>• The effect of COR-1 on HSV-2 shedding;<br>• The incidence of symptomatic HSV-2 genital recurrence;<br>• The nature of the immunological responses to COR-1, including local tissue responses.
**Primary endpoints:**	Safety:<br>• Comparative incidence and severity of adverse events in each treatment group, including vaccine related adverse events:<br>o Incidence and severity of local reactions (soreness, redness, induration, ecchymosis, oedema, itching and paraesthesia) at the site of vaccination;<br>o Incidence and severity of systemic reactions (fatigue, myalgia, malaise, fever, rigors, arthralgia, nausea, diarrhoea, light headedness, dizziness, hypersensitivity and headache).
**Secondary endpoints:**	Immunogenicity:<br>• Change from Baseline in T-cell and/or antibody responses to HSV-2 gD2 as measured by:<br>o ELISA (antibody);<br>o Interferon-gamma (IFNγ) enzyme linked immunospot (ELIspot) assays (T-cell responses).
**Exploratory endpoints:**	Efficacy:<br>• Number of days with detectable viral shedding after 3-dose vaccination, compared with Baseline;<br>• Number of days with detectable viral shedding after the booster injection compared with Baseline;<br>• Symptomatic genital herpes recurrence rate;<br>• Time to first recurrence of symptomatic genital herpes

	measured from first administration of study vaccine;<br>• Number of symptomatic recurrences of genital herpes during the course of the study (to Week 48);<br>• Viral load level after three dose levels compared to baseline;<br>• Viral load level after booster compared to baseline.<br><br>Immunogenicity<br>• Change from Baseline in T-cell and/or antibody response as measured using other exploratory measures of immune function. Such measures of humoral and T-cell responses may include (but are not limited to):<br>o T-cell responses (IFNγ, interleukin 2 [IL-2], tissue necrosis factor alpha [TNFα] and other cytokine production by CD4 positive (+) or CD8+ T-cells as measured by intracellular cytokine staining (ICS);<br>o Where responses to a whole pool of peptides are obtained, positive responses may be assessed with a matrix of peptides to give information on the breadth of the response and likely epitopes governing the responses;<br>o Innate immune responses (e.g. number and function of dendritic cells);<br>o Proportion and phenotype of T-cells measured by flow cytometry and MHC tetramer analysis;<br>o Influence of HLA type on monitored immune response;<br>o Change from baseline of T-cell receptor type;<br>• Histological assessment of the nature of local tissue response at the site of vaccination.
**Study design:**	Randomized, single or multi centre, double blind, placebo-controlled, parallel group, pilot study.<br><br>There are two primary reasons for inclusion of the 2 placebo arms:<br>• To maintain the blind; and<br>• To describe any natural variation in viral shedding rates between the baseline shedding assessment pre- and post-administration of the placebo.
**Number of participants:**	40
**Number of centres:**	One or more clinical trial centres
**Inclusion criteria:**	1. Diagnosis of genital HSV-2 infection confirmed by positive HSV-2 serology or if negative a documented positive HSV-2 pathology result, AND positive PCR for HSV-2 during the Baseline Shedding Assessment prior to randomization.<br>2. A history of recurrent genital HSV-2 for at least 12 months AND a self-reported history of at least 3 and no more than 9 reported lesion occurrences in the 12 months prior to Screening or, if currently on suppressive therapy, in the 12 months prior to initiation of suppressive therapy.<br>3. Aged 18 to 50 years, inclusive.<br>4. Male or female. Women of child-bearing potential must be using two effective methods of contraception and agree to continue to do so from Screening, throughout Investigational vaccine/placebo dosing and for 28 days after the last dose of

	investigational vaccine/placebo.<br>5. Has voluntarily given written informed consent.
**Exclusion criteria:**	1. Birthmarks, tattoos, wounds or other skin conditions on the forearms which could reasonably obscure injection site reactions.<br>2. Inadequate venous access to allow collection of blood samples.<br>3. Female subjects who are breastfeeding and those of reproductive potential with a positive urine beta human chorionic gonadotropin (β-HCG) pregnancy test at either Screening or the Baseline Visit (Visit 5).<br>4. Received immunomodulating agents (including immunosuppressive agents, interferon or other immune or cytokine-based therapies), and/or systemic chemotherapeutic agents within 60 days of Screening or expected to receive these agents during the course of the study. Stable use asthma inhalers and topical corticosteroids are permitted. All medications will be documented and reviewed for acceptance by the Investigator or a medically qualified nominee.<br>5. Receiving medication known to have anti-HSV-2 activity within 7 days of the Baseline shedding assessment.<br>6. History of or currently active serious medical or psychiatric illness which, in the opinion of the Investigator, may impair the ability to provide written informed consent and would interfere with treatment, assessment, compliance with the protocol, or subject safety.<br>7. History of HSV-1 genital infection, ocular HSV infection, HSV-related erythema multiforme, herpes meningitis or encephalitis.<br>8. HSV-1 positive PCR result during the Baseline Shedding Assessment.<br>9. Laboratory blood values:<br>a. Haemoglobin <12.0 grams/decilitre (g/dL) for men and <11.0 g/dL for women<br>b. Neutrophil count <1000/mm3<br>c. Platelet count <80,000/mm3<br>d. Aspartate aminotransferase (AST) or alanine aminotransferase (ALT) >1.5 times the upper limit of normal (ULN)<br>e. Amylase >1.5 times ULN (unless serum lipase is ≤1.5 times ULN)<br>f. Subjects with an estimated creatinine clearance of <80 mL/minute (min)<br>g. INR > ULN<br>h. Hepatitis B surface antigen (HBsAg), Hepatitis C Virus (HCV) antibody or HIV antibody positive.<br>10. Subject has received any vaccine or investigational drug within 30 days prior to Visit 2, or is due to receive vaccines

	during the treatment period.<br>11. Subject has participated in the past in another clinical trial of a vaccination related to infection with HSV (unless it can be verified that the subject received placebo).<br>12. Any known or suspected allergies to the study drug or its constituents.<br>13. Subject has a compliance of less than 80% of swab sample collection during the Baseline shedding assessment i.e. immediately prior to randomisation.<br>14. Previous participation in this study or the previous COR-1 Phase I study.<br>15. Subject unwilling to abstain from blood donation during the course of the study.
**Study product:**	COR-1 is a DNA vaccine<br>The investigational product is a 1:1 mixture of two DNA plasmids:<br>1. COR-1A: plasmid DNA NTC8485-O2-gD2<br>2. COR-1B: plasmid DNA NTC8485-O2-UgD2tr<br>COR-1 is formulated in sterile, isotonic and endotoxin free Tris (hydroxymethyl) amino methane hydrochloric acid and Ethylenediaminetetraacetic acid (EDTA) at pH 8 (TE buffer).<br>Placebo vaccine is sterile, isotonic and endotoxin free TE buffer.
**Control Group:**	Placebo to match.
**Dosage and regimen**	All subjects will receive either 2 intradermal injections of 200µL, to the forearm(s).<br><br>Subjects will be randomised to receive either investigational vaccine or placebo.<br><br>There will be 2 subject groups. Once Group 1 is fully allocated, Group 2 will then be opened for recruitment.<br><br>Group 1 patients will receive one injection into each forearm (i.e. two injections in total at each vaccination) of either:<br>• Group A: 15 subjects will receive 2 x 500mcg of COR-1<br>• Group B: 5 subjects will receive 2 x placebo.<br><br>Group 2 patients will receive two injections into the one forearm (i.e. two injections in total at each vaccination) of either:<br>• Group C: 15 subjects will receive 2 x 500mcg of COR-1<br>• Group D: 5 subjects will receive 2 x placebo.
**Study procedures:**	Please refer to Table 1.<br><br><u>Screening/</u> <u>Run in:</u><br><br>Subjects who provide voluntary written informed consent will be screened for preliminary eligibility. At screening, subjects will also be invited to provide written informed consent for the optional skin biopsy that will be collected at Visit 10.<br><br>Preliminarily eligible subjects will commence collecting once daily swabs of genital secretions for HSV-2 testing for 45 days from Visit 2. During this and each subsequent swabbing period,

	subjects will be given diaries and asked to collect daily information about swab collection and HSV-2 symptoms that they may experience.<br><br>Two days after the commencement of the Baseline shedding assessment period, subjects will be contacted via telephone by the Investigator or designee to discuss the subject's progress and compliance with daily swab collection. If there are any doubts about the quality of the swabbing or compliance, the subject will be encouraged to attend an unscheduled clinic visit so that further education can be provided. A week after the patient commenced the Baseline Shedding Assessment (Visit 4) the site may contact the subject via telephone again to check compliance and address any logistical issues with shipment of swabs to the designated virology laboratory. Visit 4 is optional and dependant on the subject's requirements, as assessed by the Investigator or designee.<br><br>The site will be encouraged to keep regular phone contact with the subject during the Run-in shedding assessment to ensure that the subject can comply with this requirement. Unscheduled clinic visits may also be arranged for this purpose.<br><br>Should a subject develop genital lesions during the Baseline shedding assessment they will be instructed to contact the clinic. An additional set of swabs will be collected for PCR or viral culture.<br><br>Study Period 1 (Treatment Period):<br><br>At Baseline (Day 0), subject eligibility will be confirmed. Subjects meeting all inclusion and no exclusion criteria will be randomised to receive either the investigational vaccine or placebo will be in a 3:1 ratio. In total, the subjects will receive 3 doses of investigational vaccine or placebo at 4 weekly intervals (Day 0, Week 4, and Week 8). The subject will keep a diary and record any injection site reactions each day for 7 days after receiving the injection(s). Subjects will return to the clinic one week after each administration of investigational vaccine/placebo for immune monitoring at Week 1, 5 and 9 (Visits 6, 8 and 11).<br><br>In addition, subjects will return to the clinic 48 hours (Visit 10) after their third dose of investigational vaccine/placebo received at Week 8. The Investigator will assess the subject's injection site(s). The Investigator or designee will take a photo using a standardized approach. Additionally, all subjects who provide written informed consent for the skin biopsy at screening will have the tissue collected from the injection site region regardless of whether they have had a visible injection site reaction. Please see Section 6.4.11 of the protocol.<br><br>One week after the third vaccination (Week 9), subjects will commence collecting post-treatment swabs for viral culture for 45 days. Subjects will return at Week 12 (Visit 12) for immune monitoring and safety assessments and at Week 15 (Visit 13) having completed the 45 days swab collection.<br><br>At Week 19, subjects will be contacted by phone and adverse

	event/concurrent medication questioning will occur<br><br><u>Study Period 2 (Booster Injection):</u><br>At Week 23 (Visit 15), one week prior to the booster vaccination/placebo administration, the subject will attend the clinic and safety assessments will be performed.<br>At Week 24 (Visit 16), subjects will be administered a single booster vaccination/placebo.<br>At Week 25 (Visit 17), immune monitoring will be performed. Subjects will also be required to collect genital swabs for viral culture for 45 days from Week 25. Swabs will be sent to the laboratory for viral load testing, virus typing and/or sequencing.<br>At Week 28 (Visit 18), immune monitoring and safety assessments will again be performed.<br><u>Follow up:</u><br>Subjects will be contacted via the telephone at Week 36 and 42 (Visits 20 and 21). Adverse event and concurrent medications will be recorded.<br>End of Study:<br>At Week 48 (Visit 22), safety assessments will be performed.<br><br>Additional information:<br>Subjects will be required to suspend all HSV-2 medication during the shedding assessments and for one week prior to the Run-in shedding assessment and throughout the study period.<br>Urine pregnancy testing will be conducted for female subjects at Screening, before administration of each dose of investigational vaccine/placebo and at the End of Study Visit. If positive, a serum pregnancy test will be conducted and the study investigational vaccine/placebo will not be administered until the result of this is received. If the result of the serum pregnancy test is negative, administration of investigational vaccine /placebo will continue. If the result of the pregnancy test is positive, the subject will be withdrawn from the study.<br>Vital signs will be recorded prior to study vaccine/placebo administration and at 30 minutes and 60 minutes following each dose. Subjects will be required to remain in the clinic for at least 60 minutes after administration or longer if clinically indicated.<br>Blood samples will be collected and shipped to nominated laboratories for preparation of serum and peripheral blood mononuclear cells (PBMCs) for use in immunological analyses (T-cell and antibody responses).
**Contraindications to further dosing:**	Subjects will be withdrawn from treatment permanently should any of the following occur:<br>• Treatment related Grade 3 or 4 toxicity<br>• The need to take medication which could interfere with study

	measurements<br>• Subject unwilling to proceed and/or consent is withdrawn. Where possible subjects who are withdrawn from treatment will remain on the study and attend the scheduled study visits, provided the Investigator deems it appropriate and the Sponsor approves.
**Safety parameters:**	Refer to Tables 1 & 2
**Clinical procedures / assessments:**	Refer to Tables 1 & 2
**Specialised analyses:**	• T-cell responses to HSV-2 gD2 will be primarily assessed by interferon gamma Enzyme-Linked Immunosorbent Spot assay (ELIspot) at specified time points during the study period.<br>• Antibody responses against HSV-2 gD2 will be monitored using ELISA.<br>• PCR analysis for presence and quantification of viral shedding and type.<br>• Other exploratory immunological assays may be performed such as the monitoring of both T-cell responses (e.g.using intracellular cytokine staining, FACS and MHC tetramer analysis), T-cell receptor and HLA typing and humoral immune responses against HSV-2 antigens(e.g. using ELISA based methodologies).
**Sample size determination:**	The samples size for this pilot study has been determined based on practical and logistical constraints. The study is not designed to be powered for hypothesis testing or precision with regard to parameter estimation.<br>.
**Statistical analyses:**	An interim analysis of unblinded immunogenicity and efficacy data is planned In summary<br>• Immunogenicity data (ELISPOT and ELISA); and<br>• Viral shedding data<br>Since the study is a pilot without consideration given to formal hypothesis testing and statistical power, the focus of the statistical analysis will be descriptive. As such, summary descriptive statistics relevant for the various endpoints will be provided for each of the individual treatment groups at the various study time points. In addition, depending on the similarity of the 2 placebo groups, the placebo groups may also be pooled. If inferential statistical tests or models are employed, they will be viewed as exploratory and descriptive in nature.<br>Detailed aspects of the analyses will be described in the Statistical Analysis Plan prior to breaking of the study blind for both the interim and final analyses.
**Committees / guidelines:**	A Safety Review Committee will convene at regular intervals to review progress of the study including any safety concerns. Please refer to Section 9 of the protocol.
**Special protocol requirement / issues:**	Subjects will be required to collect daily swabs from

	mucocutaneous genital sites for viral shedding for 45 days at various time points during the study.<br><br>If the subject develops lesions at any time of the study, they will be instructed to swab the affected area as soon as possible and to report to the clinic within 24 hours (unless this occurs during the weekend. In this instance, as soon as possible). The clinic will conduct a clinical examination and collect swabs to confirm the diagnosis. The subject will continue to collect a swab from the affected area for viral culture each day until the lesion heals.<br><br>Subjects will be requested to complete a diary during the shedding assessments and for each week following the administration of the investigational vaccine/placebo. There are two diaries that will be used to capture this information including:<br>• Injection Site Reaction Diary, and<br>• HSV-2 Symptoms and Swabbing Diary.<br><br>Safety assessments will be performed immediately prior to each injection(s). If the subject experiences any of the following, their injection(s) will be delayed for up to one week:<br>• Temperature of > 38 degrees Celsius;<br>• Acute intercurrent systemic illness.<br><br>If the elevated temperature or acute intercurrent systemic illness does not resolve within 7 days, the Investigator or designee will seek advice from the Medical Monitor.

Figure 1. Study Plan Overview

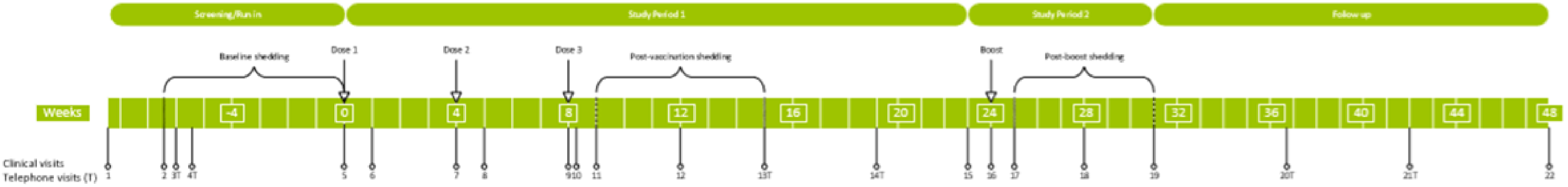

Table 1. Table of Assessments 1 – Screening/Run-in to Study Period 1

Assessments	Screening/Run in [8]				Study Period 1[8]									
	Visit 1	Visit 2	Visit 3	Visit 4[13]	Visit 5[15]	Visit 6	Visit 7	Visit 8	Visit 9	Visit 10[11]	Visit 11	Visit 12	Visit 13	Visit 14
	Day -46 to -73	Day -45	Day -43	Day -38	Baseline D0	D7	D28	D35	W8	W8 + 48 hours	W9	W12	W15	W19
Informed consent	X													
Review inclusion/exclusion criteria	X				X									
Demographics, Medical/Surgical History	X													
Physical Examination (symptom directed)	X				(X)	(X)	(X)	(X)	(X)	(X)	(X)	(X)	(X)	
Vital signs/Body Weight/Height [1, 2, 3, 4]	X				X		X		X			X		
Electrocardiogram[3]	X													
Urine Pregnancy Test[5]	X				X		X		X					
Haematology [3]	X				X	X	X	X	X		X	X		
Chemistry[3]	X				X	X	X	X	X		X	X		
Coagulation	X													
Urinalysis[3]	X				X		X		X					
Viral Serology (HIV, Hepatitis B and C, HSV-1 and 2)	X													
ELISA (antibody)					X		X		X			X		
ELIspot (T cell)					X	X		X			X			
Exploratory Immune assays (T cell and/or genetic testing)[14]					X			X			X			
Randomisation					X									
Phone call			X	X										X
Study vaccine administration and bleb check/measurement					X		X		X					
Collection of daily swabs from mucocutaneous genital sites for viral shedding for 45 days		X									X			
Take photos of injection site reactions[12]						(X)		(X)		X	(X)			
Issue and collect Diary[7]		X			X	X	X	X	X		X		X	
Review diary cards for compliance and adverse events.			X	X	X	X		X		X	X	X	X	
Optional skin biopsy										X				
Patient education regarding swab collection, diary completion and outbreak management[6]		X	X[10]	X[10]							X	X		
Adverse Event and Outbreak assessment		X	X	X	X	X	X	X	X	X	X	X	X	X
Concurrent Medications	X	X	X	X	X	X	X	X	X	X	X	X	X	X

Table 1 Table of Assessments 2 –Study Period 2 to Follow up

Assessments	Study Period 2[8]					Follow up[9]		
	Visit 15	Visit 16	Visit 17	Visit 18	Visit 19	Visit 20	Visit 21	Visit 22
	W23	W24	W25	W28	W31	W36	W42	End of Study/ W48
Informed consent								
Review inclusion/exclusion criteria								
Demographics, Medical/Surgical History								
Physical Examination (symptom directed)	(X)	(X)	(X)	(X)	(X)			(X)
Vital signs/Body Weight/Height [1, 2, 3, 4]		X		X				X
Electrocardiogram[3]								
Urine Pregnancy Test[5]		X						X
Haematology [3]	X	X	X	X				X
Chemistry[3]	X	X	X	X				X
Coagulation								
Urinalysis[3]		X						X
Viral Serology (HIV, Hepatitis B and C, HSV-1 and 2)								
ELISA (antibody)		X		X				X
ELIspot (T cell)		X	X					X
Exploratory Immune assays (T cell and/or genetic testing)[14]		X	X					X
Randomisation								
Phone call						X	X	
Study vaccine administration and bleb check/measurement		X						
Collection of daily swabs from mucocutaneous genital sites for viral shedding for 45 days			X					
Take photos of injection site (s)[12]			X					
Issue and collect Diary[7]		X	X		X			
Review diary cards for compliance and adverse events.			X	X	X			
Skin biopsy								
Patient education regarding swab collection, diary completion and outbreak management[6]			X	X				
Adverse Event and Outbreak assessment	X	X	X	X	X	X	X	X
Concurrent Medications	X	X	X	X	X	X	X	X

1	Height and weight to be performed at Visit 1 only
2	Vital signs include resting BP, heart rate, respiratory rate and temperature
3	Safety assessments to be performed prior to investigational vaccine/placebo.
4	Vital signs to be performed prior to investigational vaccine/placebo administration at 30 minutes and 60 minutes following each dose.
5	Urine pregnancy testing will be conducted in female participants at Screening, before each administration of study vaccine and at the end of study visit. If positive, a serum pregnancy will be conducted and the vaccine will not be administered.

6	To be repeated as deemed necessary by site staff
7	Subject to document daily whether any HSV-2 symptoms have occurred during the shedding assessments. Following receiving the investigational vaccine/placebo, the subject to document the existence and nature of any injection site reactions that occur for 7 days.
8	Visit window is +/- 3 days
9	Visit window is +/- 5 days
10	Subjects to be either contacted via phone or visit clinic to assess swabbing technique. If subject has had difficulties in collecting swabs since Visit 2, the subject is to be re-educated.
11	Visit 10 has a visit window of +/- 12 hours
12	Site to take a photo of any injection site(s)
13	Optional phone visit for subjects that require additional support with the Baseline Swabbing Assessment
14	Genetic exploratory testing will only be performed in subjects who provide written informed consent. Genetic exploratory analysis may include HLA typing and T-cell receptor typing.
15	Visit window is +/- 7 days

Table of Contents

1 STUDY SYNOPSIS 3

2 STUDY CONTACTS 21

3 INTRODUCTION 22
3.1 Disease and current treatment 22
3.2 COR-1 22
3.2.1 *Pharmacology* 23
3.2.1.1 *In vitro* Pharmacology 23
3.2.1.2 *In vivo* Pharmacology 23
3.2.2 Preclinical Safety 23
3.2.2.1 *In vitro* Safety 23
3.2.2.2 *In vivo* Safety 24
3.2.3 Metabolism and pharmacokinetics 24
3.2.4 Clinical trials of COR-1 24
3.3 Rationale for the Study and Dose Selection 25

4 OBJECTIVES AND ENDPOINTS 27
4.1 Objectives 27
4.2 Endpoints 27
4.3 Study Design 28
4.4 Dosing Regimens 28
4.5 Study Sites 29
4.6 Estimated Duration of the Study 29

5 SUBJECT POPULATION 30
5.1 Selection and Number of Subjects 30
5.2 Inclusion Criteria 30
5.3 Exclusion Criteria 30
5.4 Other Study Eligibility Criteria Considerations 31
5.4.1 *Contraception* 31
5.4.2 *Renal Function* 32
5.5 Subject Enrolment 32
5.5.1 *Rescreening* 32

6 SCHEDULE OF ASSESSMENTS AND PROCEDURES 33
6.1 Study Schedule of Evaluations 33
6.2 Visit Windows 33
6.3 Study Procedures / Assessment Periods 33
6.3.1 *Screening/Run in visits* 34
6.3.1.1 Visit 1/Screening 34
6.3.1.2 Visit 2 / Day - 45 34
6.3.1.3 Visit 3 / Day - 43 35
6.3.1.4 Visit 4 / Day -38 35
6.3.2 Study Period 1 (Treatment period) 35
6.3.2.1 Visit 5/Day 0/Baseline 35
6.3.2.2 Visit 6 (Week 1) and 8 (Week 5) 36
6.3.2.3 Visit 7 (Week 4) and 9 (Week 8) 36
6.3.2.4 Visit 10 / Week 8 + 48 hours 36
6.3.2.5 Visit 11 / Week 9 37
6.3.2.6 Visit 12 / Week 12 37
6.3.2.7 Visit 13 / Week 15 38
6.3.2.8 At Visit 14 / Week 19 38
6.3.3 Study Period 2 (Booster injection) 38
6.3.3.1 At Visit 15 / Week 23 38
6.3.3.2 At Visit 16 / Week 24 38
6.3.3.3 At Visit 17 / Week 25 39
6.3.3.4 At Visit 18 / Week 28 39

6.3.3.5 At Visit 19 / Week 31 ... 40
6.3.4 Follow up ... 40
6.3.4.1 Visit 20 / Week 36 and Visit 21 / Week 42 ... 40
6.3.4.2 Visit 22 / Week 48 / End of Study (or early withdrawal from treatment visit) ... 40
6.4 Details of Scheduled Assessments ... 40
6.4.1 Demographic Data, Medical/Surgical History, Medication History and Concurrent Medication ... 40
6.4.2 Physical examination ... 41
6.4.3 Vital signs ... 41
6.4.4 Electrocardiograms ... 41
6.4.5 Blood and urine samples for laboratory tests ... 42
6.4.6 Pregnancy tests ... 42
6.4.7 Intradermal injection of COR-1 or placebo ... 42
6.4.8 Photos of injection site (s) ... 43
6.4.9 Genital viral shedding assessments ... 43
6.4.10 Diary Booklets ... 44
6.4.11 Punch biopsies ... 46
6.4.12 Handling and processing of biological specimens ... 46
6.5 Randomisation process ... 46
6.6 Blinding ... 47
6.7 Method of Unblinding ... 47
6.7.1 Medical Emergency ... 47
6.7.2 End of Study ... 47
6.8 Formulation ... 48
6.8.1 Investigational products ... 48
6.8.2 Supply, packaging and labelling, storage and handling ... 48
6.8.3 Dosage and administration of test drugs ... 49
6.8.4 Dispensing and accountability ... 50

7 CONCURRENT MEDICATIONS AND TREATMENTS ... 51
7.1 Special Dietary Requirements ... 51
7.2 Concurrent Medications/Treatments Not Permitted ... 51
7.2.1 Prior to study entry ... 51
7.2.2 During the study dosing period ... 51

8 ADVERSE EVENTS AND TOXICITY MANAGEMENT ... 52
8.1 Safety Parameters ... 52
8.2 Adverse Events ... 52
8.2.1 Assessment of AEs ... 53
8.2.2 Adverse Event Reporting Period ... 54
8.3 Serious Adverse Events ... 54
8.3.1 Serious Adverse Event Definition ... 54
8.3.2 Clarification of Serious Adverse Events ... 54
8.3.3 Serious adverse event reporting requirements ... 55
8.3.3.1 All SAEs ... 55
8.3.3.2 Investigator reporting requirements for SAEs ... 55
8.4 Follow up of Serious and Non-serious Adverse Events ... 55
8.5 Clinical Laboratory Abnormalities and Other Abnormal Assessments as AEs or SAEs ... 56
8.6 Guidance for dose modification or discontinuation of treatment ... 56
8.7 Warnings and Precautions ... 57
8.8 Restrictions ... 57
8.9 Risks for Women of Childbearing Potential or during Pregnancy ... 57
8.10 Procedures to be Followed in the Event of Pregnancy ... 58
8.11 Procedures to be followed in the Event of Symptomatic Genital Herpes (Outbreak) ... 58

9 SAFETY REVIEW COMMITTEE ... 60

10 SUBJECT COMPLETION/WITHDRAWAL ... 61

10.1 Subject Completion 61
10.2 Criteria for Premature Withdrawal from Treatment or the Study 61
10.3 Withdrawal of subjects from Study Product 61
10.4 Withdrawal of Subjects from the Study 61
10.5 Premature termination of the study 62

11 STATISTICAL ANALYSIS 63
11.1 Sample Size Determination 63
11.1.1 Analysis Sets 63
11.1.2 Subject Disposition, Demographics and Baseline Characteristics 64
11.1.3 Safety Evaluations 64
11.1.4 Adverse Events 64
11.1.5 Vital Signs, Laboratory Parameters, and Immunogenicity 64
11.1.6 Exploratory Efficacy Evaluations 64
11.1.7 Interim Analysis 65

12 GENERAL STUDY ADMINISTRATION 66
12.1 Ethical Aspects 66
12.1.1 Local regulations/Declaration of Helsinki 66
12.1.2 Informed consent 66
12.1.3 Premature withdrawal 66
12.1.4 Institutional Review Boards or Ethics Committees 66
12.1.5 Conditions for modifying the protocol 66
12.1.6 Conditions for terminating the study 67
12.2 Study Documentation, CRFs and Record Keeping 67
12.2.1 Investigator’s files/Retention of documents 67
12.2.2 Background data 68
12.2.3 Audits and Inspections 68
12.2.4 Case Report Forms 68
12.3 Monitoring the Study 68
12.4 Confidentiality of Trial Documents and Subject Records 68
12.5 Publication of Data and Protection of Trade Secrets 69
12.6 Anticipated Subject Accrual and Duration of the Study 69

13 REFERENCES 70

14 APPENDICES 72
14.1 APPENDIX 1: Procedure for Intradermal Injections 72
Equipment and materials: 72
Procedure: 72
14.2 APPENDIX 2: Guidance for Industry, Toxicity Grading Scale for Healthy Adult and Adolescent Volunteers Enrolled in Preventive Vaccine Clinical Trials, September 2007 74
14.3 APPENDIX 3: Injection site biopsy sub study 84

LIST OF TABLES

GLOSSARY OF ABBREVIATIONS

AE	Adverse Event
ADR	Adverse Drug Reaction
ALP	Alkaline Phosphatase
ALT	Alanine Transaminase
AST	Aspartate Transaminase
Beta-HCG	Beta Human Chorionic Gonadotropin
BMI	Body Mass Index (weight in kg divided by height in m2)
BP	Blood Pressure
cGMP	Current Good Manufacturing Practice
CI	Confidence Interval
CMI	Cell-mediated Immunity
COA	Certificate of Analysis
CRF	Case Report Form
CPMP	Committee for Proprietary Medicinal Products
CRO	Clinical Research Organisation
CS	Clinically Significant
CSR	Clinical Study Report
CTN	Clinical Trial Notification
CV	Coefficient of Variation
d	Day
DBP	Diastolic Blood Pressure
DIFF	Differential White Blood Cell count
DNA	Deoxyribonucleic Acid
ELISA	Enzyme-linked immunosorbent assay
ELISPOT	Enzyme-linked immunosorbent spot assay
EOS	End of Study
FBC	Full Blood Count
FDA	Food and Drug Administration (US)
GCP	Good Clinical Practice
GFR	Glomerular Filtration Rate
GGT	Gamma Glutamyltransferase
GH	Genitial Herpes
GLP	Good Laboratory Practice
gD2	Envelope glycoprotein D of HSV serotype2
GMT	Geometric Mean Titre
Hb	Haemoglobin

Hct	Haematocrit
HIV	Human Immunodeficiency virus
Hep B	Hepatitis B
Hep C	Hepatitis C
HPC	High Positive Control
HR	Heart Rate
HREC	Human Research Ethics Committee
HSV	Herpes Simplex Virus
IB	Investigator’s Brochure
ICH	International Conference on Harmonisation of Technical Requirements for Registration of Pharmaceuticals for Human Use
Inj	Injection
IUD	Intrauterine device
ITT	Intention to Treat
LFT	Liver Function Test
LLPC	Lower Limit Positive Control
LOQ	Limit of Quantification
LPC	Low Positive Control
NCS	Not Clinically Significant
OECD	The Organisation for Economic Co-operation and Development
OTC	Over-the-counter
PBMC	Peripheral blood mononuclear cells
mcg	Microgram
MedDRA	Medical Dictionary for Regulatory Activities
MPC	Mid Positive Control
NA	Not applicable
ND	Not Done
NHMRC	National Health and Medical Research Council
NOAEL	No Observable Adverse Effect Limit
NR	Not Recorded
NRQ	Test Not Required
NS	Sample Not Obtained
NTC	Nature Technology Corporation
PICS	Pharmaceutical Inspection Co-operation Scheme
PC	Positive Control
PP	Per Protocol
PSCP	Plate-Specific Cut Point
PT	Preferred Term
SAE	Serious Adverse Event
TEAE	Treatment Emergent Adverse Event

TGA	Therapeutic Goods Administration
U&E	Urine and Electrolytes
ULQ	Upper Limit of Quantification
UNK	Unknown
US/USA	United States (of America)
Vacc	Vaccination
WHO	World Health Organisation
WMA	World Medical Association

2 STUDY CONTACTS

Please refer to the Study Reference Manual for full study contacts.

3 INTRODUCTION

3.1 Disease and current treatment

Herpes Simplex Virus (HSV) type 2 is the major infectious agent that causes genital herpes (GH). Epidemiological studies estimate the incidence of acute GH infection in Europe and the United States to be between 5 and 24 cases per 100 people per year (Malkin, 2004). GH is characterised by the formation of blistering lesions associated with significant pain and discomfort. After an acute episode, the virus may persist in the body as a latent infection in dorsal root ganglia (Johnston 2011). The virus may reactivate, causing migration of virus to the mucosal surface and an outbreak of GH associated with lesion formation, pain and itching. Viral shedding may result in transmission of infection to a sexual partner (Mertz 2008). GH may also be passed from an infected mother to her new-born child in utero, peri-partum and post-partum. Most transmission occurs at the time of birth (Kimberlin 2007). In severe cases HSV infection can result in encephalitis, meningitis and even death. Genital herpes has recently been found to be strongly linked with HIV transmission in Africa (Abu-Raddad et al., 2008).

To date, treatments have failed to eradicate HSV in an infected individual. Antiviral medications have been shown to reduce the frequency, duration, and severity of outbreaks. Available drugs include acyclovir, valaciclovir, famciclovir, penciclovir, and docosanol. However, these treatments have been unable to eliminate outbreaks or completely prevent shedding. Recurrence of active lesions and asymptomatic viral shedding may therefore result in continued transmission of disease.

DNA vaccines consist of bacterial plasmids, small double strands of non-chromosomal DNA, inserted with gene sequences that encode for specific surface proteins of the target infectious agent. The plasmids typically also contain a unique promoter sequence. Injection of the plasmid into the nucleus of host cells results in transcription and translation of the gene into copies of viral proteins. The foreign antigen complexes are subsequently presented at the host cell surface allowing the immune system to detect and recognize them. A range of immune responses result, including migration of the foreign antigen: immune cell complex to the lymph node.

Previous clinical trials of HSV DNA vaccines have failed to produce a significant or complete reduction of outbreaks or viral shedding, despite induction of high antibody titres (Corey et.al 1999). It is believed that an antibody response needs to also be accompanied by a strong cell mediated immune response.

It is increasingly believed that the ability to potentiate a HSV-2 specific cell mediated immune response will be a critical component of any successful therapeutic HSV-2 vaccine. Such a treatment would result in reduced recurrence, transmission and potential life- threatening herpes infections.

3.2 COR-1

This is a brief summary of the preclinical and clinical profile of COR-1. More detailed information is available in the Investigator’s Brochure.

Admedus’ HSV vaccine (COR-1) is a 1:1 mixture of two DNA plasmids formulated in sterile, isotonic and endotoxin-free TE buffer. Both plasmids comprise the Nature Technology Corporation (NTC) expression vector NTC8485 minus the enhanced green fluorescent protein sequence. The first has a codon optimised DNA sequence insert encoding full length glycoprotein D from HSV-2 (gD2) (named NTC8485-O2-gD2) and the other with a codon optimised DNA sequence insert encoding one ubiquitin repeat fused to the N-terminal end of amino acids 25-331 of truncated gD2 (named NTC8485-O2-Ubi- gD2tr).

COR-1 differs from other vaccines in development for the treatment of HSV-2 as it is designed specifically to generate both humoral (antibody) and cellular (T-cell) immune responses to the primary surface glycoprotein antigen gD2 of HSV-2. This immune response is expected to better control and protect against HSV-2 infection than either humoral or cellular response alone.

There have been previous concerns regarding the use of DNA vaccines including the possibility of integration into the host genome potentially resulting in tumors. There is however no evidence of integration of plasmid DNA into the host genome making the development of tumor's unlikely (Manam et al., 2000).

Concerns also have arisen regarding the potential to transfer of antibiotic resistance. COR-1 utilizes an antibiotic-resistance free plasmid.

COR-1 has been manufactured by VGXI Inc. (The Woodlands, Texas USA) according to current Good Manufacturing Practice (GMP) standards and is supplied in 2mL, 13mm Type I, Clear, Borosilicate w/ blowback vials (Schott #68001022) with Stopper 13mm Westar RS, S2-F451, 4432/50 w/ Flurotec & B2-40, Grey (West # 19700004) and Seal: 13mm Flip-Off® TruEdge® Long (West #54131335, Royal Blue). The solution in the vial is clear and colourless. Please refer to the IB and Certificate of Analysis (COA).

COR-1 is to be stored at -20 degrees Celsius [°C] +/- 5°C,

3.2.1 *Pharmacology*

3.2.1.1 *In vitro* Pharmacology

There is no *in-vitro* experimental data supporting the use of COR-1. In humans, DNA containing unmethylated CpG (such as bacterially-produced plasmid DNA) can selectively enhance Th1 cytokine responses as the CpG repeats may be recognized as pathogen-associated molecular patterns (PAMPs) by toll-like receptor 9 on B cells and certain dendritic cells. While the plasmids in COR-1 were not specifically engineered to contain CpG motifs, unmethylated CpG repeats are present. Unmethylated CpG has been shown to greatly improve the immunogenicity of DNA vaccines, acting as an internal adjuvant. Given that a number of DNA vaccines, including COR-1, containing bacterially-produced plasmid DNA have been tested in humans at much higher dose levels than utilised in our previous Phase I trial and that a number of these vaccines incorporated the pVAX1 plasmid (refer to www.clinicaltrials.gov), which is known to contain unmethylated CpG motifs, it is unlikely that COR-1 will induce a toxic Th1 cytokine profile in HSV-2 positive patients. In addition, oligodeoxynucleotides containing unmethylated CpG motifs designed to be used as adjuvants have been shown to be safe in humans (for reviews see Klinman et al., 2010 or Bode et al., 2011).

3.2.1.2 *In vivo* Pharmacology

In a mouse challenge model of HSV-2 infection, COR-1 has been found to be 100% protective, and 4 out of 9 mice vaccinated had no detectable HSV-2 viral genomes in the dorsal root ganglion at 50x lethal dose $(LD)_{50}$. In a second study, a vaccine with the same inserted gene sequences but a different expression vector proved to be 100% protective and able to confer sterilising immunity in 4 of 10 mice at a challenge dose of 50x LD_{50}. Please refer to the IB for further information.

3.2.2 Preclinical Safety

3.2.2.1 *In vitro* Safety

There have been concerns regarding the use of DNA vaccines including the theoretical possibility of integration into the host genome potentially resulting in tumours. However, no evidence of integration of plasmid DNA into the host genome has been observed in any DNA vaccine trial, making the development of tumour's unlikely (Manam et al., 2000).

Additionally there have been concerns with DNA vaccines that contain antibiotic selection markers having the potential to facilitate the transfer of antibiotic resistance from the plasmid to the host

genome or microbiome. To alleviate this concern, the NTC8485 plasmid used for COR-1 does not use an antibiotic-resistance selection marker.

3.2.2.2 *In vivo* Safety

A single species, Good Laboratory Practice (GLP) repeated dose toxicity study was conducted in Swiss CD1 mice. Four ID injections of the COR-1 vaccine at 30 or 250 mcg/dose were administered over a 6 week period. Dose-related antibody and inflammatory responses were associated with the administration of the 250 mcg/dose. The inflammatory symptoms had reversed after a 3-week treatment free period. There was no evidence of any systemic organ toxicity.

The no observed adverse effect level (NOAEL) based on absence of relevant adverse effects at the highest dose studied in toxicology experiments in mice was 250 mcg/mouse (repeat dose toxicology studies). This equates to a NOAEL of 12.5 mg/kg in the mouse (assuming a 20 g mouse weight). To get the human equivalent dose (HED), the NOAEL is divided by 12.3 (FDA Guidance for Industry: Estimating the Maximum Safe Starting Dose in Initial Clinical Trials for Therapeutics in Adult Healthy Volunteers), which equals a human dose of 1.016 mg/kg or a total dose of around 70mg for a 70kg human. As such, for this study, there is an approximate 140 fold safety margin compared with the 500 mcg dose and approximately 70 fold for the proposed maximum human dose of 1 mg.

3.2.3 Metabolism and pharmacokinetics

3.2.4 Clinical trials of COR-1

The primary objective of the previous Phase I, single ascending dose study was to examine the safety and tolerability of a 3 dose regimen of COR-1 administered intradermally (ID) to healthy, non-HSV-2 infected sero-negative volunteers. In addition the study investigated whether COR-1induced anti-HSV gD2 specific antibodies and/or produced a cell-mediated immune response to gD2 in this population. This dose escalation study aimed to identify an optimal dose of COR-1 required for induction of an efficacious immune response which could protect against future HSV infection.

A total of 22 subjects were enrolled in the study and received at least one dose of COR-1 vaccine. All subjects were included in the Safety Analysis. Twenty subjects received 3 doses of vaccine and were included in the Per Protocol Analysis. A total of 35 Treatment Related Adverse Events (TEAEs) were reported during the study period by 15 subjects (68.2%). A greater number of TEAEs were reported for the 1 mg COR-1 group (19 events) compared with the 10 mcg, 30 mcg, 100mcg and 300mcg COR-1 groups (between 2 and7 events).

One SAE was reported in the 1 mg COR-1 group (clavicle fracture). This event was considered not related to the COR-1 vaccine and no action was taken with the study drug.

The most frequently reported TEAEs were classified under the following System Organ Classes (SOCs):

- Skin and subcutaneous tissue disorders (10 events reported by 3 subjects in the 1 mg COR-1 group only). Skin hyperpigmentation (at the injection site) was the only TEAE reported in this SOC.
- Nervous system disorders (7 events reported by 6 subjects). Events in this SOC were observed in all treatment groups except the 30 mcg COR-1 group

The majority of TEAEs experienced were classified as mild (30 events). Three events reported in the 1 mg COR-1 group were classified as severe. These events were related to the SAE of clavicle fracture. There were no trends identified by treatment group or severity for the TEAEs reported. The majority of TEAEs were considered not related or unlikely to be related to the COR-1 vaccine. 10 events of skin hyperpigmentation observed in the 1 mg COR-1 group were considered definitely related to the COR-1 vaccine.

A total of 143 local ADRs were reported during the study period by 21 subjects (95.5%). The number of local ADRs reported increased with increasing dose of COR-1. Erythema and induration were the most frequently reported injection site reactions, occurring in at least one subject in each treatment group at some point during the vaccination phase. Erythema was reported more frequently in the 100 mcg, 300 mcg and 1 mg COR-1 treatment groups. Induration was reported more frequently in the 1 mg COR-1 treatment group. All injection site reactions were classified as mild in intensity.

A total of 14 systemic ADRs were reported during the study period by four subjects (18.2%). The most frequently reported were headache, fatigue, chills, malaise, pain and pyrexia. There were no trends in the frequency of systemic ADRs by treatment group. All the systemic ADRs reported were classified as mild in severity.

Review of haematology and biochemistry results revealed no trends or notable changes over time following vaccination with COR-1 at any dose level. There were no trends or notable findings in vital signs following each vaccination with COR-1 at any dose level.

A review of the safety data suggests that 3 ID vaccinations of the COR-1 vaccine at three weekly intervals were safe and well tolerated.

No anti-HSV gD2 antibodies were detected in any subject by ELISA, indicating that no subjects had seroconverted to COR-1 during the study based on the utilised method.

IFN-γ production by T cells specific for gD2 antigen was induced in 19 of the 20 subjects who completed the study as planned and was considered a cell-mediated response to the COR-1 vaccine. The response rates were similar in all treatment groups with 100.0% of subjects responding to the COR-1 vaccine in the 10 mcg, 30 mcg, 300 mcg and 1 mg groups and 75.0% responding in the 100 mcg group.

A post hoc analysis was performed on the size of the erythema observed from the photographs taken of the injection sites at one and two days after each vaccination. The analysis revealed that at the higher doses of the vaccine, the size of the erythema increased on day 2 compared to the size of the erythema observed at day 1. This phenomenon was also observed in some subjects receiving the lower doses, but not in the 10 mcg dose. This would indicate a Delayed Type Hypersensitivity reaction, which is a cell mediated immune response and not an antibody-mediated response. This is not inconsistent with the lack of antibody response observed and supports the cell mediated immune response observed.

The analysis of the photographs also revealed a dose response with the size of the erythema observed increasing with increasing doses of the COR-1 vaccine.

Given that no anti-HSV gD2 antibodies were detected in any subject, an optimised dose of COR-1 in order to promote an antibody response to protect against future HSV infection cannot be ascertained. However, COR-1 vaccine did induce a cell-mediated immune response at all doses and this information, together with the safety data and the post hoc analysis of size of the erythema at the injection site, can be used to predict an optimised dose of COR-1 to potentially treat an existing HSV infection.

.

3.3 Rationale for the Study and Dose Selection

COR-1 in the previous Phase I study was well tolerated in healthy volunteers administered three vaccine doses up to 1mg/administration at three weekly intervals. The proposed dose levels/administration to be utilised in this study will not exceed this 1 mg dose.

The dosing interval to be utilised has been increased from three to 4 weekly. The rationale behind this interval change is the anticipation that the subjects may find this schedule easier to maintain. Subjects in this study will receive an additional dose of vaccine half way through the study (Week 24) to both confirm its safety and tolerability and to probe the longevity of the generated immune response and the need for such a booster vaccination. Subjects will be monitored for immunological and clinical signs of vaccine efficacy pre- and post- the booster vaccinations to further understand the effect of this investigational vaccine in subjects with recurrent HSV-2 infection.

4 OBJECTIVES AND ENDPOINTS

4.1 Objectives

The primary objectives of this study are to:

- Evaluate the safety and tolerability of two injection regimens of the HSV-2 DNA vaccine COR-1 compared with placebo, administered by intradermal injection as three, 4-weekly doses followed by a 6-month booster to otherwise healthy symptomatic HSV-2 positive subjects.

The secondary objectives are:

- To investigate the impact of COR-1 on:
    - Induction of an antigen specific humoral and/or cell mediated immune response against gD2.

The exploratory objectives of this study are to evaluate:

- The effect of COR-1 on HSV-2 shedding;
- The incidence of symptomatic HSV-2 genital recurrence; and
- The nature of the immunological responses to COR-1, including local tissue responses.

4.2 Endpoints

Primary endpoints:

The primary endpoints of this study are:

- Incidence and severity of adverse events in each treatment group, including vaccine related adverse events:
    - Incidence and severity of local reactions (soreness, redness, induration, ecchymosis, oedema, itching and paraesthesia) at the site of vaccination;
    - Incidence and severity of systemic reactions (fatigue, myalgia, malaise, fever, rigors, arthralgia, nausea, diarrhoea, light headedness, dizziness, hypersensitivity and headache).

Secondary endpoints:

Immunogenicity:

- Change from Baseline in T-cell and/or antibody responses to HSV-2 gD2 as measured by:
    - ELISA (antibody);
    - Interferon-gamma (IFNγ) enzyme linked immunospot (ELIspot) assays (T-cell responses).

Exploratory endpoints:

Efficacy:

- Number of days with detectable viral shedding after 3-dose vaccination, compared with Baseline.
- Number of days with detectable viral shedding after the booster injection compared with Baseline.
- Symptomatic genital herpes recurrence rate:
- Time to first recurrence of symptomatic genital herpes measured from first administration of study vaccine;
- Number of symptomatic recurrences of genital herpes during the course of the study (to Week 48).
- Viral load level after three dose levels compared to baseline
- Viral load level after booster compared to baseline

Immunogenicity

- Change from Baseline in T-cell and/or antibody response as measured using other exploratory measures of immune function. Such measures of humoral and T-cell responses may include (but are not limited to):
    - T-cell responses (IFNγ, interleukin 2 [IL-2], tissue necrosis factor alpha [TNFα] and other cytokine production by CD4 positive (+) or CD8+ T-cells as measured by intracellular cytokine staining (ICS).
    - Where responses to a whole pool of peptides are obtained, positive responses may be assessed with a matrix of peptides to give information on the breadth of the response and likely epitopes governing the responses.
    - Innate immune responses (e.g. number and function of dendritic cells).
    - Proportion and phenotype of T-cells measured by flow cytometry and MHC tetramer analysis.
    - Influence of HLA type on monitored immune response.
    - Change from baseline of T-cell receptor type
- Histological assessment of the nature of local tissue response at the site of vaccination.

4.3 Study Design

This is a randomized single or multi centre, double blind, placebo-controlled, parallel group pilot study to assess the safety and efficacy of a therapeutic HSV-2 DNA vaccine in HSV-2 positive adults. Subjects will be randomized to receive either COR-1 or placebo using a 3:1 allocation ratio. Treatment assignment to either COR-1 or placebo will be double-blinded. There will be 2 Groups of subjects. Group 1 will receive one injection into each forearm. Once this group has been fully allocated, Group 2 will commence recruitment. Group 2 will receive two injections to the one forearm.

There are two primary reasons for inclusion of the 2 placebo arms:

- To maintain the blind; and
- To describe any natural variation in viral shedding rates between the baseline shedding assessment pre- and post-administration of the placebo.

4.4 Dosing Regimens

All subjects will receive either 2 intradermal injections of 200µL, to the forearm(s) of either:

Group 1-

- Two injections each containing 500mcg of COR-1 with one injection in each arm (two injections in total);
- Two injections containing placebo with one injection in each arm (two injections in total).

Group 2-

- Two injections each containing 500mcg of COR-1 in the one arm; or
- Two injections containing placebo in the one arm;

Intradermal injections will be administered to the patient’s forearms at 4 weekly intervals. Following this a 6 month booster will administered.

The comparison of one versus two arms is designed to test the effect of antigen presentation of vaccine encoded viral antigens occurring in one draining lymph node (2 administrations in one forearm) versus two draining lymph nodes (one administration to each forearm).

4.5 Study Sites

This is a single centre study but this may be revised during the course of the study subject to recruitment.

4.6 Estimated Duration of the Study

40 subjects plus up to 10 replacements will be recruited over a period of up to 6 months. The on-study period per subject is 48 weeks with a screening/ run in of approximately 9 weeks. It is anticipated that the duration of the study will be 18 months including 6months for recruitment and 12 months for clinical conduct, with a further 6 months for reporting.

5 SUBJECT POPULATION

5.1 Selection and Number of Subjects

Otherwise healthy subjects who have at least a 12 month history of having a genital HSV-2 infection with self-reported clinical occurrences of at least 3 and no more than 9 episodes in the 12 months prior to Screening will be selected to participate in the study. If the subject is currently on anti-viral therapy at the time of screening, the subject will need a self-reported recurrence rate of at least 3 and no more than 9 occurrences in the 12 months prior to initiating anti-viral therapy.

The nature of the study and the potential risks will be explained to all candidates. Written informed consent will be obtained from each subject prior to performing any screening procedures/assessments.

Following the Screening assessments, subjects who meet all of the inclusion and none of the exclusion criteria will be eligible to continue in the run-in phase of the study. Inclusion/exclusion criteria will again be assessed at Visit 5 prior to randomisation.

Sufficient numbers of subjects will be screened in order to randomize approximately 40 subjects with COR-1 or placebo in either one or two arms.

There will be no exemptions and subjects must satisfy all eligibility criteria in order to participate.

5.2 Inclusion Criteria

The criteria for entry into the study are:

1. Diagnosis of genital HSV-2 infection confirmed by positive HSV-2 serology or if negative a documented positive HSV-2 pathology result, AND positive PCR for HSV-2 during the Baseline Shedding Assessment prior to randomisation
2. A history of recurrent genital HSV-2 for at least 12 months AND a self-reported history of at least 3 and no more than 9 reported lesion occurrences in the 12 months prior to Screening or, if currently on suppressive therapy, in the 12 months prior to initiation of suppressive therapy.
3. Aged 18 to 50 years, inclusive.
4. Male or female. Women of child-bearing potential must be using two effective methods of contraception and agree to continue to do so from Screening, throughout Investigational vaccine/placebo dosing and for 28 days after the last dose of investigational vaccine/placebo.
5. Has voluntarily given written informed consent.

5.3 Exclusion Criteria

1. Birthmarks, tattoos, wounds or other skin conditions on the forearms which could reasonably obscure injection site reactions.
2. Inadequate venous access to allow collection of blood samples.
3. Female subjects who are breastfeeding and those of reproductive potential with a positive urine beta human chorionic gonadotropin (β-HCG) pregnancy test at either Screening or the Baseline Visit (Visit 5).
4. Received immunomodulating agents (including immunosuppressive agents, interferon or other immune or cytokine-based therapies), and/or systemic chemotherapeutic agents within 60 days of Screening or expected to receive these agents during the course of the study. Stable use asthma inhalers and topical corticosteroids are permitted. All medications will be documented and reviewed for acceptance by the Investigator or a medically qualified nominee.
5. Receiving medication known to have anti-HSV-2 activity within 7 days of the Baseline Shedding Assessment.
6. History of or active serious medical or psychiatric illness which, in the opinion of the Investigator, may impair the ability to provide written informed consent and would interfere with treatment, assessment, compliance with the protocol, or subject safety.

7. History of HSV-1 genital infection, ocular HSV infection, HSV-related erythema multiforme, herpes meningitis or encephalitis.
8. HSV-1 positive PCR result during the Baseline Shedding Assessment.
9. Laboratory blood values:
    a) Haemoglobin <12.0 grams/decilitre (g/dL) for men and <11.0 g/dL for women
    b) Neutrophil count <1000/mm3
    c) Platelet count <80,000/mm3
    d) Aspartate aminotransferase (AST) or alanine aminotransferase (ALT) >1.5 times the upper limit of normal (ULN)
    e) Amylase >1.5 times ULN (unless serum lipase is >1.5 times ULN)
    f) Subjects with an estimated creatinine clearance of <80 mL/minute (min)
    g) INR > ULN
    h) Hepatitis B surface antigen (HBsAg), Hepatitis C Virus (HCV) antibody or HIV antibody positive.
10. Subject has received any vaccine or investigational drug within 30 days prior to Visit 2, or is due to receive subsequent vaccines during the treatment period.
11. Subject has participated in the past in another clinical trial of a vaccination related to infection with HSV (unless it can be verified that the subject received placebo).
12. Any known or suspected allergies to the study drug or its constituents.
13. Subject has a compliance of less than 80% of swab sample collection during the Baseline shedding assessment i.e. immediately prior to randomisation.
14. Previous participation in this study or the previous COR-1 Phase I study.
15. Subject unwilling to abstain from blood donation during the course of the study.

5.4 Other Study Eligibility Criteria Considerations

In order to assess any potential impact on subject eligibility with regard to safety, the Investigator must refer to the relevant document(s) for detailed information regarding warnings, precautions, contraindications, adverse events, and other significant data pertaining to the study product(s) being used in this study. Such documents may include, but not be limited to, the Investigator's Brochure (IB) or equivalent document provided by Admedus Vaccines Pty Ltd.

Subjects will be required to suspend all HSV-2 medication for one week prior to commencing the Baseline Shedding Assessment at Visit 2 and for duration of the trial. This includes anti-viral medications.

Subjects will not be permitted to have any vaccinations during their participation in the study.

Blood donation must be avoided up to Week 25 (Visit 17).

5.4.1 *Contraception*

All women of child bearing potential (defined as sexually mature women who have had menses within the preceding 24 months and have not undergone hysterectomy, bilateral oophorectomy or tubal ligation) must have a negative pregnancy test (with a sensitivity of at least 50 International Units/mL) performed at Screening and prior to each scheduled vaccination.

Women of child bearing potential must agree not to attempt to become pregnant or undergo in vitro fertilization and, if participating in sexual activity that could lead to pregnancy, must use two reliable methods of contraception simultaneously while receiving protocol-specified medication and for 28 days after stopping the medication. Male subjects must agree to use two reliable methods of contraception simultaneously while receiving protocol-specified medication and for 28 days after stopping the medication if their partner is of child bearing potential.

A combination of two of the following methods must be used:

- Condoms (male or female) with or without a spermicidal agent
- Diaphragm or cervical cap with spermicide
- Intra Uterine Device
- Hormonal-based contraception.

Women who are not of reproductive potential (who have been postmenopausal for at least 24 consecutive months or have undergone hysterectomy, bilateral oophorectomy or tubal ligation) are not required to use contraception.

Male subjects and the partners of female heterosexual subjects will be required to use condoms to protect them from infection in the absence of anti-viral medications.

Since all subjects must cease taking anti-viral therapy for at least 7 days prior to Visit 2 and throughout the study, all subjects will be encouraged to use condoms to prevent infection to their partners if they are sexually active. Anti-viral therapy will only be permitted if the Investigator believes that it is in the subject's best interest to commence this and only after the Sponsor has provided documented approval. Antiviral therapy may not be commenced within 7 days of the shedding assessments and during the shedding assessments. Please refer to Section 6.4.9.

5.4.2 *Renal Function*

Renal function eligibility will be determined using an estimated creatinine clearance calculated by Cockroft-Gault Formula.

The Cockcroft-Gault Formula is:

$$\text{Creatinine clearance (mL/min)} = \frac{\text{(140-age) x (Bodyweight in kg) x (0.85 if female)}}{\text{(72 x Serum creatinine (mg/dL)}}$$

5.5 Subject Enrolment

Before subjects may be entered into the study, Admedus Vaccines Pty Ltd requires a copy of the site's written human research ethics committee (HREC)/Research Governance approval of the protocol, informed consent form, and all other subject information, as applicable. All subjects or legally acceptable representatives must personally sign and date the consent form before enrolment.

All subjects who provide written informed consent will be sequentially assigned a Subject Number. This number will be used to identify the subject throughout the clinical study and must be used on all study documentation related to that subject. Subjects who meet the inclusion/exclusion criteria will be randomly assigned to treatment using a permuted block randomization.

All initial screening tests and procedures (except for the Run-in shedding assessment) should be performed within the 14 days prior to Visit 2, unless otherwise indicated. Randomization and enrolment will be conducted as described in Section 6.3.1 and 6.3.2.

5.5.1 *Rescreening*

Subjects with laboratory abnormalities that are exclusionary, but inconsistent with previous history, may be re-tested once. Rescreening of these subjects must be discussed and agreed with the Admedus Vaccines Pty Ltd or designee.

6 SCHEDULE OF ASSESSMENTS AND PROCEDURES

6.1 Study Schedule of Evaluations

The schedule of assessments is presented in Table 1.

6.2 Visit Windows

Every effort should be made to keep to the study schedule. To allow for some flexibility visit windows have been defined and are dependent upon the study stage. The visit windows are as follows:

Table 2– Visit Windows

Study Period	Acceptable Window
Screening/Run-in	± 3 days
Study Period 1	± 3 days except for Visit 5 which has a window of ± 7 days
Study Period 2	± 3 days
Follow up	± 5 days

6.3 Study Procedures / Assessment Periods

The study procedures to be conducted for each subject enrolled in the study are listed below. Additional information on the study procedures is provided in Section 6.4.

Additional visits and/or assessments may be conducted as clinically indicated. For these additional assessments, data may be collected on unscheduled Case Report Form (CRF) pages provided, together with all adverse events (AEs) and concurrent medication that must be recorded throughout the study period.

There are 4 main study periods:

Table 3– Study periods

Study Period	Visit
Screening/Run-in	Visits 1 to 4
Study Period 1	Visit 5 to 14
Study Period 2	Visit 15 to 19
Follow up	Visit 20 to 22

Any deviation from protocol procedures will be noted in the Source Documents and the Sponsor will be notified. The Human Research Ethics Committee/Research Governance for each site will be notified according to their local requirements. Protocol deviations will be reviewed by the study team prior to breaking the blind and deviations will be classified as major or minor.

All laboratory safety tests on blood and urine samples will be performed at local laboratories. Viral shedding assessments and immunological assessments will be performed at a central laboratories specific to the particular assays utilised. . *Refer to the Study Reference Manual.*

Additional visits and/or assessments may be conducted as clinically indicated.

6.3.1 *Screening/Run in visits*

6.3.1.1 Visit 1/Screening

Subjects will be screened within 73 days prior to randomisation to determine eligibility for participation in the study.

The following will be performed and documented at Screening:

- Obtain written informed consent. (This must be completed prior to any other procedure)
- Demographic data (see Section 6.4.1);
- A screening medical/surgical history including HSV-2 history (see Section 6.4.1);
- Review of inclusion/exclusion criteria (see Section 5.2 and 5.3);
- A complete physical examination including:
  - Forearms to be examined and assessed for suitability for ID injections (see Section 7.4.3);
  - Vital signs (see Section 6.4.3);
  - Bodyweight (see Section 6.4.1);
  - Height (see Section 6.4.1);
  - Assessment of all appropriate body systems to determine study eligibility including genital region (see Section 6.4.2);
- Concurrent medication assessment (see Section 7);
- Electrocardiogram (see Section 6.4.4);
- Blood samples for
  - Haematology (see Section 6.4.5);
  - Clinical chemistry including renal function (see Section 6.4.5);
  - Coagulation (see Section 6.4.5);
  - Viral serology for HIV, Hepatitis B and C, HSV-1 and HSV-2 (see Section 6.4.5);
- Urine samples for urinalysis and pregnancy (see Sections 6.4.5 and 6.4.6)

Where possible, it will be requested that the subject will arrange for their local doctor to provide documented evidence of HSV-2 diagnosis and HSV-2 recurrence history.

Subjects meeting the initial inclusion criteria and none of the exclusion criteria will be contacted to arrange Visit 2. Please note, if the subject has been taking anti-viral medications, they will be instructed at this time to cease taking them for the duration of the study. Visit 2 will be scheduled at least 7 days after ceasing the antiviral medications.

6.3.1.2 Visit 2 / Day - 45

At Visit 2, the subject will be provided with education in regard to swab collection, diary completion and outbreak management (See sections 6.4.9, 6.4.10 and 9.10). The site will provide the subject with:

- Swabbing materials including materials for the Run-in shedding assessment and HSV-2 Outbreak kits;
- Written instructions for obtaining genital swabs (gender specific),
- HSV-2 Symptoms and Swabbing Diary and
- Written instructions in case of an HSV-2 Outbreak *(HSV-2 Outbreak Diary*).

Study compliance will be emphasized. The Baseline shedding assessment will commence on the day of Visit 2 in the clinic under supervision of site staff. Subjects will be encouraged to take swabs at the same time each day where possible. (See section 6.4.9).

Adverse event and concomitant medication questioning will be performed. Subjects will be provided with a *Concurrent Medication Form* and *Observation Form* so that they can record information about

medications taken and adverse events between visits. These forms will be reviewed at each clinic visit as part of the adverse event and concurrent medication questioning.

6.3.1.3 Visit 3 / Day - 43

Visit 3 is scheduled two days after Visit 2. This visit can be performed either onsite or via telephone. The purpose of this visit is to ensure that subject is complying with the daily swabbing procedures. The Investigator or designee will question the subject in relation to their swabbing technique, HSV-2 Symptoms and Swabbing Diary completion and the occurrences of HSV-2 outbreaks. If there is any uncertainty regarding the subject's compliance, the subject will be asked to come in for an onsite visit for re-education.

Adverse event and concurrent medication questioning will also take place at this visit (see Section 7 and 8)

.

6.3.1.4 Visit 4 / Day -38

Visit 4 is optional and will be performed 5 days after Visit 3. The purpose of this visit again is to promote compliance with the swabbing component of the protocol. This visit could be performed onsite or via telephone. If there are any doubts about the quality of the swabbing or compliance, the subject will be re-educated via an unscheduled clinic visit. Sites are to be encouraged to keep in contact with their subjects during each swabbing period.

Adverse event and concurrent medication questioning will also take place at this visit (see Section 7 and 8)

6.3.2 Study Period 1 (Treatment period)

6.3.2.1 Visit 5/Day 0/Baseline

On Day 0 prior to vaccine administration, the following assessments will be performed and documented:

- Review of Inclusion/exclusion criteria paying particular attention to compliance with the Run-in swabbing requirements and whether HSV-1 was identified by PCR during the Run-in shedding assessment (see Section 5.2 and 5.3);
- Urine samples for urinalysis and pregnancy testing (see Section 6.4.5 and 6.4.6);
- A symptom directed physical examination (see Section 6.4.2);
- Vital signs(see Section 6.4.3);
- Concurrent medication assessment (see Section 7);
- Adverse event questioning (see Section 8);
- Blood samples for:
  - Haematology (see Section 6.4.5);
  - Clinical chemistry (see Section 6.4.5);
- Blood samples for immunological assessments including:
  - ELIspot (T cell responses specific for HSV-2 gD2) (see Section 6.4.5);
  - ELISA (anti HSV-2 gD2 antibodies) (see Section 6.4.5);
  - Exploratory Immune assays (see Section 6.4.5); and
- Review of HSV-2 Symptoms and Swabbing Diary (see Section 6.4.10).

Once the above assessments have been performed, the patient will be randomised. The vaccination/placebo will be administered and the resultant bleb will be inspected and measured in mm. The results of the bleb inspection will be recorded in the source documents.

Vital signs will be measured at 30 and 60 minutes following the administration of the investigational vaccine/placebo. Subjects will be observed during this time.

Adverse event and concomitant medication questioning will occur.

The subject will be issued with an Injection Site Reaction Diary and instructed to view their injection site(s) at the same time each day and record their findings every day for 7 days.

6.3.2.2 Visit 6 (Week 1) and 8 (Week 5)

The subject will return to the clinic one week following the investigational vaccine/placebo at Week 1 and 5. The following procedures will be performed:

- Symptom directed physical examination (see Section 6.4.2);
- Review and collection of the Injection Site Reaction Diary (see Section 6.4.11);
- Collection of blood samples for:
    - Haematology (see Section 6.4.5);
    - Clinical chemistry (see Section 6.4.5);
- Blood samples for immunological assessments including:
    - ELIspot (T cell responses specific for HSV-2 gD2) (see Section 6.4.5);
    - Visit 8 only; Exploratory Immune assays (see Section 6.4.5).
- Adverse event, occurrences of HSV-2 outbreaks and concurrent medication questioning (see Section 7 and 8);
- If the subject has evidence of a hypersensitivity type reaction at their injection site(s) photographs will be taken in a standardized manner (see Section 6.4.8)

6.3.2.3 Visit 7 (Week 4) and 9 (Week 8)

The subject will receive 2 further investigational vaccinations/placebos in 4 weekly intervals at Visit 7 and 9 according to the assigned randomisation schedule. The following procedures will be performed prior to the administration of the investigational vaccination/placebo:

- A symptom directed physical examination (see Section 6.4.2);
- Vital signs(see Section 6.4.3);
- Urine samples for urinalysis and pregnancy testing (see Section 6.4.5 and 6.4.6);
- Blood samples for:
    - Haematology (see Section 6.4.5);
    - Clinical chemistry (see Section 6.4.5);
- Blood samples for immunological assessments including:
    - ELISA (anti HSV-2 gD2 antibodies) (see Section 6.4.5);

The investigational vaccination/placebo will be administered and the resultant bleb will be inspected. The results of the bleb inspection will be recorded in the source documents.

Vital signs will be measured at 30 and 60 minutes following the administration of the investigational vaccine/placebo. Subjects will be observed during this time.

The subject will be issued with an Injection Site Reaction Diary and instructed to view their injection site(s) at the same time each day and record their findings every day for 7 days.

6.3.2.4 Visit 10 / Week 8 + 48 hours

Visit 10 is to be scheduled for all subjects 48 hours after receiving their third vaccination at Visit 8. There is a +/- 12 hour window for this visit.

All subjects will have their injection sites assessed by the Investigator or designee. Any reactions will be recorded in the source notes and the corresponding CRF page. All subjects regardless of whether they have had an injection site reaction will have a photograph taken in a standardised manner Please refer to Section 6.4.8.

A symptom directed physical examination will be performed (see Section 6.4.2).

Skin biopsies will be taken from all subjects who provide consent at their injection sites. Please refer to Section 6.4.11.

Adverse event, occurrences of HSV-2 outbreaks and concurrent medication questioning will occur (see Section 7 and 8).

The Injection Site Reaction Diary will be reviewed and returned to the subject.

6.3.2.5 Visit 11 / Week 9

Visit 11 will take place one week after the third investigational vaccination/placebo. The following procedures will be performed:

- Symptom directed physical examination (see Section 6.4.2);
- Review and collection of the Injection Site Reaction Diary (see Section 6.4.11);
- Adverse event, occurrences of HSV-2 outbreaks and concurrent medication questioning (see Sections 7 and 8);
- Blood samples for:
    - Haematology (see Section 6.4.5);
    - Clinical chemistry (see Section 6.4.5;
- Blood samples for immunological assessments including:
    - ELIspot (T-cell responses specific for HSV-2 gD2) (see Section 6.4.5);
      Exploratory Immune assays (see Section 6.4.5);
- If the subject has evidence of a hypersensitivity type reaction at their injection site(s) photographs will be taken in a standardized manner (see Section 6.4.8).

The subject’s will receive refresher training on the swabbing collection procedure, HSV-2 Symptoms and Swabbing Diary completion and HSV-2 outbreak management. The Post Vaccination Shedding Assessment will commence on the day of Visit 11 in the clinic under supervision. Subjects will be encouraged to collect swabs at the same time each day for 45 days. Subjects will also be required to complete the HSV-2 Symptoms and Swabbing Diary each day of the shedding assessment to record any HSV-2 symptoms that occur. (See sections 6.4.9 – 6.4.10 and 9).

The subject will be issued with:

- Swabbing materials, and
- HSV-2 Symptoms and Swabbing Diary.

6.3.2.6 Visit 12 / Week 12

Visit 12 will be scheduled 4 weeks after the third vaccination. The following procedures will be performed:

- Symptom directed physical examination (see Section 6.4.2);
- Vital signs (see Section 6.4.3);
- Blood samples for:
    - Haematology (see Section 6.4.5);
    - Clinical chemistry (see Section 6.4.5;

- Blood samples for immunological assessments including:
  - ELISA (anti HSV-2 gD2 antibodies) (see Section 6.4.5)
- Review of the HSV-2 Symptoms and Swabbing Diary (see Section 6.4.10); and
- Adverse event, occurrences of HSV-2 outbreaks and concurrent medication questioning (see Sections 6.4.10, 7 and 8).

The subject's will receive refresher training on the swabbing collection procedure, diary completion and HSV-2 outbreak management.

6.3.2.7 Visit 13 / Week 15

Visit 13 will be scheduled 45 days after Visit 10 at the end of the Post Vaccination Shedding Assessment.

The following procedures will be performed:
- Symptom directed physical examination (see Section 6.4.2)
- Review and collection of the HSV-2 Symptoms and Swabbing Diary (see Section 6.4.10); and
- Adverse event, occurrences of HSV-2 outbreaks and concurrent medication questioning (see Sections 6.4.10, 7 and 8);

6.3.2.8 At Visit 14 / Week 19

The subject will be contacted via the phone at Week 19. Adverse event, the occurrence of HSV-2 outbreaks and concurrent medication questioning will take place. Subjects will be reminded about their next scheduled appointment.

6.3.3 Study Period 2 (Booster injection)

6.3.3.1 At Visit 15 / Week 23

Visit 15 is a clinic visit and will take place one week prior to the Booster investigational vaccination/placebo. The following procedures will take place:

- Symptom directed physical examination (see Section 6.4.2);
- Collection of blood samples for:
  - Haematology (see Section 6.4.5);
  - Clinical chemistry (see Section 6.4.5);
- Adverse event, occurrences of HSV-2 outbreaks and concurrent medication questioning (see Section 6.4.10, 7 and 8).

6.3.3.2 At Visit 16 / Week 24

The subject will receive the Booster investigational vaccination(s)/placebo at Week 24 following the completion of the safety assessment procedures including:

- A symptom directed physical examination (see Section 6.4.2);
- Vital signs(see Section 6.4.3);
- Adverse event, occurrences of HSV-2 outbreaks and concurrent medication questioning (see Sections 6.4.10, 7 and 8);
- Blood samples for:
  - Haematology (see Section 6.4.5);

  - Clinical chemistry (see Section 6.4.5);
- Blood samples for immunological assessments including:
  - ELIspot (T-cell responses specific for HSV-2 gD2) (see Section 6.4.5);
  - Exploratory Immune assays (see Section 6.4.5)
  - ELISA (anti HSV-2 gD2 antibodies) (see Section 6.4.5)
- Urine samples for urinalysis and pregnancy testing (see Section 6.4.5 and 6.4.6);

The investigational vaccination/placebo will be administered and the resultant bleb will be inspected. The results of the bleb inspection will be recorded in the source documents.

Vital signs will be measured at 30 and 60 minutes following the administration of the investigational vaccine/placebo. Subjects will be observed during this time.

The subject will be issued with an Injection Site Reaction Diary and instructed to view their injection site(s) at the same time each day and record their findings every day for 7 days.

6.3.3.3 At Visit 17 / Week 25

Visit 17 will take place one week after the Booster investigational vaccine/placebo. The following procedures will be performed:

- Symptom directed physical examination (see Section 6.4.2);
- Review and collection of the Injection Site Reaction Diary (see Section 6.4.10);
- Collection of blood samples for:
  - Haematology (see Section 6.4.5);
  - Clinical chemistry (see Section 6.4.5);
  - ELIspot (T-cell responses specific for HSV-2 gD2) (see Section 6.4.5);
  - Exploratory Immune assays (see Section 6.4.5);
- Adverse event, occurrences of HSV-2 outbreaks and concurrent medication questioning (see Sections 6.4.10, 7 and 8);
- If the subject has evidence of a hypersensitivity type reaction at their injection site(s) photographs will be taken in a standardized manner (see Section 6.4.8).

The subject’s will receive refresher training on the swabbing collection procedure, HSV-2 Symptoms and Swabbing Diary completion and HSV-2 outbreak management. The Post Booster Vaccination Shedding Assessment will commence on the day of Visit 17 in the clinic under supervision. Subjects will be encouraged to collect swabs at the same time each day where possible for 45 days. Subjects will also be required to complete the HSV-2 Symptoms and Swabbing Diary each day of the shedding assessment to record any HSV-2 symptoms that occur. (See sections 6.4.9 – 6.4.8 and 9).

6.3.3.4 At Visit 18 / Week 28

Visit 18 will be scheduled 4 weeks after the booster vaccination. The following procedures will be performed:

- Symptom directed physical examination (see Section 6.4.2);
- Vital signs (see Section 6.4.3);
- Collection of blood samples for:
  - Haematology (see Section 6.4.5);
  - Clinical chemistry (see Section 6.4.5);
- Blood samples for immunological assessments including:
  - ELISA (anti HSV-2 gD2 antibodies) (see Section 6.4.5);
- Review of the HSV-2 Symptoms and Swabbing Diary (see Section 6.4.10); and
- Adverse event, occurrences of HSV-2 outbreaks and concurrent medication questioning (see Sections 6.4.10, 7 and 8).

The subject's will receive refresher training on the swabbing collection procedure, HSV-2 Symptoms and Swabbing Diary completion and HSV-2 outbreak management.

6.3.3.5 At Visit 19 / Week 31

The subject will return to the clinic for Visit 19 will be scheduled 45 days after Visit 17 after completing the Post Booster Shedding Assessment.

The following procedures will be performed:
- Symptom directed physical examination (see Section 6.4.2);
- Review and collection of the HSV-2 Symptoms and Swabbing Diary (see Section 6.4.10); and
- Adverse event, occurrences of HSV-2 outbreaks and concurrent medication questioning (see Sections 6.4.10, 7 and 8).

6.3.4 Follow up

6.3.4.1 Visit 20 / Week 36 and Visit 21 / Week 42

At Visit 20 and Visit 21, the site staff will telephone the subject and collect the following information:
- Adverse event, occurrences of HSV-2 outbreaks and concurrent medication questioning will occur. (See Sections 6.4.10, 7 and 8).

6.3.4.2 Visit 22 / Week 48 / End of Study (or early withdrawal from treatment visit)

The end of study visit will be conducted in the clinic. The following tests will be performed and documented as indicated on the CRF:

- Symptom directed physical examination (see Section 6.4.2);
- Vital signs (see Section 6.4.3);
- Adverse event, occurrences of HSV-2 outbreaks and concurrent medication questioning (see Sections 6.4.10, 7 and 8);
- Blood samples for
  - Haematology (see Section 6.4.5);
  - Clinical chemistry (see Section 6.4.5);
- Blood samples for immunological assessments including:
  - ELIspot (T cell responses specific for HSV-2 gD2) (see Section 6.4.5);
  - Exploratory Immune assays (see Section 6.4.5)
  - ELISA (anti HSV-2 gD2 antibodies) (see Section 6.4.5)
- Urine samples for urinalysis and pregnancy (see Sections 5 and 6.4.6)

6.4 Details of Scheduled Assessments

6.4.1 Demographic Data, Medical/Surgical History, Medication History and Concurrent Medication

Demographic data will include gender, ethnicity and date of birth.

The medical history will include any diagnosed medical conditions or surgical history. It will also include the existence of any birthmarks, tattoos, wounds or other skin conditions on the forearms.

Where possible, subjects will be asked to provide documented evidence from their local doctor that they have a diagnosis of HSV-2 and details regarding their HSV-2 outbreak recurrence history.

All genital herpes medication history, and concurrent medication, including non-prescription medication, will be collected and recorded in the CRF.

6.4.2 Physical examination

A complete physical examination (including head, ears, nose, throat, lungs, lymph nodes, heart, abdomen, skin and genital region) will be conducted to determine study eligibility at screening. A symptom directed physical examination will be performed at subsequent visits as defined in Section 6.3. The symptom directed physical examinations will be performed based on signs and symptom the subject has reported verbally and/or documented in the diary booklets. A symptom directed physical examination may also be performed if in the opinion of the Investigator it is required.

Prior to each injection, the forearm that is to be injected will be examined and findings from this examination will be documented.

At each visit, following Visit 5, the subject’s injection sites will be inspected and any reactions will be graded. Please refer to Section 8. Any reactions should also be captured as adverse events within the CRF.

6.4.3 Vital signs

Vital signs to be measured are:

- body temperature (degrees Celsius) aural;
- respiratory rate;
- pulse rate;
- blood pressure; and
- Height and body weight will be measured and documented at the Screening visit.

Vital signs will be measured after the subject has been in a resting semi- supine position for at least 3 minutes. If there are abnormalities, at least two further repeat BP measurements will be performed to confirm results. On visits where the subject is scheduled to receive a vaccination, the vital signs must be performed prior to receiving the investigational vaccine. If the subject’s temperature is > 38 degrees Celsius, the investigational product will be withheld for up to one week. Please see Section 8.6.

On dosing days, vital signs will also be performed at 30 and 60 minutes post dosing.

Body weight (kilogram [kg], without shoes), and Height (centimetres [cm], without shoes) will be measured and documented at the Screening visit.

6.4.4 Electrocardiograms

The ECG recordings in this clinical trial will be performed by the Investigator or designated nominee. Repeat measurements will be performed if there are any clinically significant abnormalities observed or artifacts are present. All ECG recordings will be reviewed by the Investigator or medically qualified nominee who will document whether the tracing is normal or abnormal. If abnormal the Investigator or medically qualified nominee will document whether the abnormality is assessed as clinically significant or not.

The ECG recordings will be performed once the subject has been resting semi-supine for at least 10 minutes. The following parameters will be reported: QRS, QT, QTc, RR and PR intervals. The formula for calculating the corrected QT interval in respect to the heart rate will also be calculated using either Bazett or Fredericia formulas. If this isn’t automatically calculated by the ECG machine, the following formulas will be used:

- Bazett’s formula: $QT / (RR)^{0.5}$

  [Observed QT interval divided by root of RR interval, in seconds]

- Fridericia's formula: QT / $(RR)^{0.33}$

  [Observed QT interval divided by cube root of RR interval, in seconds]

All tracings will be printed out and retained with the subject's source notes. Any clinically significant out of range results will be recorded in the subject's CRF and the subject will be excluded from the study.

6.4.5 Blood and urine samples for laboratory tests

Blood and urine will be collected at Screening (Visit 1), Baseline (Visit 5), and scheduled clinic Visits 7, 9, 16 and 22. Blood will be collected at scheduled clinic Visits 6, 7, 8, 9, 11, 12, 15, 16, 17, 18 and 22. Samples for laboratory tests should be collected within the visit window. Detailed instructions on the handling and processing of specimens are located in the Study Reference Manual. The following will be collected at the times specified in Section 6.3.

Haematology:	*Haemoglobin, haematocrit, red blood cell (RBC) and RBC morphology, white blood cell and differential white blood cell count, platelet count,*
Chemistry:	*Sodium, potassium, chloride, bicarbonate, BUN, creatinine, total protein, albumin, lipase, phosphorus, AST, ALT, GGT, alkaline phosphatase, total bilirubin, glucose, CPK, calcium, uric acid, GFR**
Coagulation:	*Prothrombin time (PT), partial thromboplastin time (PTT), and International Normalized Ratio (INR).*
Viral Serology:	*HIV, HCV and HBV, HSV-1 and 2 (at screening).*
Urinalysis:	*Specific gravity, protein, glucose, blood leukocytes, pH, ketones, bilirubin, urobilinogen, nitrites, protein, and blood..*
Urine pregnancy Testing:	*Human chorionic gonadotropin*
Immunological	*Interferon-gamma (IFNγ) enzyme linked immunospot (ELIspot) assays (T-cell responses specific for HSV-2 gD2).*<br>*ELISA (anti HSV-2 gD2 antibodies)*<br>*Exploratory endpoints include measures of humoral and T-cell responses and/or sequencing T-cell receptor gene repertoire and/or sequencing human leukocyte antigen (HLA) gene.*

**As determined using the Cockcroft and Gault calculation. Please refer to Section 5.4.2.*

6.4.6 Pregnancy tests

Urine pregnancy testing will be conducted for female subjects at Screening, before administration of each dose of investigational vaccine/placebo and at the end of the study. If positive, a serum pregnancy test will be conducted and the investigational vaccine/placebo will not be administered until the result of this is known. If the result of the serum pregnancy test is negative, administration of study vaccine/placebo will continue. The subject will be re-educated regarding the contraception requirements for this study. If the result of the pregnancy test is positive, the subject will be withdrawn from the study.

6.4.7 Intradermal injection of COR-1 or placebo

Prior to each injection, the forearm(s) that is to be injected will be examined and findings from this examination will be documented. The proposed injection site will be approximately 5 cm below the elbow joint on the inside forearm.

If the subject is allocated to Group 2, that is, to receive 2 injections on the same arm, the second injection site will be located 5 cm away radially, not transversely, from the first injection site. Subject's in Group 2 may choose which arm will be injected, however where possible the non-

dominant arm is preferred. Where possible the same arm will be injected throughout the course of the study.

The skin should be free of blemishes, scars, heavy hair, veins, moles, skin conditions, sores or tattoos that have the potential to interfere with the detection of local reactions.

Areas of skin that involve injection site reactions where possible should be avoided for subsequent vaccinations. Another suitable site should be chosen at least 5 cm away from the existing reaction.

Intradermal injections will be carried out by an individual experienced with ID injections as per Appendix 1.

6.4.8 Photos of injection site (s)

At Visits 6 and 8, the Investigator or designee will review the subject and inspect their injection sites for evidence of local reactions. If an injection site reaction has occurred, the Investigator or designee the site will take a photo. An injection site reaction is defined as an area of erythema and/or swelling/induration.

At Visits 10, all injection sites will be photographed regardless of whether there is a hypersensitivity type reaction or not.

In addition, if at other times the Investigator or designee assesses an injection site reaction that is unusual and/or not expected, this will also be photographed.

All photographs will be captured in a standardized manner. Please refer to the Study Reference Manual for details of the procedure for image acquisition.

6.4.9 Genital viral shedding assessments

Subjects will be educated regarding genital swab collection, diary booklet completion and HSV-2 outbreak management. The requirement for compliance in these aspects of the study should be reinforced as often as possible. Additional unscheduled visits may be scheduled for educational purposes should the Investigator or designee judge that this is required.
Daily genital swabs to detect viral shedding will be performed for 45 days commencing as follows:

1. Run-in Shedding Assessment - prior to the first vaccination.
2. Post Vaccination Shedding Assessment - commencing one week following the third investigational vaccine/placebo (Study Period 1); and
3. Post Booster Shedding Assessment - commencing one week after the booster vaccination/placebo (Study Period 2).

Prior to commencing the first assessment period, each subject will be given written gender specific instructions for obtaining genital swabs.

The subject will be instructed to take the swabs at the approximately the same time of the day. Ideally this will be before bathing.

If the subject does not collect a swab at the planned time they will be instructed to obtain a swab as soon as they are able to. (Provided it is on the same day). If this is not until the following day, they will be instructed to document in the diary booklet that a swab wasn’t collected, and the reason for not doing so.

Subjects will be given sufficient swabs and courier supplies to enable them to collect the required number of daily swabs. Each subject will receive:

- Swabs,
- Tubes containing the transport medium,
- Spare swabs in case of error,
- Sample biological bags,
- Prepaid/pre-labelled return mail packs,
- Requisition forms;
- Sample labels;
- Torch (for inspection of the genital region);
- Mirror (for inspection of the genital region);
- ‘HSV-2 Outbreak’ kits for when a subject develops lesions. This kit includes:
    - Swabs;
    - Tubes containing the transport medium,
    - Sample biological bags,
    - Prepaid/pre-labelled return mail packs
    - Requisition Forms; and
    - Sample labels.

Gender specific instructions for obtaining genital swabs will be provided to subjects at Visit 2.

In summary, men will be instructed to obtain the following swabs:
1. Penile skin
2. Perianal and rectal areas.

Women will obtain the following swabs:
1. Vaginal and vulval area;
2. Perianal and rectal areas.

If subjects have previously experienced lesions outside the above mentioned areas, they will be provided with additional swab(s) to enable this region to be swabbed daily providing the lesion is below the waist.

Subjects who do not collect at least 80% of swabs during the Baseline Shedding Assessment (i.e. at least 36 days) will not meet eligibility criteria for the study (see Exclusion criterion 14).

Subjects who test positive for HSV-1 during the Baseline Shedding Assessment will be excluded from this study.

Swabs will be sent directly to the central laboratory each week for analysis. Prior to shipment, the swabs will be kept in the subject’s refrigerator in sealed specimen bags.

Should a subject develop an HSV-2 Outbreak during a scheduled 45 day swabbing assessment, the subject should not interrupt this assessment. Additional swabs taken from the lesions will be collected. Please see section 6.4.10.

6.4.10 Diary Booklets

Subjects will be provided with a diary after each vaccination to collect injection site reactions and at the commencement of each swabbing assessment to collect HSV-2 symptoms.

Injection Site Reaction Diary:

The aim of this Diary is to collect information about injection site reactions for 7 days following each vaccination. Information that is to be collected includes the presence of:

- Pain/tenderness;
- Swelling/induration;
- Erythema; or
- Other findings.

Subjects will be provided with a ruler to enable the subject to measure and record the size of any injection site reactions that occur in millimetres (mm). Subjects will be instructed to measure the longest diameter of the reaction area. Instructions on the measurement of reactions will be provided to the subjects within their Diary booklet. Injection site reactions will be graded in accordance with the FDA Guidance for Industry document entitled *Toxicity Grading Scale for Healthy Adult and Adolescent Volunteers Enrolled in Preventive Vaccine Clinical Trial* (2007) except for pain and tenderness (see Section 9.2.1).

The Injection Site Reaction Diaries will be issued and collected at the following visits:

- Vaccination 1- Issued at Visit 5, collected at Visit 6;
- Vaccination 2- Issued at Visit 7 and collected at Visit 8;
- Vaccination 3 – Issued at Visit 9, reviewed at Visit 10, and collected at Visit 11; and
- Booster Vaccination/Placebo – Issued at Visit 16 and collected at Visit 17.

When each Diary is returned by the subject, the site staff will check that it is complete and legible while the subject is in the clinic.

<u>HSV-2 Symptom Diary:</u>
At the start of each shedding assessment, the subjects will be provided with a Diary to record HSV-2 symptoms. A HSV-2 Symptom Diary will be issued to the subjects at the following time points:

- Visit 2 (Day -45),
- Visit 11 (Week 9), and
- Visit 17 (Week 25).

The subjects will be instructed to inspect the genital region for lesions daily and document each day whether they have experienced any genital herpes symptoms. A mirror and torch will also be provided for this purpose. There are provisions in the Diary to record the presence of:

- Redness;
- Swelling;
- Soreness;
- Itching; or
- Ulceration/blisters.

When each Diary is collected, the site staff will check that it is complete and legible while the subject is in the clinic. Completed diaries will be filed with the source notes.

The Diary Booklets will have provisions for subjects to record concomitant medications and adverse events.

<u>HSV-2 Outbreak Diary</u>:

At Visit 2 subjects will be provided with HSV-2 Outbreak Diaries. The purpose of these diaries is to record further information about their HSV-2 outbreak. The site staff will check at each visit that the subject has sufficient blank copies of this diary. Please refer to Section 8.11.

6.4.11 Punch biopsies

The vaccine has been demonstrated to produce a reddened thickening at the site of administration which appears after 24 hours and is maximal at 48 hours. This is characteristic of a delayed type hypersensitivity reaction, which would indicate induction of an appropriate antigen specific cell mediated immune response. However, it could represent some other vaccine induced non-specific or inflammatory response. To establish the nature of the response, immunohistochemistry of the lesion at 48 hours may provide evidence of a T-cell infiltrate, oedema and or a neutrophilic infiltrate which would not be associated with antigen specific immunity).

All subjects who provide written informed consent at Screening will have a skin biopsy taken from the injections site area at Visit 10 for immunohistochemical analysis to assist in determining the type of immune response occurring.

Please refer to Appendix 2 for further information.

6.4.12 Handling and processing of biological specimens

All personnel involved in collecting and handling biological specimens should follow appropriate precautionary procedures for handling biohazardous materials as currently recommended by the national regulatory authority. The processing of all biological specimens will be in accordance with the site's standard practices.

Laboratory safety evaluations, including haematology, clinical chemistry, urinalysis and coagulation will be performed by the sites local laboratory.

Immunological testing will be performed by:

Table 4 – Immunological and Virological Testing Laboratories

	Laboratory
ELIspot	*TBA*
HSV-2 serology	*TBA*
HLA Typing	*TBA*
ICS	*TBA*
Skin biopsy	*TissuPath*
Swabs for genital shedding	*Victorian Infectious Diseases Research Laboratory (VIDRL)*
PBMC preparation	*Q-Pharm*

6.5 Randomisation process

Subjects will first be randomized to one of 2 injection regimens using a 3:1 allocation ratio.

There will be 2 patient groups. Group 1 will be recruited first. Once this group has been fully allocated, Group 2 will open for recruitment.

Group 1 patients will receive one injection to each forearm (i.e. two injections in total at each vaccination) of either:

- Sub-group A: 15 subjects will receive 2 x 500mcg of COR-1;
- Sub-group B: 5 subjects will receive 2 x placebo.

Group 2 patients will receive two injections to the one forearm (i.e. two injections in total at each vaccination) of either:

- Sub-group C: 15 subjects will receive 2 x 500mcg of COR-1;
- Sub-group D: 5 subjects will receive 2 x placebo.

Under no circumstances are the subjects who enrol in this study are permitted to be re-randomised and re-enrolled for a second course of treatment for this protocol.

6.6 Blinding

Since the investigational vaccine is more viscous as compared to the placebo, it will not be possible to blind the study site member who administers the investigational vaccine. The pharmacy team members will also be unblinded. At the commencement of the study, the blinded investigational vaccine administrators will be identified and trained. There will be at least 2 investigational vaccine administrators at each site who have received training in the delivery of intradermal injections. The investigational vaccine administrators will not be permitted to be involved in patient assessments or management throughout the trial. Additionally they will not discuss what they believe the subject is receiving with either the subject or other staff members.

All other site staff and the subject will be blinded to the study treatment, COR-1 or placebo. Admedus Vaccines Pty Ltd and its representatives will also be blinded to the study treatment throughout the course of the study. The investigational vaccine administrator’s will make every attempt to ensure that both the subject and the blinded team members do not observe the intradermal injection as this may threaten the integrity of the blind. The subject may place their forearm through a screen which blocks their view or may be asked to turn away during the injection. Similarly site staff will be instructed to not observe the administration of the investigational vaccine/placebo. This will be highlighted during the site initiation visit and throughout the study.

An unblinded monitor will perform drug accountability according to the Monitoring Plan.

The dose and injection regimen will not be blinded.

6.7 Method of Unblinding

6.7.1 Medical Emergency

Code break envelopes will be provided to participating sites. The Investigator may request the treatment assignment in emergency situations only. The breaking of the blind will only be sanctioned where knowledge of the study medication treatment will affect subject management. The Investigator will discuss with the Sponsor prior to any un-blinding taking place, unless immediate knowledge of the treatment allocated is required for the Subject’s care.

If the code is broken a note to file will be generated by the individual who broke the code explaining the reason and date that the blind was broken. Included in this document should be details of the person who authorised the code break, and the identity of the study product allocated to the subject. This must be countersigned by the Investigator.

Reason(s) for un-blinding will be clearly documented and the details included in the study report.

6.7.2 End of Study

The randomisation code will be broken by the Study Statistician once data entry has been completed, the database locked, and the per-protocol population for analysis established and the Statistical Analysis Plan (SAP) finalised and approved. Admedus Vaccines Pty Ltd will provide written permission to the Study Statistician prior to the breaking of the randomisation code.

6.8 Formulation

6.8.1 Investigational products

Admedus' HSV vaccine (COR-1) is a 1:1 mixture of two DNA plasmids. Both plasmids comprise the Nature Technology Corporation (NTC) expression vector NTC8485 minus the enhanced green fluorescent protein sequence. The first has a codon optimised DNA sequence insert encoding full length glycoprotein D from HSV-2 (gD2) (named NTC8485-O2-gD2) and the other with a codon optimised DNA sequence insert encoding one ubiquitin repeat fused to the N-terminal end of amino acids 25-331 of truncated gD2 (named NTC8485-O2-Ubi-gD2tr). In summary:

1. COR-1A: plasmid DNA NTC8485-O2-gD2
2. COR-1B: plasmid DNA NTC8485-O2-UgD2tr

It is formulated in sterile, isotonic and endotoxin free Tris (hydroxymethyl) amino methane hydrochloric acid and Ethylenediaminetetraacetic acid (EDTA) at pH 8 (TE buffer). Placebo vaccine is sterile, isotonic and endotoxin free TE buffer.

Each COR-1 single use vial contains 1,250 mcg in 0.5mL

Table 5: Investigational Products

**Name:**	**COR-1**
Characteristics & Physical State:	Clear, colourless solution free from visible particulate matter containing 500 mcg of COR-1
Supplied by:	VGXI Inc. (The Woodlands, Texas, USA)
Storage Conditions:	Store at minus 20 degrees Celsius +/- 5 degrees
Package	2 mL clear vial
**Name:**	**Placebo**
Characteristics & Physical State:	Clear, colourless solution free from visible particulate matter containing TE buffer.
Supplied by:	Sypharma Pty Ltd. (27 Healey Rd, Dandenong, Victoria 3175)
Storage Conditions:	Store at 20 degrees Celsius +/- 5 degrees
Package	2 mL clear vial

Please refer to the IB and Certificate of Analysis (CoA).

6.8.2 Supply, packaging and labelling, storage and handling

COR-1 will be manufactured under the current Good Manufacturing Practice (cGMP) conditions by VGXI Inc. 2700 Research Forest Drive, Suite 180, The Woodlands, TX 77381, USA under licence from Admedus Vaccines.

The batch number, expiry date and documented compliance with specification results will be included in the COR-1 certificate of analysis. Evidence of cGMP will also be made available. The vaccine will be supplied to the study site after receipt of required documents in accordance with the Sponsor authorised release procedures. Clinical trial labels will comply with Annex 13 of the Australian Code

of Good Manufacturing Practice for Medicinal Products – Manufacture of Investigational Medicinal Products.

COR-1 must be stored in a secure area with access limited to the pharmacist and authorized staff. The vials and outer packaging will be stored frozen at -20 +/-5 ^{o}C, and thawed to room temperature prior to use. The COR-1 vials contain 1,250mcg per 0.5 mL.

The clinical study personnel must maintain a temperature log for the freezer which houses the investigational product. Daily minimum and maximum temperatures will be recorded in the temperature logs.

The packaging lot numbers will be recorded on the investigational product accountability record.

The pharmacy team will be unblinded to the subject treatment allocation. There will be an unblinded monitor who will perform IP accountability/reconciliation.
.
Refer to the Pharmacy Manual for detailed information regarding the storage, preparation and administration of COR-1.

At a minimum, the immediate packaging should include the following information:

- Sponsor name;
- Protocol number;
- Name/drug code;
- Dose/product strength;
- Batch number or batch identifier (in case of double blind studies);
- Subject ID; and
- Dosage form.

6.8.3 Dosage and administration of test drugs

Subjects are to receive either one or two intradermal injections at four week intervals for 3 vaccinations and a Booster vaccination at approximately 6 months from Baseline (see Section 6.3). Vaccinations will occur at: Baseline, Week 4, 8 and a booster will be administered at Week 24. Subjects in Group 1 will receive the intradermal injections into each forearm. Subjects in Group 2 will receive the intradermal injections to the one forearm.

Further details on the administration of study vaccine will be provided in the Study Reference Manual.

Prior to each injection, the forearm(s) that are to be injected will be examined and findings from this examination will be documented. The proposed injection site will be 5-10 cm below the elbow joint. The skin should be free from blemishes, scars, heavy hair, veins, moles, skin conditions, sores or tattoos etc. that have the potential to interfere with the detection of local injection site reactions.

Subjects in Group 2 will receive 2 injections to the same forearm, the second injection will be administered approximately 5 cm away radially, not transversely, from the first injection.

Where there is an ongoing injection site reaction an alternative site should be utilised at subsequent vaccination time points. The subsequent vaccination should be located at least 5 cm away from the previous injection site.

Intradermal injections will be carried out by an individual experienced with ID injections as per Appendix 1.

The subject will be instructed to extend their forearm(s) for 10-15 minutes after receiving their vaccination or until the bleb subsides. The injection site will be left open. No cotton wool, adhesive bandages etc. are to be applied over the injection site. The subject will be instructed to avoid touching the injected area(s).

The Investigator or nominee will make the following observations and document findings in the source notes:

- The size of the 'bleb' immediately after injecting in millimetres,
- The size of the 'bleb' during the period that the subject must keep the forearm extended,
- Whether there is an absence of a 'bleb,'
- If the injected material leaked from the skin before 15 minutes,
- If there are any unusual appearances at the injection site.

The subject will be observed for one hour following each vaccination.

6.8.4 Dispensing and accountability

COR-1 is supplied for use only in this clinical study and should not be used for any other purpose. The Investigator's nominee will prepare the vaccine under the Pharmacy Manual set out in a separate document.

Subjects will require the following numbers of vials according to their treatment allocation:

Table 6. Subject group dispensing requirements

Group	Sub-Group	Treatment dose	Number of COR-1 vials to dispense	Number of placebo vials to dispense
1	A	2 x 500 mcg COR-1	1	0
	B	2 x Placebo	0	1
2	C	2 x 500 mcg COR-1	1	0
	D	2 x Placebo	0	1

Should the pharmacist have difficulties drawing up 2 doses of the investigational product/placebo from the one vial, 2 vials may be dispensed.

The Investigator will be responsible for maintaining accurate records for all study medications dispensed and returned. The inventory must be available for inspection by the study monitor. Study product supplies, including partially used or empty vials and the dispensing logs, must be accounted for by the study monitor and returned to the drug repository and at the end of study returned to the Sponsor.

If requested in writing by the Sponsor, unused study medication supplies may be destroyed by the Investigator provided such disposition can be performed safely. Records shall be maintained by the Investigator or any such alternate disposition of the study medication. These records must show the identification and quantity of each unit disposed of, the method of destruction (taking into account the requirements of local law), and the person who disposed of the test substance. Such records shall be submitted to the Sponsor.

7 CONCURRENT MEDICATIONS AND TREATMENTS

At each study visit or contact, the Investigator should question the subject about any medication taken including vitamin supplements and herbal remedies. Any concurrent medications will be recorded in the subject's records and the CRF. Any changes in doses or introduction of a new medication during the course of the study will also be recorded.

7.1 Special Dietary Requirements

There are no special dietary requirements.

7.2 Concurrent Medications/Treatments Not Permitted

7.2.1 Prior to study entry

Refer to Exclusion Criteria in Section 5.3

7.2.2 During the study dosing period

Throughout the study medication dosing period, subjects <u>may not</u> receive any of the following concomitant medications:

- Immunomodulating agents (including immunosuppressive agents, interferon or other immune or cytokine-based therapies),and/or systemic chemotherapeutic;
- Chronic treatment with immune-suppressive therapy (stable use asthma inhalers and topical corticosteroids are permitted);
- Anti- viral medications, (both orally and topically) must not be taken during the swabbing assessment periods of the study. The subject should abstain from taking antiviral medications at all other times during their participation in the study unless in the judgement of the Investigator it is in the subject's best interest and Sponsor approval has been obtained prior;
- Topical application of any lotions or the like to the genital region which may interfere with swab collection during the swabbing assessment periods;
- Subjects also should not receive any other vaccines during their participation in this study.

8 ADVERSE EVENTS AND TOXICITY MANAGEMENT

8.1 Safety Parameters

Safety parameters will include adverse events, vital signs, and clinical laboratory tests.

8.2 Adverse Events

An adverse event or adverse experience (AE) is any untoward medical occurrence in a subject or clinical investigation subject administered an investigational product (whether it is the experimental product or the control) and which does not necessarily have a causal relationship with the investigational product. An AE can therefore be any unfavourable and unintended sign, symptom, or disease temporally associated with the use of a medicinal product, whether or not considered related to the medicinal product. Pre-existing events, which increase in frequency or severity or change in nature during or as a consequence of use of a drug in human clinical trials, will also be considered as adverse experiences. AEs may also include pre- or post-treatment complications that occur as a result of protocol-mandated procedures (e.g. invasive procedures such as biopsies).

Any AE (i.e. a new event or an exacerbation of a pre-existing condition) with an onset date after obtaining written informed consent up to the last day on study (including the follow-up, off study medication period of the study), should be recorded as an AE on the appropriate CRF page(s).

An AE **does not** include:

- Medical or surgical procedures (e.g. surgery, endoscopy, tooth extraction, transfusion); the condition that leads to the procedure is an adverse event;
- Pre-existing diseases or conditions present or detected prior to start of study product administration, that do not worsen;
- Situations where an untoward medical occurrence has not occurred (e.g. hospitalisation for elective surgery, social and/or convenience admissions);
- Overdose of either study product or concomitant medication without any signs or symptoms unless the subject is hospitalised for observation.

The subject will be instructed on the completion of the Diary Booklets that will serve as supporting documentation for solicited signs or symptoms after each vaccination including local injection site reactions and systemic symptoms and also genital HSV-2 symptoms (during swabbing assessments). Entries in the diary booklets shall be evaluated and graded for severity and causality by the Investigator (see Section 8 and Appendix 2). Clinically significant signs and symptoms reported in the diary card will be entered in the CRF.

Injection site reactions may include:

- Pain/tenderness;
- Erythema;
- Swelling/Induration; or
- Other.

Systemic symptoms may include:

- Hypersensitivity
- Headache;
- Fatigue;
- Myalgia;
- Malaise;
- Fever;
- Rigors;
- Arthralgia
- Nausea/Vomiting;
- Diarrhoea;
- Light headedness;
- Dizziness.

Additionally the subjects will be provided with HSV-2 Outbreak forms should they have a genital herpes recurrence outside the shedding assessment periods

8.2.1 Assessment of AEs

All AEs will be assessed by the Investigator and recorded on the appropriate CRF page, including the date of onset and resolution, severity, relationship to investigational vaccine/placebo, outcome and action taken with study medication. See Appendix 2 for details of toxicity grade scales (TGS) relevant to vital signs, local reactions to injectable product, systemic signs and symptoms, systemic Illness, and clinical laboratory adverse events.

In addition, injection site reactions will be graded by an Investigator or designee, according to severity. It is anticipated that the subjects may find it difficult to differentiate pain and tenderness (Norquist et. al. 2012). For this reason pain and tenderness have been combined with the following grading scale:

Table 7 – Grading for Pain and Tenderness at injection sites

Mild<br>Grade 1	Moderate<br>Grade 2	Severe<br>Grade 3
Awareness of symptoms but easily tolerated	Discomfort enough to cause interference with usual activities	inability to do work or usual activities

For all other reported adverse events, severity should be recorded and grades as:

Table 8 - Adverse event severity grading

Grade	Severity	Comments
1	Mild	Aware of sign or symptom, but easily tolerated
2	Moderate	Discomfort enough to cause interference with usual activities
3	Severe	Incapacitating with inability to work or perform usual activities
4	Life-threatening	Participant is at immediate risk of death

A grade of severe should not be confused with a SAE. Severity refers to the intensity of an event and both AEs and SAEs can be severe. An event is defined as ‘serious’ when it meets one of the predefined outcomes as described in Section 9.3

The relationship to study product therapy should be assessed using the following definitions:

Table 9 – Adverse Event causality assessment

Causality	Comment
Unrelated	AE is clearly due to extraneous causes (e.g. underlying disease, environment, known effect of another drug)
Unlikely	The temporal association between the AE and study drug is such that study drug is not likely to have any reasonable association with the AE
Possible	The AE could have been produced by the subject’s clinical state or study drug
Probable	The AE follows a reasonable temporal sequence from the time of study drug administration, abates upon discontinuation of the study drug and cannot be reasonably explained by the

Causality	Comment
	known characteristics of the subject's clinical state
Definite	The AE follows a reasonable temporal sequence from the time of study drug administration, abates upon discontinuation of the study drug and/or reappears when study drug is re-introduced

These criteria in addition to good clinical judgment should be used as a guide for determining the causal assessment. If it is felt that the event is not related to study product, then an alternative explanation should be provided.

8.2.2 Adverse Event Reporting Period

All adverse events, regardless of severity, causality or seriousness must be reported from the date of informed consent until the end of the study or 28 days after the last dose of study medication, whichever is later. However, any adverse event that the Investigator believes is at least possibly related to study medication should be reported regardless of time elapsed from the final dose.

8.3 Serious Adverse Events

8.3.1 Serious Adverse Event Definition

A **serious adverse event** (SAE) is defined as follows:

Any adverse drug experience occurring at any dose that results in any of the following outcomes:

- death
- life-threatening situation (subject is at immediate risk of death)
- inpatient hospitalisation or prolongation of existing hospitalisation (excluding those for study therapy or placement of an indwelling catheter, unless associated with other serious events)
- persistent or significant disability/incapacity
- congenital anomaly/birth defect in the offspring of a subject who received study product
- Other: Important medical events that may not result in death, be immediately life-threatening, or require hospitalisation, may be considered a SAE when, based upon appropriate medical judgment, they may jeopardise the subject and may require medical or surgical intervention to prevent one of the outcomes listed in this definition. Examples of such events are:
    - intensive treatment in an emergency room or at home for allergic bronchospasm
    - blood dyscrasias or convulsions that do not result in hospitalisation
    - development of drug dependency or drug abuse

8.3.2 Clarification of Serious Adverse Events

Death is an outcome of an AE, and not an AE in itself. In reports of death due to "Disease Progression", where no other information is provided, the death will be assumed to have resulted from progression of the disease being treated with the study product(s).

All deaths, regardless of cause, must be reported to Sponsor for subjects on study and for deaths occurring within 30 days of last study product dose or within 30 days of last study evaluation, whichever is longer.

"Occurring at any dose" does not imply that the subject is receiving study product at the time of the event. Dosing may have been given as treatment cycles or interrupted temporarily prior to the onset of the SAE, but may have contributed to the event.

"Life-threatening" means that the subject was at immediate risk of death from the event as it occurred. This does not include an event that might have led to death, if it had occurred with greater severity.

Complications that occur during hospitalisations are AEs. If a complication prolongs hospitalisation, it is a SAE.

"In-patient hospitalisation" means the subject has been formally admitted to a hospital for medical reasons, for any length of time. This may or may not be overnight. It does not include presentation and care within an emergency department.

The Investigator should attempt to establish a diagnosis of the event based on signs, symptoms and/or other clinical information. In such cases, the diagnosis should be documented as the AE and/or SAE and not the individual signs/symptoms.

8.3.3 Serious adverse event reporting requirements

8.3.3.1 All SAEs

Admedus Vaccines Pty Ltd has requirements for expedited reporting of SAE's meeting specific requirements to worldwide regulatory authorities; therefore, all appropriate parties must be notified immediately regarding the occurrence of any SAE that occurs after the first dose of study product has been administered. The procedures for reporting all SAEs, regardless of causal relationship, are as follows:

- Complete the "Serious Adverse Event Report" Form:
    - Email the SAE report within 24 hours of the Investigator's knowledge of the event (Please refer to the Study Reference Manual for the designated email address);
    - For fatal or life-threatening events, also fax copies of hospital case reports, autopsy reports, and other documents when requested and applicable.

Admedus Vaccines Pty Ltd may request additional information from the Investigator to ensure the timely completion of accurate safety reports.

The Investigator must take all therapeutic measures necessary for resolution of the SAE. Any medications necessary for treatment of the SAE must be recorded onto the concurrent medication section of the subject's CRF.

8.3.3.2 Investigator reporting requirements for SAEs

An SAE may qualify for reporting to regulatory authorities if the SAE is considered to have a possible causal relationship to the study product, and is unexpected (Suspected Unexpected Serious Adverse Reaction [SUSAR]) based upon the current Investigator's Brochure. In this case for multi-centre studies, all Investigators will receive a formal notification describing the SAE.

Where this is required by local regulatory authorities, and in accordance with the local institutional policy, the Investigator should notify (in writing) the Human Research Ethics Committee (HREC) which approved the study of the SAEs, according to the HREC requirements as soon as is practical.

8.4 Follow up of Serious and Non-serious Adverse Events

Follow-up of serious and non-serious AEs will continue through the last day on study (including the follow-up, off study medication period of the study), until the Investigator and/or Admedus Vaccines Pty Ltd determine that the subject's condition is stable, or up to 30 days after the last dose of Study Product, whichever is longer. Admedus Vaccines Pty Ltd may request that certain AEs be followed until resolution.

8.5 Clinical Laboratory Abnormalities and Other Abnormal Assessments as AEs or SAEs

All laboratory values must be reviewed by the Investigator. Given that all laboratory data are collected and statistically analysed according to their respective toxicity gradings, laboratory abnormalities that occur without related clinical symptoms and signs should generally not be recorded as an AE unless they are considered clinically significant by the Investigator. Where possible, the overall diagnosis rather than the laboratory abnormality should be recorded on the CRF. This will avoid duplication of laboratory abnormalities in both the CRF and laboratory reports. Abnormal laboratory results that are of clinical significance should be reviewed by the Medical Monitor.

Any laboratory test result that meets the criteria for a SAE (refer to Section 9.3) should be recorded as an AE, the AE page of the CRF completed and a SAE form also completed in order for the Sponsor to collect additional information about that abnormality, including information regarding relationship to study product or other causes, any action taken and resolution.

8.6 Guidance for dose modification or discontinuation of treatment

To date, no specific toxicities have been identified that result from COR-1 treatment. Any toxicities and/or abnormal laboratory findings should be investigated for aetiology and graded according to Section 9.2 and Appendix 2.

The following toxicity management will be followed:

- Grade 1 or 2: Patients may continue study vaccination/placebo (except if they have an elevated temperature of >38 degrees Celsius or inter-current systemic illness). Dosing may be delayed for up to one week. Any dosing delays of > one week may require that the subject is withdrawn after discussion with the Medical Monitor. Safety assessments will occur immediately prior to each dosing of investigational vaccine/placebo.
- Grade 3: Patients with any Grade 3 toxicity considered to be at least possibly related to treatment (i.e. treatment related events) should be evaluated carefully by the Investigator prior to continuing study vaccination/placebo treatment. Investigators may discuss individual cases with the Medical Monitor.
- Grade 4: Patients developing any Grade 4 toxicity should have treatment interrupted. The Investigator should contact the Medical Monitor to discuss the subject's withdrawal.

Subjects will be withdrawn from treatment permanently should any of the following occur:

- Treatment related Grade 3 or 4 toxicity
- The need to take medication which could interfere with study measurements
- Subject unwilling to proceed and/or consent is withdrawn.

Where possible subjects who are withdrawn from treatment will remain on the study and attend the scheduled study visits, provided the Investigator deems it appropriate and the Sponsor approves.

Dosing will be stopped if suspected adverse drug reactions, changes to vital signs or clinical laboratory results are observed and these changes pose a significant health risk.

A corresponding adverse event will be captured within the CRF.

For any subject experiencing any event (irrespective of severity) which, in the opinion of the Investigator, contraindicates further dosing in that subject and the event is considered to be at least possibly related to treatment (or where causality to study treatment cannot be ruled out – see Section 9.2.1), continued dosing of the subject should be interrupted. In all cases, the final treatment decisions, made in response to toxicity, are the responsibility of the Investigator. A careful evaluation of the potential risk/benefit will dictate the optimal therapeutic course. Should treatment be interrupted, re-

initiation will follow review of the available safety data and in agreement with the Sponsor. There will be no dose reduction or modification.

If the Investigator deems that management of the patient's medical condition requires knowledge of the study treatment regimen, then the Medical Monitor must be contacted for consultation and the procedures described in Section 7.3 followed. If a patient dies from an event that is considered to be at least possibly related to treatment, continued dosing of all study participants should be interrupted.

Clinically significant suspected adverse drug reactions, and serious adverse events considered to be related to study procedures will be followed until resolved or considered stable. All subjects who experience a study drug related AE should be followed until resolution of the AE, even if the subject has discontinued study drug.

If an unscheduled interruption of study drug occurs, the study site should notify the Sponsor at the earliest possible time. In the event that a subject requires an unscheduled interruption of study drug under conditions other than those associated with toxicity, the case will be reviewed by the Sponsor to determine whether such a subject will be allowed to resume study drug.

Subjects withdrawn from study drug will be treated as deemed appropriate by the Investigator. Follow- up procedures should be performed and the appropriate CRFs should be completed.

The study may be terminated at any point in time at the discretion of the Sponsor.

8.7 Warnings and Precautions

The adverse event profile in humans of COR-1 has not been fully characterized.
In a Phase I clinical trial it was well tolerated. The most common adverse reactions expected include injection site reactions, and systemic adverse reactions including headache, fatigue, chills, malaise, pain and pyrexia.

For further information regarding warnings and precautions with the study drug, please refer to the Investigator's Brochure for COR-1.

8.8 Restrictions

Since all subjects must cease taking anti-viral therapy for at least 7 days prior to Visit 2 and throughout the study, anti-viral therapy will only be permitted if the Investigator believes that it is in the subject's best interest to commence this and only after Sponsor written approval. Antiviral therapy may not be commenced within 7 days prior to the shedding assessments and during the shedding assessments.

No other treatments for genital herpes should be taken by the subject during their participation on this study.

Vaccines are prohibited in this study.

For information regarding precautions and AEs with the investigational drug, the Investigator is referred to the Investigator's Brochure for COR-1.

8.9 Risks for Women of Childbearing Potential or during Pregnancy

The risks of treatment with Study Product during pregnancy have not been evaluated. Pre-menopausal women of childbearing potential will follow a medically prescribed birth control regimen or agree to abstain from heterosexual intercourse while participating in the study and for 28 days following the last dose of Study Product.

8.10 Procedures to be Followed in the Event of Pregnancy

The subject must be instructed to inform the Investigator IMMEDIATELY if she becomes pregnant during the study. No further vaccinations will be administered. The Investigator should report all pregnancies to Admedus Vaccines Pty Ltd or the Contract Research Organisation (CRO) acting on their behalf within 24 hours of becoming aware of the pregnancy. Pregnancies should be reported using the form in the CRF for reporting the occurrence and outcome of pregnancies in subject enrolled in the study.

Monitoring of the subject should continue until conclusion of the pregnancy. The outcome of the pregnancy should be reported to Admedus Vaccines Pty Ltd or CRO if the study is still in progress at the end of the pregnancy. If the end of the pregnancy occurs after the study has been completed, the outcome should be reported to Admedus Vaccines Pty Ltd.

8.11 Procedures to be followed in the Event of Symptomatic Genital Herpes (Outbreak)

Subjects will be educated at Visits 2, 3, 4, 11 and 16 about what an HSV-2 outbreak is and how to manage these during the study. This education may be repeated as deemed necessary by the Investigator or designee. At Visit 2, subjects will be provided with several copies of the *HSV-2 Outbreak Form*. The purpose of this form is to remind subjects of what they are required to do in the event of an HSV-2 Outbreak as well as to capture information about the event(s).

An HSV-2 Outbreak is defined as the occurrence of a papule, vesicle, pustule, ulcer or crust which is consistent with genital herpes (Wald et al. 2014).

On developing a lesion subjects will be informed to contact the clinic within 24 hours to arrange an appointment with the Investigator or designee so that lesion can be inspected and an assessment can be made as to whether it is consistent with genital HSV-2. The Investigator or designee will take a swab of the lesion and send this to the central laboratory.

If the subject notices the lesion on a weekend or public holiday, the following procedure is to be followed:

- The subject will contact the clinic as soon as the clinic reopens.
- A swab will be collected immediately upon the subject noticing the lesion using the 'HSV-2 Outbreak' collection pack which will be supplied to them at Visit 2. . The subject will carefully obtain as much sample as possible on the swab paying particular care not to spread the infection to other areas of their body.
- The subject will commence completing the *HSV-2 Outbreak Form* which will also be provided to them at Visit 2.

The subject will attend the clinic as soon as possible so the Investigator or designee can inspect the lesion and assess whether it is consistent with a genital HSV-2. The Investigator or designee will review the HSV-2 Outbreak Form and check that this is being completed correctly. If required re-education will be performed.

Swabs from the lesion(s) will be collected daily by the subject until the lesion(s) has healed. If more than one lesion is present, only one swab is required to be collected by using the same swab which is rubbed over each lesion. The subject will carefully obtain as much sample as possible. The subject will post their samples to the central laboratory.

There is no requirement for papules, vesicles or pustules to be broken. Crusts also should not be removed. Swabs will be sent to the central laboratory for analysis at least weekly. Subjects will be informed that they should avoid sexual contact until the lesion(s) has healed.

Site staff will enquire at each visit as to whether an outbreak(s) has occurred and collect corresponding completed *HSV-2 Outbreak Diary* which will be filed with the source notes.

Subjects will be reminded to avoid sexual contact in the event of developing a lesion.

9 *SAFETY REVIEW COMMITTEE*

Admedus Vaccines Pty Ltd have convened a Safety Review Committee (SRC) charged with the review of safety data and dosing decisions during the conduct of the study.

The SRC will meet regularly throughout the trial. Of particular importance will be a full review of all available safety data prior to the first subject receiving a booster injection. At this time, all available data will be reviewed by the SRC and agreement to proceed formalised in writing.

Adhoc meetings will be scheduled immediately should any safety concerns arise at any time during the conduct of the study.

The treatment blind will not be broken for the purposes of SRC review.

Meetings will be convened to review aggregate and individual patient level data by injection regimen prepared as listings and tables. At a minimum, baseline patient characteristics, vital signs and adverse events will be provided.

Membership of the SRC will include the Investigator(s), Medical Monitor for the study and one company representative. An expert advisor on vaccines immunology and/or infectious disease will be invited, as appropriate. The Clinical Project Manager or other Sponsor designated staff will be invited to facilitate information dissemination and preparation of documentation of meeting outcomes and decisions.

Details of the membership function and decision making processes of the SRC for this study are set out in a separate SRC Charter document.

10 SUBJECT COMPLETION/WITHDRAWAL

10.1 Subject Completion

A subject will be deemed to have completed the study once all trial procedures have been conducted. Any AEs or SAEs still ongoing at the time of the Exit Evaluation will be followed in accordance with Section 8.

10.2 Criteria for Premature Withdrawal from Treatment or the Study

Subjects have the right to withdraw from treatment or the study at any time for any reason. The Investigator must make every reasonable effort to keep each subject in the study except where termination or withdrawal is for reasons of safety. The Investigator also has the right to withdraw subjects from treatment or the study in the event of concurrent illness, AEs, pregnancy, treatment failure after a prescribed procedure, protocol violations, administrative reasons or other reasons.

It is understood by all concerned that an excessive rate of withdrawals from the study can render the study difficult to interpret. In particular, missing data on virological or immunological assessments could significantly impact on the interpretation of the results; therefore, unnecessary withdrawal of subjects from the study should be avoided.

The reasons for withdrawal of the subject must be recorded on the CRF. The following are considered justifiable reasons for subject withdrawal:

- the need to take medication which may interfere with study measurements
- intolerable/unacceptable adverse experiences
- major violation or deviation of study protocol
- non-compliance of subject with protocol
- subject unwilling to proceed and/or consent is withdrawn
- withdrawal from the study is, in the Investigator’s judgement, in the subject’s best interest
- pregnancy of female study subject at any time during the study period (if applicable).

10.3 Withdrawal of subjects from Study Product

Section 8.6 provides guidance for dose modification or discontinuation of Study Product in the event of AEs or abnormal laboratory values.

If a subject permanently discontinues dosing with Study Product, for example as a result of an AE, every attempt should be made to keep the subject in the study and continue to perform the required study related follow-up and procedures.
The ideal is to continue to follow the subject for the full study period or until resolution of the adverse event. If it is not possible for the subject to remain in the study for the full study period, every attempt should be made to keep the subject in the study up to the Exit Evaluation at Day/Week . If the subject remains in the study but off study treatment, all study procedures should continue as per protocol.

If the subject will not remain in the study for all study related procedures, the Exit Evaluation should be performed wherever possible.

All subjects who discontinue study medication dosing should be followed for at least 30 days after the last dose of Study Product in order to monitor subjects for possible post-treatment events which may occur after Study Product has been discontinued.

10.4 Withdrawal of Subjects from the Study

Should a subject decide to withdraw from the study, all efforts will be made to complete and report the observations as thoroughly as possible.

The Investigator should contact the subject either by telephone or through a personal visit, or a responsible relative must be contacted to determine, if possible, the reason for withdrawal. A complete final evaluation at the time of the subject withdrawal should be made with an explanation of why the subject is withdrawing from the study.

If the reason for removal of a subject from the study is an AE or an abnormal laboratory test result, the principal reason will also be recorded on the CRF. Where possible, subjects should be followed until the AE is resolved or the abnormal laboratory test has returned to normal.

10.5 Premature termination of the study

Admedus Vaccines Pty Ltd reserves the right to terminate the study at any time. Reasons will be provided in the event of this happening.

11 STATISTICAL ANALYSIS

The study is a randomized, double-blind, placebo controlled, Phase I/IIa trial designed as a pilot study whose primary objective is to describe the safety and tolerability of the HSV-2 DNA vaccine COR-1. The study design also includes 2 injection regimen cohorts of: 1) two injections, 500 mcg each, into the same arm, or 2) two injections, 500 mcg each, administered as one injection per arm

Within each injection regimen cohort, subjects will be randomized to receive COR-1 or placebo using a 3:1 allocation ratio. Within each injection regimen, randomization to COR-1 or placebo will be double-blinded. Given the pilot and exploratory nature of the study, the focus of the statistical analysis will be descriptive rather than hypotheses testing. If inferential statistical tests of hypotheses are employed, they will be viewed as exploratory and descriptive rather than confirmatory.

An interim analysis of unblinded immunogenicity and efficacy data is planned. In summary the following available analyses will be performed:

1. Immunogenicity data (ELISPOT and ELISA); and
2. Viral shedding data.

For the analyses, descriptive statistics will be summarized by injection regimen as well as overall. Additional data summaries may include those for a pooled placebo group and/or a pooled COR-1 group. Prior to pooling and to aid in the interpretation, the individual groups contributing to the pooled group will be reviewed to determine how similar or disparate the groups are.
The following provides a general overview of the statistical methods. The Statistical Analysis Plan (SAP), will be written and finalized prior to the study blind break and data base lock for both the interim and final analyses.

11.1 Sample Size Determination

Since the current trial is a pilot study, the sample size was determined on the basis of practical and logistical considerations and not based on statistical power with regard to hypothesis testing or precision with regard to parameter estimation.

11.1.1 Analysis Sets

The following defines the analytic sample(s) for the relevant endpoint used in a particular analysis.

Full Analysis Set (FAS): The full analysis set will include all subjects randomized and any , who receive study drug and have at least one post-baseline measurement for the relevant endpoint being described. For endpoints requiring a change from, or comparison to, baseline, a baseline measurement will also be required. Subjects will be analysed according to their randomized treatment group regardless of the actual treatment received. Further details will be provided in the SAP.

Per Protocol Analysis Set: The per-protocol analysis set will consist of all randomized subjects receiving study drug who are compliant with inclusion/exclusion criteria, study drug administration, study procedures, and measurement follow-up. Further details including the criteria for compliance will be detailed in the SAP.

Intent-to-Treat Set (ITT): The ITT analysis set will include all randomized subjects. Subjects will be analysed according to their randomized treatment group regardless of exposure to study drug or the actual treatment received.

Safety Analysis Set: The safety analysis set will include all subjects who receive at least one injection of study drug. Subjects will be included in the treatment group according to the actual treatment received regardless of their randomized assignment.

11.1.2 Subject Disposition, Demographics and Baseline Characteristics

Descriptive statistics with regard to subject disposition using the ITT analysis set will be provided by randomized dose/injection regimen. If feasible, a Kaplan-Meier plot of time-to-study withdrawal will also be estimated.

Tables of descriptive statistics for subject demographics and baseline characteristics will also be provided.

11.1.3 Safety Evaluations

Safety endpoints to be examined include:

- Adverse events
- Clinical laboratory measurements
- Immunogenicity
- Vital Signs

The analysis of safety will be descriptive, and unless otherwise stated, all safety analyses will use the Safety Analysis set.

11.1.4 Adverse Events

The primary analysis of adverse events will consider only treatment-emergent AEs (TEAEs), events occurring for the first time, or worsening, during or after the first administration of study drug. The analysis will focus on subject incidence, although for TEAEs of special interest, the number of events may also be provided. Tables summarizing TEAEs will be displayed by preferred term and system organ class (SOC). The analysis will also include the analysis of serious adverse events (SAEs) and the categorization of AEs by severity and relationship to study drug. The analysis of AEs leading to study withdrawal or deaths on-study will also be provided in tables and/or listings. Further details will be provided in the SAP.

11.1.5 Vital Signs, Laboratory Parameters, and Immunogenicity

The analysis of vital signs and laboratory parameters will include tabulations by dose/injection regimen. Descriptive statistics for each parameter and/or the change from baseline at each time point will be provided. For endpoints measured on a categorical scale the number of subjects and percents will be provided. Further details will be provided in the SAP.

11.1.6 Exploratory Efficacy Evaluations

The analysis of exploratory efficacy endpoints will be descriptive providing summary statistics reflecting the endpoint at each time point and/or change or shift from baseline as appropriate. Efficacy analyses will be based on the FAS and/or Per Protocol Analysis Set. Data listings will also be provided. Further details will be provided in the SAP.

11.1.7 Interim Analysis

Blinded safety data will be periodically reviewed by the Safety Review Committee, details of which are provided in a separate Charter.

An interim analysis of unblinded immunogenicity and efficacy data is planned. In summary, the following available analyses will be performed:

- Immunogenicity data (ELISPOT and ELISA); and
- Viral shedding data.

12 GENERAL STUDY ADMINISTRATION

12.1 Ethical Aspects

Subjects who are enrolled into this study that can be verified as having received placebo may be eligible to participate in further clinical trials involving COR-1.

12.1.1 Local regulations/Declaration of Helsinki

The Investigator will ensure that this study is conducted in full conformance with the protocol, the latest version of the "Declaration of Helsinki (and its amendments and with the requirements of national drug and data protection laws of the countries in which the research is conducted).

In other countries, Admedus Vaccines Pty Ltd and the Investigators will ensure strict adherence to the provisions of Good Clinical Practice and all applicable and national regulations. The International Conference on Harmonisation (ICH) guidelines will apply as a minimum.

The Sponsor agrees to abide by the Medicines Australia Guidelines for the 'Compensation for Injury Resulting from Participation in a Company-Sponsored Clinical Trial' (16 January 2004). Compensation will only be provided on the understanding that the provision of compensation does not amount to an admission of legal liability, and is subject to the proposed recipient signing a full and complete release of Admedus Vaccines Pty Ltd from all claims, damages and costs.

12.1.2 Informed consent

It is the responsibility of the Investigator to obtain written informed consent from each individual participating in this study after adequate explanation of the aims, methods, objectives and potential hazards of the study prior to undertaking any study related procedures. The Investigator must also explain to the subject that they are completely free to refuse to enter the study or to withdraw from it at any time for any reason. The Investigator must utilise an HREC approved consent form for documenting written informed consent.

12.1.3 Premature withdrawal

If subjects discontinue study medication dosing, for example as a result of an AE, every attempt should be made to keep the subject in the study and continue to perform the required study related follow-up and procedures. If this is not possible or acceptable to the subject or Investigator, the subject may be withdrawn from the study. Refer to Section 10 for more detail.

12.1.4 Institutional Review Boards or Ethics Committees

This protocol and any accompanying material provided to the subject (such as subject information sheets or descriptions of the study used to obtain informed consent), will be submitted, by the Investigator, to an HREC. Approval from the committee must be obtained before starting the study, and should be documented in a letter to the Investigator specifying the protocol number and version and the date on which the committee met and granted the approval.

Any modifications made to the protocol after receipt of HREC approval must also be submitted by the Investigator to the committee in accordance with institutional procedures and regulatory requirements.

12.1.5 Conditions for modifying the protocol

Protocol modifications to ongoing studies which could potentially adversely affect the safety of participating subjects or which alter the scope of the investigation, the scientific quality of the study, the experimental design, dosages, duration of therapy, assessment variables, the number of subjects

treated or subject selection criteria, may be made only after consultation between an appropriate representative of the Sponsor and the Investigator.

Protocol modifications (amendments) must be prepared by a representative of the Sponsor and initially reviewed and approved by the responsible Medical Monitor and (when applicable) the Statistician.

All protocol modifications must be submitted to the HREC in accordance with local requirements. Approval must be awaited before changes can be implemented.

In the event of an emergency, the Investigator may institute any medical procedures deemed appropriate. However, all such procedures must be promptly reported to Admedus Vaccines Pty Ltd, the Medical Monitor and the HREC.

Administrative changes of the protocol are defined as minor corrections and/or clarifications that have no effect on the way the study is to be conducted, or on the safety of the subjects. These administrative changes will be agreed upon by Admedus Vaccines Pty Ltd and the Investigator, and will be documented in a memorandum. The Investigator will then notify the HREC of such administrative changes.

12.1.6 Conditions for terminating the study

Both the Sponsor and the Investigator reserve the right to terminate the study at any time. Should this be necessary, the procedures will be arranged on an individual study basis after review and consultation by both parties. In terminating the study, Admedus Vaccines Pty Ltd and the Investigator will assure that adequate consideration is given to the protection of the subject’s interests.

12.2 Study Documentation, CRFs and Record Keeping

12.2.1 Investigator’s files/Retention of documents

The Investigator must maintain adequate and accurate records to enable the conduct of the study to be fully documented and the study data to be subsequently verified.

These documents should be classified into two separate categories:

a. Investigator’s Site File, and
b. Subject clinical source documents.

The Investigator’s Site File will contain essential documents including the protocol/amendments, HREC approval with correspondence, informed consent, drug records, staff curriculum vitae and authorisation forms and other appropriate documents and correspondence.

Subject clinical source documents include subject hospital/clinic records, physician’s and nurse’s notes, appointment book, original laboratory reports, ECG, EEG, X-ray, pathology and special assessment reports, consultant letters, screening and enrolment log, etc. All electronic records must be FDA 21 CFR part 11 compliant. All clinical study documents must be retained by the Investigator until at least 2 years after the last approval of a marketing application in an International Conference on Harmonization (ICH) region (i.e. USA, Europe, or Japan) and until there are no pending or contemplated marketing applications in an ICH region; or if no application is filed or if the application is not approved for such indication, until 2 years after the investigation is discontinued and regulatory authorities have been notified. The Investigator must notify Admedus Vaccines Pty Ltd prior to destroying any clinical study records.

Should the Investigator wish to assign the study records to another party or move them to another location, Admedus Vaccines Pty Ltd must be notified in advance.

If the Investigator cannot guarantee this archiving requirement at the study site for any or all of the documents, special arrangements must be made between the Investigator and Admedus Vaccines Pty

Ltd to store these in a sealed container(s) outside of the site so that they can be returned sealed to the Investigator in case of a regulatory audit. Where source documents are required for the continued care of the subject, appropriate copies should be made for storage outside of the site.

12.2.2 Background data

The Investigator shall supply the Admedus Vaccines Pty Ltd, on request, with any required background data from the study documentation or clinic records. This is particularly important when errors in data transcription are suspected. In case of special problems and/or governmental queries or requests for audit inspections, it is also necessary to have access to the complete study records, provided that subject confidentiality is protected.

12.2.3 Audits and Inspections

The Investigator should understand that source documents for this trial should be made available to appropriately qualified personnel from Admedus Vaccines Pty Ltd or its representative or to regulatory authority or health authority inspectors after appropriate notification. The verification of the CRF data may be by direct inspection of source documents (where permitted by law) or through an interview technique.

12.2.4 Case Report Forms

For each subject enrolled, CRFs must be completed and signed or electronically signed by the Investigator or his/her authorised delegate. This also applies to records for those subjects who fail to complete the study (even during a pre-randomisation screening period). If a subject withdraws from the study, the reason must be noted on the CRF. If a subject is withdrawn from the study because of a treatment-limiting AE, thorough efforts should be made to clearly document the outcome.

All hardcopy forms should be typed or filled out using a black/blue pen, and must be legible. Errors should be crossed out but not obliterated, the correction inserted, and the change initialled and dated by the Investigator or his/her authorised delegate. The CRFs, as well as the protocol, are confidential. The CRFs remain the property of the Sponsor at all times.

12.3 Monitoring the Study

In accordance with International Conference on Harmonization Good Clinical Practice (ICH-GCP) guidelines, the study monitor must have direct access to the Investigator’s source documentation in order to verify the data recorded in the CRFs for consistency.

It is understood that the responsible monitor, as an Admedus Vaccines Pty Ltd representative, will contact and visit the Investigator regularly and that he/she will be allowed, on request, to inspect the various records of the trial (CRFs and other pertinent data) provided that subject confidentiality is maintained in accord with local requirements.

It will be the monitor’s responsibility to inspect the CRFs at regular intervals throughout the study, to verify the adherence to the protocol and the completeness, consistency and accuracy of the data being entered on them. Where local regulations permit, the monitor should have access to laboratory test reports and other subject records needed to verify the entries on the CRF. The Investigator agrees to cooperate with the monitor to ensure that any problems detected in the course of these monitoring visits are resolved.

12.4 Confidentiality of Trial Documents and Subject Records

The Investigator must assure the subjects’ anonymity will be maintained and that their identities are protected from unauthorised parties. On all study documents submitted to the Sponsor, subjects should not be identified by their names, but by the subject’s initials and an identification code. The Investigator should keep a subject enrolment log showing codes, names and contact details.

Documents not for submission to Admedus Vaccines Pty Ltd (e.g. subject's written consent forms), should be maintained by the Investigator in strict confidence.

All information concerning the study treatment and Admedus Vaccines Pty Ltd and its operation, such as patent applications, formulae, manufacturing processes, basic scientific data and material not previously published are considered confidential and shall remain the sole property of the Sponsor. The Investigator agrees to use this information only in accomplishing the study and will not use it for any other purposes without written consent from the Sponsor.

12.5 Publication of Data and Protection of Trade Secrets

In accord with standard editorial and ethical practice, Admedus Vaccines Pty Ltd will support publication of multicentre trials only in their entirety and not as individual centre data.

Admedus Vaccines Pty Ltd will list the study on a public database listing of clinical trials, for example, Clinical trials.gov and anzctr.org.au.

The results of this study may be published or presented at scientific meetings. If this is envisaged, the Investigator agrees to submit all manuscripts or abstracts to Admedus Vaccines Pty Ltd prior to submission. This allows Admedus Vaccines Pty Ltd to protect proprietary information and to provide comments based on information from other studies that may not yet be available to the Investigator.

Any formal publication of the study in which input of Admedus Vaccines Pty Ltd personnel exceeded that of conventional monitoring will be considered as a joint publication by the Investigator and the appropriate Admedus Vaccines Pty Ltd personnel. Authorship will be determined by mutual agreement prior to the start of the study. Number of subjects enrolled will be taken into consideration in the selection of authors. Additional authors will be agreed prior to the completion of the study.

12.6 Anticipated Subject Accrual and Duration of the Study

The anticipated patient accrual will be 40 patients in 6 months. The Investigator should continually compare the actual and expected accrual rates, and make every effort to ensure that they are as closely matched as possible. If the Investigator anticipates major problems with recruitment, or delay in the expected completion date, he/she should discuss this with the Admedus Vaccines Pty Ltd. as early as possible.

13 REFERENCES

Abu-Raddad LJ, Magaret AS, Celum C, Wald A, Longini IM Jr, Self SG, Corey L (2008). Genital herpes has played a more important role than any other sexually transmitted infection in driving HIV prevalence in Africa. PLoS One; 3(5): e2230

Bode C, Zhao G, Steinhagen f, Kinjo T, Klinman DM. CpG DNA as a vaccine adjuvant. *Expert Review of Vaccines* 2011 10:4, 499-511

Corey L, Langenberg AGM, Ashley R, Sckulovich RE, Izu AE, Douglas JM, Handsfield HH, Warren T, Marr L, Tyring S, DiCarlo R, Adimora AA, Leone P, Dekker CL, Burke RL, Leong WP, Straus SE (1999). Recombinant Glycoprotein Vaccine for the Prevention of Genital HSV-2 Infection- Two Randomized Controlled Trials. JAMA. Vol .281; 4; 331-340

FDA (2007a). Guidance for Industry – Considerations for Plasmid DNA Vaccines for Infectious Disease Indications. November 2007. http://www.fda.gov/downloads/BiologicsBloodVaccines/GuidanceComplianceRegulatoryInformation/Guidances/Vaccines/ucm091968.pdf (accessed 1st July 2014).

FDA (2007b). Guidance for Industry – Toxicity Grading Scale for Healthy Adult and Adolescent Volunteers Enrolled in Preventive Vaccine Clinical Trials. September 2007. http://www.fda.gov/downloads/BiologicsBloodVaccines/GuidanceComplianceRegulatoryInformation/Guidances/Vaccines/ucm091977.pdf (accessed 1st July 2014).

Ferraro B, Morrow MP, Nutnick NA, Shin TH, Lucke C Weiner DB (2011). Clinical applications of DNA vaccines: current progress. Clin Infect Dis. (2011) 53 (3): 296-302. doi:10.1093/cid/cir334

Haddow LJ, Dave B, Mindel A, McPhie KA, Chung C, Marks C, Dwyer DE (2006). Increase in rates of herpes simplex virus type 1 as a cause of anogenital herpes in western Sydney, Australia, between 1979 and 2003. Sex Transm Infect; 82(3): 255-259.

Gupta R, Wald A, Krantz E, Selke S, Warren T, Vergas-Cortes M, Miller G, Corey L (2004) Valcyclovir and Acyclovir and Acyclovir for Suppression of Shedding of Herpes Simplex Virus in the Genital Tract. JID 1990:1374-1381

Johnston C, Koelle D, Wald A (2011). HSV-2 in pursuit of a vaccine. J Clin Invest. 2011;121(12):4600–4609. doi:10.1172/JCI57148.

Kimberlin D (2007). Herpes Simplex Virus Infections of the Newborn. Seminars in Peinatology, Volum 31, Issue 1, Pages 19-25. DOI: 10.1053/j.semperi.2007.01.003

Klinman DM, Klaschick S, Tross D, et al. FDA guidance on prophylactic DNA vaccines: analysis and recommendations. *Vaccine* 2010;28(16): 2801-05.

Magaret AS, Stanaway J (2011). Sample Size for a Binomial Proportion with Autcorrelation. Sat Commun. Infect Dis. Doi:10.2202/1948-4690.1036

Malkin JE, (2004). Epidemiology of genital herpes simplex virus infection in developed countries. Herpes. 2004 Apr;11 Suppl 1:2A-23A.

Manam S, Ledwith BJ, Barnum AB, et al. Plasmid DNA vaccines: tissue distribution and effects of DNA sequence, adjuvants and delivery method on integration in host DNA. *Intervirology* 2000;43(4-6):273-81.

Medicines Australia (2004). Guidelines for compensation for injury resulting from participation in a company sponsored clinical trial. http://medicinesaustralia.com.au/files/2010/09/Clnical-Trials-Compensation-Guidelines.pdf (accessed 1st July 2014).

Mertz G (2008). Asymptomatic Shedding of Herpes Simplex Virus 1 and 2: Implications for Prevention of Transmission. J Infect Dis. (2008) 198 (8): 1098-1100.
doi: 10.1086/591914

NHMRC (1999). National Statement on Ethical Conduct in Research Involving Humans (1999). http://www.nhmrc.gov.au/_files_nhmrc/publications/attachments/e35.pdf (accessed 1st July 2014).

Norquist JM, Khawaja SS, Mast TC, Liaw KL, Robertson MN, Evans B, Gutsch D, Saddler P (2012) Adaption of a previously validated vaccination report card for use in adult vaccine clinical trials to align with the 2007 FDA Toxicity Grading Scale Guidance. Human Vaccines & Immunotherapeutics 8:10, 1208-1212.

PICS (2009). Guide to Good Manufacturing Practice for Medicinal Products – Annex 13 Manufacture of Investigational Medicinal Products. http://www.tga.gov.au/pdf/manuf-pics-gmp-medicines-annexes.pdf (accessed 1st July 2014).

Schiffer JT, Mayer BT, Hong Y, Swan DA, Wald A (2014). Herpes simplex virus-2 transmission probability estimates bassed on quantity of viral shedding. J. R. Soc. Interface June 6, 2014 11 95 20140160; doi:10.1098/rsif.2014.0160 1742-5662

TGA (2000). Note for guidance on good clinical practice (CPMP/ICH/135/95 - Annotated with TGA comments). http://www.tga.gov.au/pdf/euguide/ich13595.pdf (accessed 1st July 2014).

Tronstein E, Johnston C, Huang ML, Selke S, Magaret A, Warren T, Corey L, Wald A (2011). Genitial Shedding of Herpes Simplex Virus Among Symptomatic and Asymptomatic Person With HSV-2 Infection. JAMA Vol.305 No.14 1441-1449.

Wald A, Corey L, Timmler B, Magaret A, Warren T, Tyring S, Johnston C, Kriesel J, Fife K, Galitz L, Stoelben S, Huang ML, Selke S, Sobernack HP, Ruebsamen-Schaeff H, Birkmann A (2014). Helicase Primase Inhibitor Pritelivir for HSV-2 Infection. N. Eng J Med 307:3 201-210.

WHO (2001). Annex 1: WHO Guidelines on clinical evaluation of vaccines: regulatory expectations. URL: http://www.who.int/biologicals/publications/clinical_guidelines_ecbs_2001.pdf (accessed 30 June 2014).

WMA (2008). Declaration of Helsinki – Ethical Principles for Medical Researching Involving Human Subjects.http://www.wma.net/en/30publications/10policies/b3/index.html.pdf?print-media-type&footer- right=[page]/[toPage] (accessed 30 June 2014).

21 CFR Chapter 11. Food and Drugs, Chapter 1- Food and Drug Administration, Department of Health and Human Services, Subchapter A – general. Part 11 electronic records, electronic signatures. http://www.accessdata.fda.gov/scripts/cdrh/cfdocs/cfcfr/cfrsearch.cfm?cfrpart=11 (accessed 30 June 2014).

14 APPENDICES

14.1 APPENDIX 1: Procedure for Intradermal Injections

COR-1 is administered using a single-dose disposable 1 mL insulin (or tuberculin) syringe and a 27- gauge ½" needle (Terumo or equivalent). A volume of 0.2 mL will be administered intradermally.

Un-blinded investigational product administrators will be assigned for each site at the commencement of the study. These administrators will be authorised by the Investigator and will be listed on the delegation of duties log.

Equipment and materials:

1. 1 mL Terumo insulin (or tuberculin) syringe (27 Gauge ½" needle)
2. Alcohol swabs or equivalent
3. The emergency cart should be available in the immediate area
4. Sharps container
5. Detergent wipes
6. Gloves
8. Ruler to measure the diameter of the bleb after intradermal injection
9. Source documents/pen

Procedure:

1. The subject must be seated, not standing.
2. In order to maintain the blind, the subject must not be able to observe their forearm whilst it is being injected. A physical barrier for example a material screen may be utilized for this purpose or the subject may simply turn their head away until they are instructed that they may turn their head back.
3. Place arm in a relaxed position, elbow flexed. Palm side up.
4. Make sure that the area where vaccinations will be given has a firm, well-lit surface on to which to rest the subject's arm
5. Clean the skin at the injection site thoroughly with an alcohol swab or equivalent. Use a circular motion from the centre of the injection site outward. If the skin is dirty use detergent wipes and rinse with water.
6. Remove needle cap
7. Stretch the skin at the site to be injected between the thumb and forefinger.
8. Hold the syringe, bevel up, with fingers and thumb resting on the sides of the barrel. Slowly insert needle, bevel up, just under the skin at an angle of 5 to 15° until the bevel is covered and lies just under the skin (approximately 3 mm). When the needle is inserted at the correct angle you can see the bevel of the needle just below the skin surface. Release the stretched skin and hold the syringe in place.

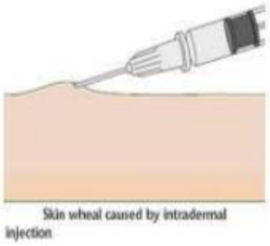

Figure1: Example of an intradermal injection

9. Grip the flange of the syringe between first and middle fingers. Use your thumb to press on the plunger. Slowly inject 0.2 mL of the vaccine. You should feel firm resistance as the liquid enters the skin. A tense, pale bleb or wheal (blister like) appears over the needle bevel.

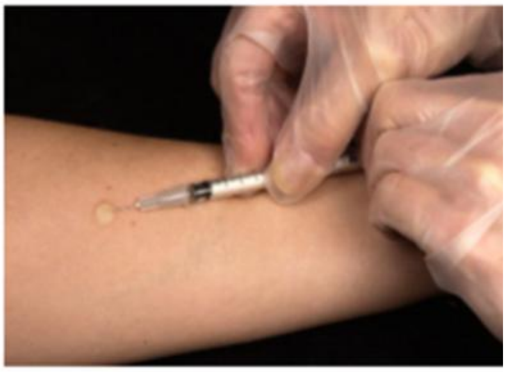

Figure 2: Example of an intradermal injection

10. If none appears immediately, resite the position of the needle.
11. If the bleb does not appear, the solution has been injected too deeply. Record in the source notes if an injection does not produce the bleb, or leaks following the injection within 15 minutes.
    Withdraw the needle quickly at the same angle it was inserted and dispose of according to universal precautions. The bleb should not be covered with a dressing or touched by the subject but left to heal open to the air.
12. Record the time and date of administration of the investigational vaccine in the source notes. Record the diameter of the 'bleb' immediately after the injection and also any other relevant observations.
13. If the subject has been randomised to receive two injections on the same arm, the second injection will be administered 5 cm away radially, not transversely from the first injection site using the procedure detailed above. Subject's in Group 2 may choose which arm will be injected, however where possible the non-dominant arm is preferred. Where possible the same arm will be injected throughout the course of the study
14. Subjects will be instructed to keep their forearm(s) extended for 10 – 15 minutes until the 'bleb' subsides.
15. The diameter of the bleb will again be measured after the period of time that the subject has kept their arm extended. This measurement will be recorded in the source notes.

14.2 APPENDIX 2: Guidance for Industry, Toxicity Grading Scale for Healthy Adult and Adolescent Volunteers Enrolled in Preventive Vaccine Clinical Trials, September 2007

Guidance for Industry

Toxicity Grading Scale for Healthy Adult and Adolescent Volunteers Enrolled in Preventive Vaccine Clinical Trials

Additional copies of this guidance are available from the Office of Communication, Training and Manufacturers Assistance (HFM-40), 1401 Rockville Pike, Suite 200N, Rockville, MD 20852-1448, or by calling 1-800-835-4709 or 301-827-1800, or from the Internet at http://www.fda.gov/cber/guidelines.htm.

For questions on the content of this guidance, contact the Division of Vaccines and Related Products Applications, Office of Vaccines Research and Review at 301-827-3070.

U.S. Department of Health and Human Services
Food and Drug Administration
Center for Biologics Evaluation and Research
September 2007

Contains Nonbinding Recommendations

Table of Contents

I. INTRODUCTION.. 1
II. BACKGROUND.. 2
III. TOXICITY GRADING SCALE TABLES.. 2
A. Tables for Clinical Abnormalities 3
B. Tables for Laboratory Abnormalities.. 6
IV. REFERENCES.. 8

Contains Nonbinding Recommendations

Guidance for Industry

Toxicity Grading Scale for Healthy Adult and Adolescent Volunteers Enrolled in Preventive Vaccine Clinical Trials

This guidance represents the Food and Drug Administration's (FDA's) current thinking on this topic. It does not create or confer any rights for or on any person and does not operate to bind FDA or the public. You can use an alternative approach if the approach satisfies the requirements of the applicable statutes and regulations. If you want to discuss an alternative approach, contact the appropriate FDA staff. If you cannot identify the appropriate FDA staff, call the appropriate number listed on the title page of this guidance.

I. INTRODUCTION

Preventive vaccines are usually developed to prevent disease in a healthy population. The Office of Vaccines Research and Review, Center for Biologics Evaluation and Research, regulates preventive vaccines under authority of section 351 of the Public Health Service Act (42 U.S.C. 262), as well as specific sections of the Federal Food, Drug, and Cosmetic Act, and reviews investigational new drug applications (INDs) and biologics license applications (BLAs). (See, for example, Title 21 Code of Federal Regulations (CFR) Parts 312, 600, and 601). Most of the clinical trials of preventive vaccines conducted to support INDs and BLAs enroll healthy volunteers in all phases of vaccine testing. The enrollment of healthy volunteers warrants a very low tolerance for risk in those clinical trials.

This guidance provides you, sponsors, monitors, and investigators of vaccine trials, with recommendations on assessing the severity of clinical and laboratory abnormalities in healthy adult and adolescent volunteers enrolled in clinical trials. The grading system described in the table can also be useful in defining a particular study's stopping rules (e.g., a certain number of adverse events, as defined in the table, may call for stopping the study). Less extreme observations (e.g., mild) may not require discontinuing the study vaccine but can still contribute to evaluating safety by identifying parameters to focus upon in subsequent product development. Uniform criteria for categorizing toxicities in healthy volunteers can improve comparisons of safety data among groups within the same study and also between different studies. We, FDA, recommend using toxicity grading scale tables, provided below, as a guideline for selecting the assessment criteria to be used in a clinical trial of a preventive vaccine. We recommend incorporation of such appropriate, uniform, criteria into the investigational plan, case report forms, and study reports and correspondence with FDA, sponsors, monitors, investigators, and IRBs.

This guidance finalizes the draft guidance of the same title dated April 2005 (70 FR 22664, May 2, 2005).

Contains Nonbinding Recommendations

FDA's guidance documents, including this guidance, do not establish legally enforceable responsibilities. Instead, guidances describe FDA's current thinking on a topic and should be viewed only as recommendations, unless specific regulatory or statutory requirements are cited. The use of the word *should* in FDA's guidances means that something is suggested or recommended, but not required.

II. BACKGROUND

Standardized toxicity assessment scales have been widely used to evaluate products treating specific diseases. For example, the National Cancer Institute's Common Toxicity Criteria Scale and the Division of AIDS' Toxicity Grading Scale standardize the evaluation of adverse events among patients with cancer and HIV/AIDS, respectively (Refs. 1, 2). The defined toxicity parameters in those scales are designed for patients who may already experience mild, moderate, or severe adverse clinical or laboratory events due to the disease process, and may not be appropriate for healthy volunteers.

In the development of the toxicity grading scales for healthy volunteers, we chose parameter limit values based on published information, when such values were available (Refs. 1-6). For example, the Brighton Collaboration has developed case definitions and guidelines to evaluate some adverse events associated with administering vaccines (Ref. 3). In some cases, parameter limit values were based on clinical experience and experience reviewing vaccine clinical trials that enroll normal healthy subjects.

Toxicity grading scales for laboratory abnormalities should consider the local laboratory reference values when the parameter limit values are defined. The characterization of laboratory parameters among some populations of healthy adults and adolescents may require the exercise of clinical judgment, for example, consideration of the potential for ethnic differences in white blood cell (WBC) counts or gender differences in creatine phosphokinase (CPK) values.

III. TOXICITY GRADING SCALE TABLES

Adverse events in a clinical trial of an investigational vaccine must be recorded and monitored and, when appropriate, reported to FDA and others involved in an investigation (sponsors, IRBs, and investigators). (See, for example, 21 CFR 312.32, 312.33, 312.50, 312.55, 312.56, 312.60, 312.62, 312.64, 312.66). Although the use of a toxicity grading scale for adverse events would not replace these regulatory requirements, using a scale to categorize adverse events observed during a clinical trial may assist you in monitoring safety and making required reports. Nonetheless, we believe that categorization or grading of data as outlined in this document is supplementary to and should not replace full and complete data analysis.

These guidelines for toxicity grading scales are primarily intended for healthy adult and adolescent volunteers. The parameters in the tables below are not necessarily applicable to every clinical trial of healthy volunteers. The parameters monitored should be appropriate for the specific study vaccine. For some preventive vaccines under development, it may be appropriate

Contains Nonbinding Recommendations

to include additional parameters to be monitored during a clinical trial or to alter the choice of values in the toxicity table. For example, additional parameters might be added based on one or more of the following: safety signals observed in pre-clinical toxicology studies, the biological plausibility of the occurrence of certain adverse events, or previous experience with a similar licensed product.

As discussed above, the tables do not represent a recommendation to monitor all the listed parameters in all clinical trials of healthy volunteers, nor do the tables represent all possible parameters to be monitored. In addition, these tables do not represent study inclusion or exclusion criteria. We recommend that the parameters monitored be appropriate for the study vaccine administered to healthy volunteers participating in the clinical trial.

A. Tables for Clinical Abnormalities

Local Reaction to Injectable Product	Mild (Grade 1)	Moderate(Grade 2)	Severe (Grade 3)	Potentially Life Threatening (Grade 4)
Pain	Does not interfere with activity	Repeated use of non-narcotic pain reliever > 24 hours or interferes with activity	Any use of narcotic pain reliever or prevents daily activity	Emergency room (ER) visit or hospitalization
Tenderness	Mild discomfort to touch	Discomfort with movement	Significant discomfort at rest	ER visit or hospitalization
Erythema/Redness *	2.5 – 5 cm	5.1 – 10 cm	> 10 cm	Necrosis or exfoliative dermatitis
Induration/Swelling **	2.5 – 5 cm and does not interfere with activity	5.1 – 10 cm or interferes with activity	> 10 cm or prevents daily activity	Necrosis

* In addition to grading the measured local reaction at the greatest single diameter, the measurement should be recorded as a continuous variable.

** Induration/Swelling should be evaluated and graded using the functional scale as well as the actual measurement.

3

Contains Nonbinding Recommendations

Vital Signs *	Mild (Grade 1)	Moderate(Grade 2)	Severe (Grade 3)	Potentially Life Threatening (Grade 4)
Fever (°C) ** (°F) **	38.0 – 38.4<br>100.4 – 101.1	38.5 – 38.9<br>101.2 – 102.0	39.0 – 40<br>102.1 – 104	> 40<br>> 104
Tachycardia - beats per minute	101 – 115	116 – 130	> 130	ER visit or hospitalization for arrhythmia
Bradycardia - beats per minute***	50 – 54	45 – 49	< 45	ER visit or hospitalization for arrhythmia
Hypertension (systolic) - mm Hg	141 – 150	151 – 155	> 155	ER visit or hospitalization for malignant hypertension
Hypertension (diastolic) - mm Hg	91 – 95	96 – 100	> 100	ER visit or hospitalization for malignant hypertension
Hypotension (systolic) – mm Hg	85 – 89	80 – 84	< 80	ER visit or hospitalization for hypotensive shock
Respiratory Rate – breaths per minute	17 – 20	21 – 25	> 25	Intubation

* Subject should be at rest for all vital sign measurements.
** Oral temperature; no recent hot or cold beverages or smoking.
*** When resting heart rate is between 60 – 100 beats per minute. Use clinical judgement when characterizing bradycardia among some healthy subject populations, for example, conditioned athletes.

Systemic (General)	Mild (Grade 1)	Moderate(Grade 2)	Severe (Grade 3)	Potentially Life Threatening (Grade 4)
Nausea/vomiting	No interference with activity or 1 – 2 episodes/24 hours	Some interference with activity or > 2 episodes/24 hours	Prevents daily activity, requires outpatient IV hydration	ER visit or hospitalization for hypotensive shock
Diarrhea	2 – 3 loose stools or < 400 gms/24 hours	4 – 5 stools or 400 – 800 gms/24 hours	6 or more watery stools or > 800gms/24 hours or requires outpatient IV hydration	ER visit or hospitalization
Headache	No interference with activity	Repeated use of non-narcotic pain reliever > 24 hours or some interference with activity	Significant; any use of narcotic pain reliever or prevents daily activity	ER visit or hospitalization
Fatigue	No interference with activity	Some interference with activity	Significant; prevents daily activity	ER visit or hospitalization
Myalgia	No interference with activity	Some interference with activity	Significant; prevents daily activity	ER visit or hospitalization

4

Contains Nonbinding Recommendations

**Systemic Illnes**	**Mild (Grade 1)**	**(Moderate(Grade 2)**	**Severe (Grade 3)**	**Potentially Life Threatening (Grade 4)**
Illness or clinical adverse event (as defined according to applicable regulations)	No interference with activity	Some interference with activity not requiring medical intervention	Prevents daily activity and requires medical intervention	ER visit or hospitalization

5

Contains Nonbinding Recommendations

B. Tables for Laboratory Abnormalities

The laboratory values provided in the tables below serve as guidelines and are dependent upon institutional normal parameters. Institutional normal reference ranges should be provided to demonstrate that they are appropriate.

Serum *	Mild (Grade 1)	Moderate (Grade 2)	Severe (Grade 3)	Potentially Life Threatening (Grade 4)**
Sodium – Hyponatremia mEq/L	132 – 134	130 – 131	125 – 129	< 125
Sodium – Hypernatremia mEq/L	144 – 145	146 – 147	148 – 150	> 150
Potassium – Hyperkalemia mEq/L	5.1 – 5.2	5.3 – 5.4	5.5 – 5.6	> 5.6
Potassium – Hypokalemia mEq/L	3.5 – 3.6	3.3 – 3.4	3.1 – 3.2	< 3.1
Glucose – Hypoglycemia mg/dL	65 – 69	55 – 64	45 – 54	< 45
Glucose – Hyperglycemia<br>Fasting – mg/dL<br>Random – mg/dL	 100 – 110<br>110 – 125	 111 – 125<br>126 – 200	 >125<br>>200	Insulin requirements or hyperosmolar coma
Blood Urea Nitrogen BUN mg/dL	23 – 26	27 – 31	> 31	Requires dialysis
Creatinine – mg/dL	1.5 – 1.7	1.8 – 2.0	2.1 – 2.5	> 2.5 or requires dialysis
Calcium – hypocalcemia mg/dL	8.0 – 8.4	7.5 – 7.9	7.0 – 7.4	< 7.0
Calcium – hypercalcemia mg/dL	10.5 – 11.0	11.1 – 11.5	11.6 – 12.0	> 12.0
Magnesium – hypomagnesemia mg/dL	1.3 – 1.5	1.1 – 1.2	0.9 – 1.0	< 0.9
Phosphorous – hypophosphatemia mg/dL	2.3 – 2.5	2.0 – 2.2	1.6 – 1.9	< 1.6
CPK – mg/dL	1.25 – 1.5 x ULN***	1.6 – 3.0 x ULN	3.1 –10 x ULN	> 10 x ULN
Albumin – Hypoalbuminemia g/dL	2.8 – 3.1	2.5 – 2.7	< 2.5	--
Total Protein – Hypoproteinemia g/dL	5.5 – 6.0	5.0 – 5.4	< 5.0	--
Alkaline phosphate – increase by factor	1.1 – 2.0 x ULN	2.1 – 3.0 x ULN	3.1 – 10 x ULN	> 10 x ULN
Liver Function Tests –ALT, AST increase by factor	1.1 – 2.5 x ULN	2.6 – 5.0 x ULN	5.1 – 10 x ULN	> 10 x ULN
Bilirubin – when accompanied by any increase in Liver Function Test increase by factor	1.1 – 1.25 x ULN	1.26 – 1.5 x ULN	1.51 – 1.75 x ULN	> 1.75 x ULN
Bilirubin – when Liver Function Test is normal; increase by factor	1.1 – 1.5 x ULN	1.6 – 2.0 x ULN	2.0 – 3.0 x ULN	> 3.0 x ULN
Cholesterol	201 – 210	211 – 225	> 226	---
Pancreatic enzymes – amylase, lipase	1.1 – 1.5 x ULN	1.6 – 2.0 x ULN	2.1 – 5.0 x ULN	> 5.0 x ULN

* The laboratory values provided in the tables serve as guidelines and are dependent upon institutional normal parameters. Institutional normal reference ranges should be provided to demonstrate that they are appropriate.

** The clinical signs or symptoms associated with laboratory abnormalities might result in characterization of the laboratory abnormalities as Potentially Life Threatening (Grade 4). For example. a low sodium value that falls within a grade 3 parameter (125-129 mE/L) should be recorded as a grade 4 hyponatremia event if the subject had a new seizure associated with the low sodium value.

***ULN" is the upper limit of the normal range.

Contains Nonbinding Recommendations

Hematology *	Mild (Grade 1)	Moderate (Grade 2)	Severe (Grade 3)	Potentially Life Threatening (Grade 4)
Hemoglobin (Female) - gm/dL	11.0 – 12.0	9.5 – 10.9	8.0 – 9.4	< 8.0
Hemoglobin (Female) change from baseline value - gm/dL	Any decrease – 1.5	1.6 – 2.0	2.1 – 5.0	> 5.0
Hemoglobin (Male) - gm/dL	12.5 – 13.5	10.5 – 12.4	8.5 – 10.4	< 8.5
Hemoglobin (Male) change from baseline value – gm/dL	Any decrease – 1.5	1.6 – 2.0	2.1 – 5.0	> 5.0
WBC Increase - cell/mm^3	10,800 – 15,000	15,001 – 20,000	20,001 – 25, 000	> 25,000
WBC Decrease - cell/mm^3	2,500 – 3,500	1,500 – 2,499	1,000 – 1,499	< 1,000
Lymphocytes Decrease - cell/mm^3	750 – 1,000	500 – 749	250 – 499	< 250
Neutrophils Decrease - cell/mm^3	1,500 – 2,000	1,000 – 1,499	500 – 999	< 500
Eosinophils - cell/mm^3	650 – 1500	1501 - 5000	> 5000	Hypereosinophilic
Platelets Decreased - cell/mm^3	125,000 – 140,000	100,000 – 124,000	25,000 – 99,000	< 25,000
PT – increase by factor (prothrombin time)	1.0 – 1.10 x ULN**	1.11 – 1.20 x ULN	1.21 – 1.25 x ULN	> 1.25 ULN
PTT – increase by factor (partial thromboplastin time)	1.0 – 1.2 x ULN	1.21 – 1.4 x ULN	1.41 – 1.5 x ULN	> 1.5 x ULN
Fibrinogen increase - mg/dL	400 – 500	501 – 600	> 600	--
Fibrinogen decrease - mg/dL	150 – 200	125 – 149	100 – 124	< 100 or associated with gross bleeding or disseminated intravascular coagulation (DIC)

* The laboratory values provided in the tables serve as guidelines and are dependent upon institutional normal parameters. Institutional normal reference ranges should be provided to demonstrate that they are appropriate.

** "ULN" is the upper limit of the normal range.

Urine *	Mild (Grade 1)	Moderate (Grade 2)	Severe (Grade 3)	Potentially Life Threatening (Grade 4)
Protein	Trace	1+	2+	Hospitalization or dialysis
Glucose	Trace	1+	2+	Hospitalization for hyperglycemia
Blood (microscopic) – red blood cells per high power field (rbc/hpf)	1 - 10	11 – 50	> 50 and/or gross blood	Hospitalization or packed red blood cells (PRBC) transfusion

* The laboratory values provided in the tables serve as guidelines and are dependent upon institutional normal parameters. Institutional normal reference ranges should be provided to demonstrate that they are appropriate.

Contains Nonbinding Recommendations

IV. REFERENCES

1. National Cancer Institute Common Toxicity Criteria, April 30, 1999. (http://ctep.cancer.gov/reporting/CTC-3.html)

2. Division of AIDS Table for Grading Severity of Adult Adverse Experiences; August 1992. (http://rcc.tech-res-intl.com/tox_tables.htm)

3. The Brighton Collaboration. Finalized Case Definitions and Guidelines. (http://brightoncollaboration.org/internet/en/index/definition___guidelines.html)

4. HIV Vaccine Trials Network Table for Grading Severity of Adverse Experiences; September 18, 2002. (http://rcc.tech-res-intl.com/tox_tables.htm)

5. Division of AIDS Table for Grading the Severity of Adult and Pediatric Adverse Events, December 2004. (http://www3.niaid.nih.gov/research/resources/DAIDSClinRsrch/PDF/Safety/DAIDSAEGradingTable.pdf)

6. Kratz A, Ferraro M, Sluss PM, Lewandrowski KB. Laboratory Reference Values. New England Journal of Medicine. 2004;351:1548-1563.

14.3 APPENDIX 3: Injection site biopsy sub study

All subjects who provide written informed consent at Screening will have a skin biopsy collected 48 hours following the third vaccination at Visit 10. If a patient has been randomised to receive 2 injections, only one injection site will have a biopsy taken.

Purpose of the Optional biopsy sub study

The vaccine has been demonstrated to produce a reddened thickening at the site of administration which appears after 24 hours and is maximal at 48 hours. This is characteristic of a delayed type hypersensitivity reaction, which would indicate induction of an appropriate antigen specific cell mediated immune response. However, it could represent some other vaccine induced non-specific or inflammatory response. To establish the nature of the response, immunohistochemistry of the lesion at 48 hours may provide evidence of a T-cell infiltrate, oedema and or a neutrophilic infiltrate which would not be associated with antigen specific immunity.

Punch Biopsy Procedure

Equipment and materials:

- Skin cleanser or equivalent (please note the area may be sensitive to touch so alcohol based solutions should be used sparingly),
- Local anaesthetic to numb the area,
- Biopsy punch,
- Dressing,
- Gauze,
- Scalpel,
- Forceps,
- Gloves,
- Sharps container

Procedure

1. Ensure informed consent has been obtained and documented,
2. Thoroughly clean the skin that will be biopsied using a circular motion from the centre of the injection site outwards being careful not to further irritate the skin but ensuring sterile conditions.
3. Administer local anaesthetic per local guidelines to the area. This will be injected just under the epidermis and should create a ‘bleb’ under the skin greater than 3 mm in diameter.

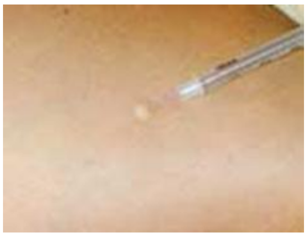

4. Check the local anaesthetic has taken effect. The test site should be positioned around the periphery of the bleb directly over the vaccination site reaction. It may be normal for the

subject to feel a pressure sensation however pain should not be felt. If the subject experiences pain, administer further anaesthesia until the skin is completely numb.

5. A sterile 3 mm skin punch is used to obtain the punch. Pressure is applied and the device is twisted and gently pushed until the blade of the skin punch pierces the epidermis of the skin. About half of the blade should be exposed.

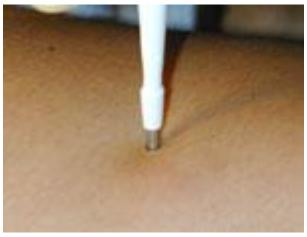

6. The blade will carve out a 3mm cylinder of skin much like a hole punch. Wipe excess blood with gauze to enable the cylinder to be viewed.

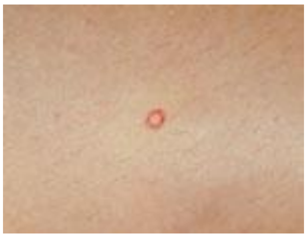

7. Using forceps gently lift the top of the cored skin raising it up so that excess dermis and subdermal fat is visible.

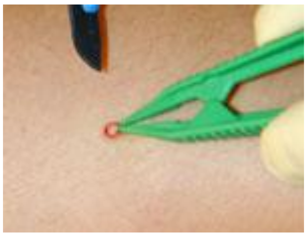

8. With the scalpel in a lateral position under the forceps, gently move the sample towards the scalpel and using one or two strokes excise the biopsy cleanly

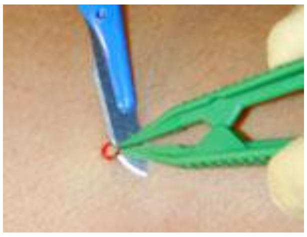

9. Cover the biopsy site with gauze immediately to gently place pressure on the site to help stop bleeding. The site can then be covered with a dressing.

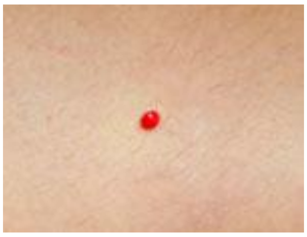

10. Instruct subject to keep the biopsy site clean and dry. The dressing should be changed at least daily.
11. Prepare sample for shipment to laboratory. Please refer to the Study Reference Manual.
