# Supplementary figures and images for: Immune responses to a HSV-2 polynucleotide immunotherapy COR-1 in HSV-2 positive subjects: A randomized double blinded phase I/IIa trial
Source: PLoS One. 2019 Dec 17;14(12):e0226320. doi: 10.1371/journal.pone.0226320 (PMC6917347; doi:10.1371/journal.pone.0226320)

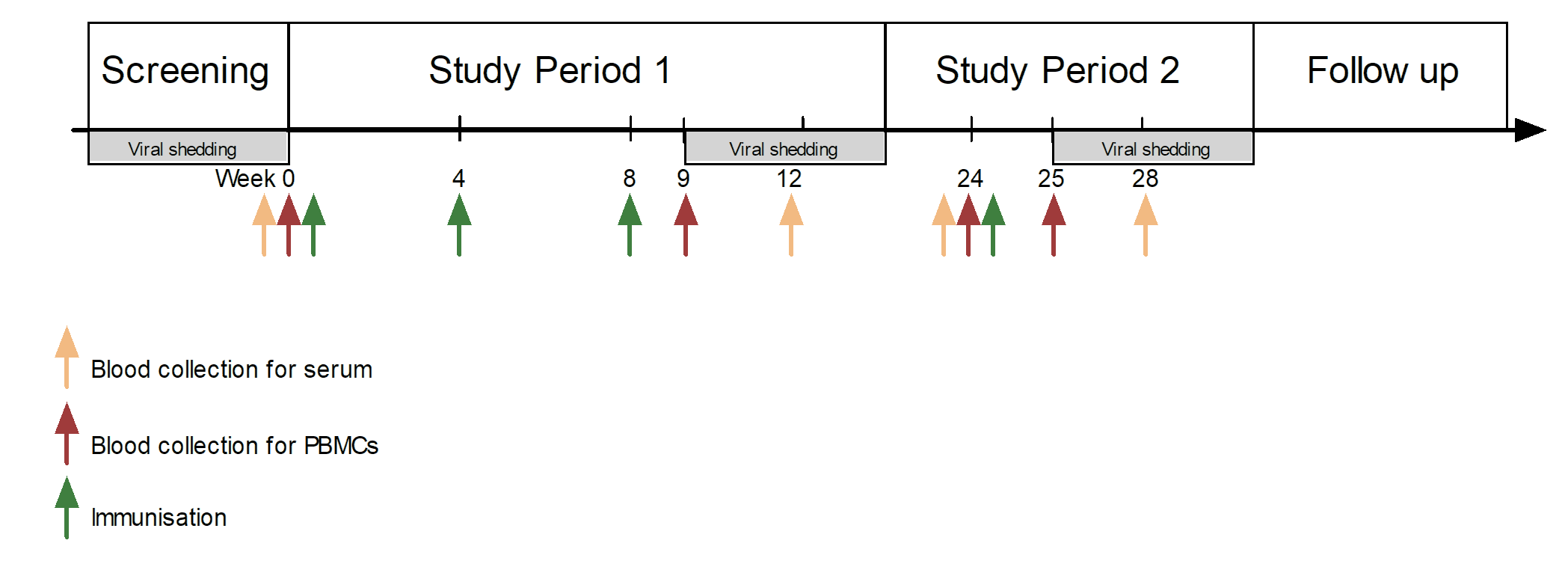

Supplement: S1 Fig — (TIF) [file pone.0226320.s003.tif]
