# Supplementary material for: Immune responses to a HSV-2 polynucleotide immunotherapy COR-1 in HSV-2 positive subjects: A randomized double blinded phase I/IIa trial
Source: PLoS One. 2019 Dec 17;14(12):e0226320. doi: 10.1371/journal.pone.0226320 (PMC6917347; doi:10.1371/journal.pone.0226320)
Supplement: S1 Table — (DOCX) [file pone.0226320.s004.docx]

S1 Table. HSV-2 History.

|  | Group 1 | | Group 2 | | Combined | |
| --- | --- | --- | --- | --- | --- | --- |
|  | COR-1  (N=17) | Placebo  (N=5) | COR-1  (N=17) | Placebo  (N=5) | COR-1  (N=34) | Placebo  (N=10) |
| HSV-2 recurrences / outbreaks in past 12 months | | | | | | |
| Mean (SD) | 5.2 (2.01) | 6.6 (2.30) | 6.5 (1.77) | 5.8 (2.59) | 5.9 (1.99) | 6.2 (2.35) |
| Median | 5.0 | 6.0 | 6.0 | 5.0 | 6.0 | 5.5 |
| Range | 3-9 | 4-9 | 3-9 | 3-9 | 3-9 | 3-9 |
| Sites of recurrent lesions, n (%) | | | | | | |
| Genital | 17 (100) | 5 (100) | 16 (94.1) | 4 (80.0) | 33 (97.1) | 9 (90.0) |
| Perianal | 1 (5.9) | 0 | 5 (29.4) | 0 | 6 (17.6) | 0 |
| Other below waist | 2 (11.8) | 1 (20.0) | 3 (17.6) | 1 (20.0) | 5 (14.7) | 2 (20.0) |
| Subject currently on antiviral therapy, n (%) | | | | | | |
| Yes | 1 (5.9) | 0 | 0 | 0 | 1 (2.9) | 0 |
| No | 16 (94.1) | 5 (100) | 17 (100) | 5 (100) | 33 (97.1) | 10 (100) |
| Has the subject ever taken antiviral therapy for prevention of recurrences? | | | | | | |
| Yes | 11 (64.7) | 4 (80.0) | 15 (88.2) | 5 (100) | 26 (76.5) | 9 (90.0) |
| No | 6 (35.3) | 1 (20.0) | 2 (11.8) | 0 | 8 (23.5) | 1 (10.0) |
| Reason for ceasing antiviral treatment | | | | | | |
| Intolerance | 0 | 0 | 0 | 0 | 0 | 0 |
| Lack of efficacy | 2 (18.2) | 0 | 2 (13.3) | 0 | 4 (15.4) | 0 |
| Patient preference | 4 (36.4) | 2 (50.0) | 4 (26.7) | 3 (60.0) | 8 (30.8) | 5 (55.6) |
| To participate in this study | 5 (45.5) | 2 (50.0) | 8 (53.3) | 2 (40.0) | 13 (50.0) | 4 (44.4) |
| Other | 0 | 0 | 1 (6.7) | 0 | 1 (3.8) | 0 |

Note: SD = standard deviation; Sites of Recurrent Lesions: each subject could have more than one site; Reason for Ceasing Antiviral Treatment: Percentage is based on ‘Subjects Ever Taken Antiviral Therapy for Prevention of Recurrences’.
