# Supplementary material for: Immune responses to a HSV-2 polynucleotide immunotherapy COR-1 in HSV-2 positive subjects: A randomized double blinded phase I/IIa trial
Source: PLoS One. 2019 Dec 17;14(12):e0226320. doi: 10.1371/journal.pone.0226320 (PMC6917347; doi:10.1371/journal.pone.0226320)
Supplement: S2 Table — (DOCX) [file pone.0226320.s005.docx]

**S2 Table. Demographics.**

|  | Group 1 | | Group 2 | | Combined | |
| --- | --- | --- | --- | --- | --- | --- |
|  | COR-1  **(N=17)** | Placebo  (N=5) | COR-1  (N=17) | Placebo  (N=5) | COR-1  (N=34) | Placebo  (N=10) |
| Gender, n (%) |  |  |  |  |  |  |
| Male | 9 (52.9) | 2 (40.0) | 4 (23.5) | 4 (80.0) | 13 (38.2) | 6 (60.0) |
| Female | 8 (47.1) | 3 (60.0) | 13 (76.5) | 1 (20.0) | 21 (61.8) | 4 (40.0) |
| Age (years) |  |  |  |  |  |  |
| Mean (SD) | 33.6 (7.92) | 33.4 (10.11) | 33.4 (7.07) | 35.2 (8.35) | 33.5 (7.39) | 34.3 (8.79) |
| Median | 32.0 | 27.0 | 34.0 | 32.0 | 32.5 | 30.5 |
| Range | 20-48 | 25-48 | 23-45 | 28-48 | 20-48 | 25-48 |
| Weight (kg) |  |  |  |  |  |  |
| Mean (SD) | 77.92 (14.020) | 70.68 (13.405) | 72.14 (12.876) | 84.26 (15.415) | 75.03 (13.576) | 77.47 (15.385) |
| Median | 80.00 | 75.40 | 70.00 | 91.20 | 72.05 | 80.25 |
| Range | 51.7-108.9 | 54.2-85.7 | 56.4-97.0 | 58.5-95.7 | 51.7-108.9 | 54.2-95.7 |
| BMI (kg/m^2^) |  |  |  |  |  |  |
| Mean (SD) | 25.98 (3.834) | 23.98 (3.845) | 24.55 (3.200) | 27.24 (5.866) | 25.27 (3.552) | 25.61 (4.982) |
| Median | 25.90 | 23.80 | 23.90 | 27.00 | 24.90 | 26.15 |
| Range | 20.2-34.0 | 19.4-29.3 | 19.7-30.8 | 19.1-35.6 | 19.7-34.0 | 19.1-35.6 |
| Race, n (%) |  |  |  |  |  |  |
| Asian | 3 (17.6) | 0 | 2 (11.8) | 0 | 5 (14.7) | 0 |
| White | 14 (82.4) | 4 (80.0) | 14 (82.4) | 5 (100) | 28 (82.4) | 9 (90.0) |
| Other | 0 | 1 (20.0) | 1 (5.9) | 0 | 1 (2.9) | 1 (10.0) |
| Ethnicity, n (%) |  |  |  |  |  |  |
| Not Hispanic or Latino | 17 (100) | 5 (100) | 16 (94.1) | 5 (100) | 33 (97.1) | 10 (100) |
| Not reported | 0 | 0 | 1 (5.9) | 0 | 1 (2.9) | 0 |
