# Supplementary material for: Immune responses to a HSV-2 polynucleotide immunotherapy COR-1 in HSV-2 positive subjects: A randomized double blinded phase I/IIa trial
Source: PLoS One. 2019 Dec 17;14(12):e0226320. doi: 10.1371/journal.pone.0226320 (PMC6917347; doi:10.1371/journal.pone.0226320)
Supplement: S3 Table — (DOCX) [file pone.0226320.s006.docx]

**S3 Table: Study schedule of events.**

| Assessments | Screening/Run in | | | | Study Period 1 | | | | | | | | | |
| --- | --- | --- | --- | --- | --- | --- | --- | --- | --- | --- | --- | --- | --- | --- |
|  | Visit 1 | Visit 2 | Visit 3 | Visit 4 | Visit 5 | Visit 6 | Visit 7 | Visit 8 | Visit 9 | Visit 10 | Visit 11 | Visit 12 | Visit 13 | Visit 14 |
|  | Day -46 to -73 | Day  -45 | Day  -43 | Day  -38 | Baseline  D0 | D7 | D28 | D35 | W8 | W8 +48 hours | W9 | W12 | W15 | W19 |
| Informed consent | X |  |  |  |  |  |  |  |  |  |  |  |  |  |
| Review inclusion/exclusion criteria | X |  |  |  | X |  |  |  |  |  |  |  |  |  |
| Demographics, Medical/Surgical History | X |  |  |  |  |  |  |  |  |  |  |  |  |  |
| Physical Examination (symptom directed) | X |  |  |  | (X) | (X) | (X) | (X) | (X) | (X) | (X) | (X) | (X) |  |
| Vital signs/Body Weight/Height | X |  |  |  | X |  | X |  | X |  |  | X |  |  |
| Electrocardiogram | X |  |  |  |  |  |  |  |  |  |  |  |  |  |
| Urine Pregnancy Test | X |  |  |  | X |  | X |  | X |  |  |  |  |  |
| Haematology | X |  |  |  | X | X | X | X | X |  | X | X |  |  |
| Chemistry | X |  |  |  | X | X | X | X | X |  | X | X |  |  |
| Coagulation | X |  |  |  |  |  |  |  |  |  |  |  |  |  |
| Urinalysis | X |  |  |  | X |  | X |  | X |  |  |  |  |  |
| Viral Serology (HIV, Hepatitis B and C, HSV-1 and | X |  |  |  |  |  |  |  |  |  |  |  |  |  |
| ELISA (antibody) |  |  |  |  | X |  | X |  | X |  |  | X |  |  |
| ELIspot (T-cell) |  |  |  |  | X | X |  | X |  |  | X |  |  |  |
| Exploratory Immune assays (T-cell and/or genetic testing) |  |  |  |  | X |  |  |  | X |  | X |  |  |  |
| Randomization |  |  |  |  | X |  |  |  |  |  |  |  |  |  |
| Phone call |  |  | X | X |  |  |  |  |  |  |  |  |  | X |
| Study vaccine administration and bleb check/measurement |  |  |  |  | X |  | X |  | X |  |  |  |  |  |
| Collection of daily swabs from mucocutaneous genital sites for viral shedding for 45 days |  | X |  |  |  |  |  |  |  |  | X |  |  |  |
| Take photos of injection site reactions |  |  |  |  |  | X |  | X |  | X |  |  |  |  |
| Issue and collect Diary |  | X |  |  | X | X | X | X | X |  | X |  | X |  |
| Review diary cards for compliance and adverse events. |  |  | X | X | X | X |  | X |  | X | X | X | X |  |
| Optional skin biopsy |  |  |  |  |  |  |  |  |  | X |  |  |  |  |
| Patient education regarding swab collection, diary completion and outbreak management |  | X | X | X |  |  |  |  |  |  | X | X |  |  |
| Adverse Event and Outbreak assessment |  | X | X | X | X | X | X | X | X | X | X | X | X | X |
| Concurrent Medications | X | X | X | X | X | X | X | X | X | X | X | X | X | X |

| Assessments | Study Period 2 | | | | | Follow up | | |
| --- | --- | --- | --- | --- | --- | --- | --- | --- |
|  | Visit 15 | Visit 16 | Visit 17 | Visit 18 | Visit 19 | Visit 20 | Visit 21 | Visit 22 |
|  | W23 | W24 | W25 | W28 | W31 | W36 | W42 | End of Study/ W48 |
| Informed consent |  |  |  |  |  |  |  |  |
| Review inclusion/exclusion criteria |  |  |  |  |  |  |  |  |
| Demographics, Medical/Surgical History |  |  |  |  |  |  |  |  |
| Physical Examination (symptom directed) | (X) | (X) | (X) | (X) | (X) |  |  | (X) |
| Vital signs/Body Weight/Height |  | X |  | X |  |  |  | X |
| Electrocardiogram |  |  |  |  |  |  |  |  |
| Urine Pregnancy Test |  | X |  |  |  |  |  | X |
| Hematology | X | X | X | X |  |  |  | X |
| Chemistry | X | X | X | X |  |  |  | X |
| Coagulation |  |  |  |  |  |  |  |  |
| Urinalysis |  | X |  |  |  |  |  | X |
| Viral Serology (HIV, Hepatitis B and C, HSV-1 and 2) |  |  |  |  |  |  |  |  |
| Competitive Inhibition ELISA (antibody) |  | X |  | X |  |  |  | X |
| ELIspot (T cell) |  | X | X |  |  |  |  | X |
| Exploratory Immune assays (T cell and/or genetic testing) |  | X | X |  |  |  |  | X |
| Randomization |  |  |  |  |  |  |  |  |
| Phone call |  |  |  |  |  | X | X |  |
| Study vaccine administration and bleb check/measurement |  | X |  |  |  |  |  |  |
| Collection of daily swabs from mucocutaneous genital sites for viral shedding for 45 days |  |  | X |  |  |  |  |  |
| Take photos of injection site (s) |  |  | X |  |  |  |  |  |
| Issue and collect Diary |  | X | X |  | X |  |  |  |
| Review diary cards for compliance and adverse events. |  |  | X | X | X |  |  |  |
| Skin biopsy |  |  |  |  |  |  |  |  |
| Patient education regarding swab collection, diary completion and outbreak management |  |  | X | X |  |  |  |  |
| Adverse Event and Outbreak assessment | X | X | X | X | X | X | X | X |
| Concurrent Medications | X | X | X | X | X | X | X | X |
