# Supplementary material for: Immune responses to a HSV-2 polynucleotide immunotherapy COR-1 in HSV-2 positive subjects: A randomized double blinded phase I/IIa trial
Source: PLoS One. 2019 Dec 17;14(12):e0226320. doi: 10.1371/journal.pone.0226320 (PMC6917347; doi:10.1371/journal.pone.0226320)
Supplement: S4 Table — (DOCX) [file pone.0226320.s007.docx]

**S4 Table. Summary of Treatment-Emergent Adverse Events occurring in ≥1 subject by system organ class (Safety Population).**

| **System Organ Class** |  | | | |
| --- | --- | --- | --- | --- |
|  | COR-1  (N=34) | | Placebo  (N=10) | |
|  | No. of Subjects (%) | No. of Events | No. of Subjects (%) | No. of Events |
| **All Body Systems** | **34**  **(100.0)** | **661** | **9**  **(90.0)** | **125** |
| **Gastrointestinal disorders** | **9**  **(26.5)** | **13** | **3**  **(30.0)** | **7** |
| Diarrhea | 3 (8.8) | 3 | 1 (10.0) | 2 |
| Nausea | 3 (8.8) | 3 | 1 (10.0) | 1 |
| Vomiting | 2 (5.9) | 2 | 0 | 0 |
| **General disorders and administration site conditions** | **34**  **(100.0)** | **483** | **9**  **(90.0)** | **58** |
| Injection site discoloration | 15 (44.1) | 43 | 0 | 0 |
| Injection site vesicles | 2 (5.9) | 3 | 0 | 0 |
| Vaccination site bruising | 2 (5.9) | 3 | 0 | 0 |
| Vaccination site erythema | 34  (100.0) | 229 | 8  (80.0) | 34 |
| Vaccination site induration | 28 (82.4) | 133 | 6 (60.0) | 17 |
| Vaccination site pain | 11 (32.4) | 42 | 2 (20.0) | 4 |
| Vaccination site pruritus | 10 (29.4) | 26 | 0 | 0 |
| **Infections and infestations** | **29 (85.3)** | **109** | **9 (90.0)** | **32** |
| Herpes simplex | 21 (61.8) | 64 | 8 (80.0) | 20 |
| Upper respiratory tract infection | 20 (58.8) | 36 | 7 (70.0) | 8 |
| **Injury, poisoning and procedural complications** | **4 (11.8)** | **5** | **5 (50.0)** | **6** |
| Contusion | 2 (5.9) | 2 | 3 (30.0) | 3 |
| **Investigations** | **1 (2.9)** | **2** | **1 (10.0)** | **3** |
| Aspartate aminotransferase increased | 1 (2.9) | 1 | 1 (10.0) | 1 |
| Blood creatine phosphokinase increased | 1 (2.9) | 1 | 1 (10.0) | 1 |
| **Musculoskeletal and connective tissue disorders** | **6 (17.6)** | **6** | **4 (40.0)** | **5** |
| Arthralgia | 0 | 0 | 2 (20.0) | 2 |
| Back pain | 2 (5.9) | 2 | 1 (10.0) | 1 |
| Bursitis | 1 (2.9) | 1 | 1 (10.0) | 1 |
| **Nervous system disorders** | **12 (35.3)** | **20** | **4 (40.0)** | **6** |
| Dizziness | 2 (5.9) | 2 | 0 | 0 |
| Headache | 10 (29.4) | 15 | 3 (30.0) | 3 |
| **Psychiatric disorders** | **3 (8.8)** | **4** | **0** | **0** |
| Insomnia | 2 (5.9) | 3 | 0 | 0 |
| **Respiratory, thoracic and mediastinal disorders** | **6 (17.6)** | **10** | **2 (20.0)** | **4** |
| Oropharyngeal pain | 5 (14.7) | 7 | 2 (20.0) | 3 |
| Rhinorrhea | 2 (5.9) | 2 | 0 | 0 |
