# Supplementary material for: Immune responses to a HSV-2 polynucleotide immunotherapy COR-1 in HSV-2 positive subjects: A randomized double blinded phase I/IIa trial
Source: PLoS One. 2019 Dec 17;14(12):e0226320. doi: 10.1371/journal.pone.0226320 (PMC6917347; doi:10.1371/journal.pone.0226320)
Supplement: S5 Table — (DOCX) [file pone.0226320.s008.docx]

**S5 Table. Swabs collected for early withdrawals.**

| **Subject** | **Swab collection period** | **Days swabs collected** | **Shedding rate calculated** | **Study arm** |
| --- | --- | --- | --- | --- |
| S016 | Screening | 42 | Yes | Group 1 COR-1 |
|  | Post vaccination |  | No |  |
|  | Post Booster |  | No |  |
| S017 | Screening | 45 | Yes |  |
|  | Post vaccination |  | No |  |
|  | Post Booster |  | No |  |
| S018 | Screening | 44 | Yes |  |
|  | Post vaccination |  | No |  |
|  | Post Booster |  | No |  |
| S033 | Screening | 45 | Yes |  |
|  | Post vaccination |  | No |  |
|  | Post Booster |  | No |  |
| S037 | Screening | 45 | Yes |  |
|  | Post vaccination | 48 | Yes |  |
|  | Post Booster |  | No |  |
| S054 | Screening | 48 | Yes |  |
|  | Post vaccination |  | No |  |
|  | Post Booster |  | No |  |
| S119 | Screening | 45 | Yes |  |
|  | Post vaccination | 5 | Yes |  |
|  | Post Booster |  | No |  |
| S100 | Screening | 45 | Yes | Group 2 Placebo |
|  | Post vaccination |  | No |  |
|  | Post Booster |  | No |  |
